# Parallel and non-parallel features of adaptive radiation in Yucatán pupfishes

**DOI:** 10.1101/2025.11.17.688971

**Published:** 2025-11-18

**Authors:** Matthew C. Kustra, David Tian, M. Fernanda Palominos, Feifei Guo, Dylan Chau, Oskar Golwala, HoWan Chan, Andrés Alvarez Zapata, Reyna Guadalupe Cetz Paredes, Frida Ximena Cortés Sánchez, Sonia Gabriela Hernández, Adan Fernando Mar-Silva, Fernando Mex, Charles Tralka, Maribel Badillo-Alemán, Juan J. Schmitter-Soto, Carlos A. Gracida-Juárez, Christopher M. Martinez, Jairo Arroyave, Christopher H. Martin

**Affiliations:** 1 Department of Integrative Biology, University of California, Berkeley, CA, United States; 2 Museum of Vertebrate Zoology, University of California, Berkeley, CA, United States; 3 Miller Institute for Basic Research in Science, University of California, Berkeley, CA, United States; 4 Department of BioSciences, Rice University, Houston, TX, United States; 5 Instituto Tecnologico Superior de Conkal, Yucatan, Mexico; 6 Instituto de Biología, Universidad Nacional Autónoma de México, Mexico City, Mexico; 7 Unidad Multidisciplinaria de Docencia e Investigación, Unidad Sisal, Universidad Nacional Autónoma de México, Sisal, Yucatán, Mexico; 8 Departamento de Sistemática y Ecología Acuática, El Colegio de la Frontera Sur, Chetumal, Quintana Roo, Mexico; 9 Subdirección de Posgrado e Investigación, Tecnológico Nacional de México, Campus Felipe Carrillo Puerto.Carretera a Vigía Chico km 1.5, 77200 Felipe Carrillo Puerto, Quintana Roo.; 10 Department of Ecology and Evolutionary Biology, University of California, Irvine, CA, United States

## Abstract

Understanding the extent of parallelism across adaptive radiations remains a central problem in evolutionary biology. We used whole-genome resequencing of 123 individuals to study an adaptive radiation of *Cyprinodon* pupfishes in Laguna Chichancanab, Mexico, compared to previous research on an independent radiation of San Salvador Island (SSI) pupfishes in the Bahamas, to assess the repeatability of their genetic architecture, sources of adaptive variation, and stages of selection. Despite rapid craniofacial divergence of trophic specialists within 8–15 kya, only two candidate genes (3.6%) were shared between Caribbean radiations. Although adaptive introgression played a major role in SSI, we found minimal evidence of adaptive introgression in Chichancanab, likely due to the higher geographical isolation of this inland lake. Instead, de novo mutations provided a substantial source of adaptive variation (30.6%) for the endemic zooplanktivore, nearly 10 times higher than the scale-eater in SSI. We found strong evidence that selection occurred in stages, first on regulatory and standing genetic variation, then on de novo and nonsynonymous mutations, parallel to SSI. Unique to Chichancanab, adaptive variants near opsin and spermatogenesis genes are consistent with our findings of greater visual acuity and differences in sperm morphology, respectively, within the zoooplanktivore. Our study suggests that de novo mutations may contribute more to rapid adaptive radiations than previously appreciated, particularly in isolated environments.

## Introduction

Much of biodiversity results from adaptive radiation—when a clade diversifies rapidly into three or more new species (1–5). Young adaptive radiations provide excellent systems for investigating speciation and adaptation (3, 6–9). However, it remains unclear to what degree radiations occur in parallel given similar starting positions of genetic diversity, gene flow, and environments (10, 11).

Beneficial de novo mutations may provide novel variation to reach new fitness peaks on the adaptive landscape during adaptive radiations (12, 13); however, the time needed for de novo beneficial mutations to arise contrasts with the rapid divergence of adaptive radiations (11, 14, 15). Alternatively, hybridization could introduce large amounts of genetic variation and novel phenotypes (transgressive segregation) through hybrid swarms, enabling populations to reach previously inaccessible fitness peaks (16). Support for the role of hybridization in adaptive radiations has been demonstrated both theoretically (17, 18) and empirically in many systems (3, 19–27). However, we still do not know the importance of different sources of genetic variation across time and space.

Caribbean pupfishes are a remarkable system for assessing the repeatability of adaptive radiations due to two independent, recent adaptive radiations that contain a generalist and multiple trophic specialists within isolated lake environments that have historically lacked predators. The San Salvador Island (SSI), Bahamas radiation proceeded predominantly via the reassembly of pre-existing standing genetic variation found throughout the Caribbean, with additional contributions of adaptive introgression in both molluscivore (durophage) and scale-eating (lepidophage) specialists, and an additional 2% of de novo mutations within the scale-eater (26, 28–32). However, it is unclear how a second radiation in Laguna Chichancanab, Mexico, drawing from the same Caribbean pool of standing genetic variation over the same timeframe unfolded, allowing us to assess the degree to which radiations are parallel.

**Figure 1.**
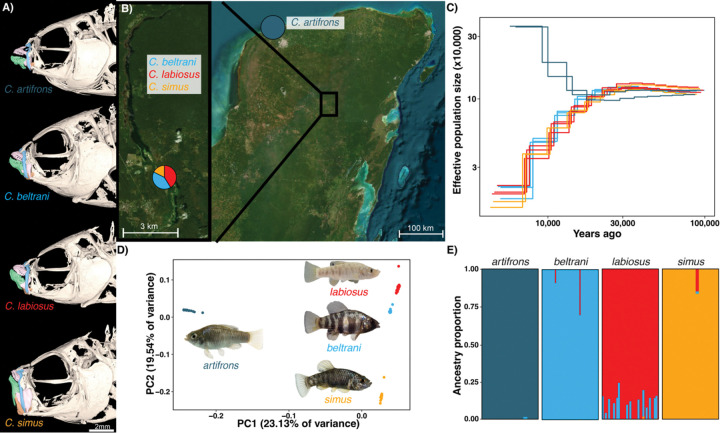
Yucatán *Cyprinodon* pupfishes show strong genetic differentiation. (A) μCT scans of Yucatán pupfish. (B) Google Earth image of the Yucatán Peninsula showing the sampling site for *C. artifrons*, with an inset map of Laguna Chichancanab. The pie chart represents the relative frequency of the different Chichancanab species in our dataset. (C) Estimated demographic history with *MSMC2* using linkage disequilibrium pruned SNPs. Each colored line represents an individual, with colors corresponding to different species. (D) Principal component analysis using a set of linkage disequilibrium pruned SNPs. (E) Estimated ancestry proportions for each individual inferred using *ADMIXTURE* with K = 4 (see Figure S2 for K=2–3) from the linkage-pruned dataset.

Here, we use whole-genome resequencing of 123 individuals to provide insight into the genomic architecture and genetic origins of the Laguna Chichancanab *Cyprinodon* pupfish radiation (33). This recent adaptive radiation (~8000 years old (34)) consisted of at least five species historically (Strecker, 2005; Humphries and Miller 1981), including a detritivore (*C. beltrani*), piscivore (*C. maya*), rocky substrate specialist (*C. verecundus*), bivalve specialist (*C. labiosus*), and zooplanktivore (*C. simus*). However, due to recent colonization by native fishes not historically present in the lake and invasive colonization by African tilapia (*Oreochromis* sp.) (36, 37), all trophic specialist species now appear to be extinct since our collections in 2022, with only the generalist, *C. beltrani*, remaining in 2024 (MK pers. obs.) and 2025 (CG, JSS pers. obs.). Despite exhibiting one of the fastest rates of trait diversification of any vertebrate in tooth length (130 times faster than background rates across *Cyprinodon* (29)) and rapid divergence in other craniofacial morphology (Figure 1A), previous genetic studies of microsatellite data and mtDNA suggest that this radiation has low genetic differentiation (38, 39), potentially enabling the identification of highly differentiated loci among species that may be involved in trophic adaptation.

## Results

### Demographic history

To understand the demographic history and population structure of the Yucatán *Cyprinodon* pupfishes, we collected specimens from the wild in 2022 (Figure 1B) and conducted whole genome resequencing of the species flock and the coastal sister species (*n* = 15 *C. artifrons*, *n* = 35 *C. beltrani*, *n* = 35 *C. labiosus*, and *n* = 17 *C. simus*). We aligned these samples, as well as eleven other previously sequenced Caribbean pupfish species, to the *Cyprindon nevadensis mionectes* reference genome (GCA_030533445.1). After calling and filtering variants, our dataset included 23,960,536 SNPs (12.4x mean depth).

Using MSMC2 on Chichancanab species on a linkage disequilibrium pruned set of SNPs (*n* =5,476,855 SNPs) and a mutation rate of 1.56 × 10^−8^ substitutions per base pair (estimated from a Caribbean *Cyprinodon* species (26)), we found that the three Chichancanab species (*C. beltrani*, *C. labiosus*, and *C. simus*) diverged from the coastal sister species *C. artifrons* at the end of the Pleistocene, 10–15 kya, consistent with receding sea levels resulting in the formation of the Chichancanab lake (34). Since divergence from *C. artifrons*, the Chichancanab species flock shows a similar demographic history, characterized by a sharp decline in effective population sizes, consistent with endemic sympatric species within an isolated lake (Figure 1C).

### Genetic differentiation of the species flock

Using a linkage-pruned set of SNPs and the subset of data restricted to Yucatán pupfish, we used principal component analysis and ADMIXTURE to visualize population structure. We found that all species formed distinct, non-overlapping genetic clusters, with *C. artifrons* being the most genetically divergent from and sister to the Chichancanab species (Figure 1D). Within the Chichancanab species flock, *C. simus* was more genetically divergent from *C. labiosus* and *C. beltrani* (Figure 1D). These results were also supported by genome-wide *F*_*ST*_ estimates and a phylogenetic tree constructed with *ADMIXTOOLS2* (Table S1; Figure S1). Four clusters corresponded to the four different species, as determined by ADMIXTURE (Figure 1E). All *C. artifrons* individuals showed minimal evidence of admixture with Chichancanab species (Figure 1E).

### Adaptive loci within trophic specialists

**Figure 2.**
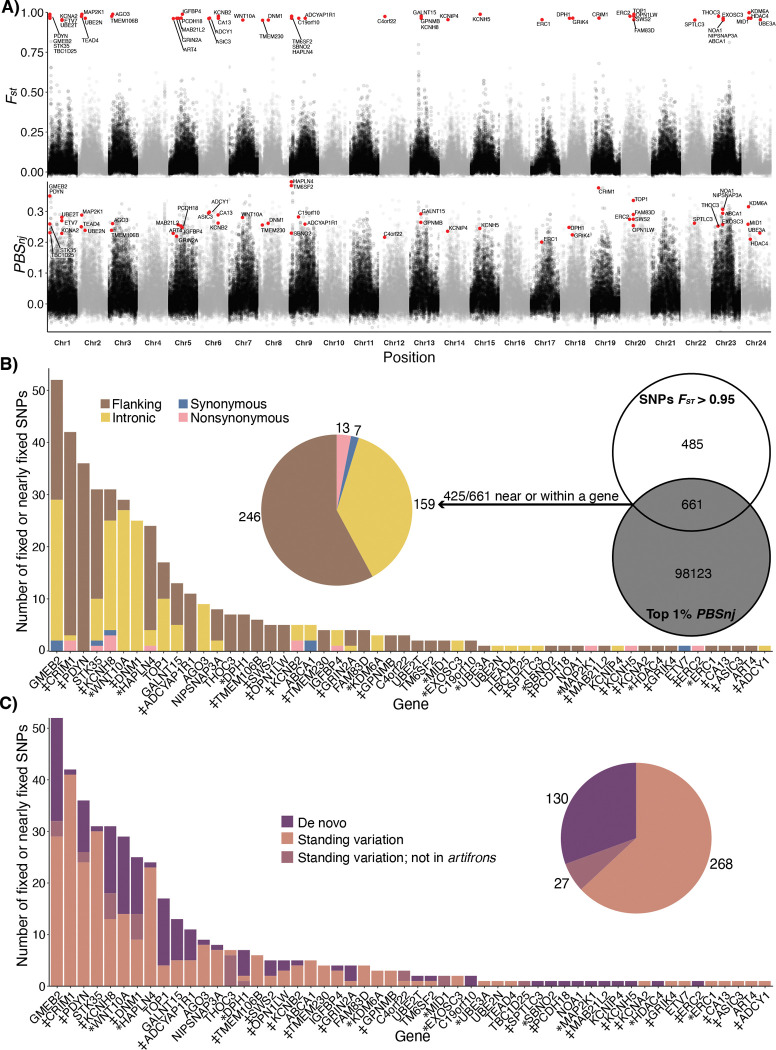
Architecture and source of adaptive genetic variation in the zooplanktivore. (A, top row) *F*_*st*_ between *C. simus* and *C. beltrani* + *C. labiosus* calculated in non-overlapping 10-kb windows. *F*_*st*_ values for candidate SNPs (red circles) were calculated separately per locus to show the location of fixed variants. (A, lower row) Population branch statistics (*PBS*_*nj*_) with *C. simus* as the target population versus *C. beltrani*, *C. labiosus*, and *C. artifrons* in 10-kb non-overlapping windows. Candidate genes within 20 kb of fixed or nearly-fixed (Fst > 0.95) SNPs and within *PBS*_*nj*_ 1% outlier windows are highlighted in red and labeled. Alternating colors represent different chromosomes. (B) Candidate adaptive variants near or within a gene were defined as SNPs that were fixed (*n* = 17) or nearly fixed (*n* = 1,129, *F*_*st*_ > 0.95) and occurred within a 1% population branch statistic (*PBS*_*nj*_) outlier window for *C. simus* (Venn diagram). Pie chart and bar graphs indicate the proportion of these 425 adaptive variants that were found within the flanking, intronic, or coding regions. (C) Pie chart and bar graphs indicate the proportion of these 425 variants that were detected only within Chichancanab (de novo), as standing genetic variation in coastal sister species *C. artifrons*, or in other Caribbean species, but not *C. artifrons*. * Indicates genes with a craniofacial annotation; ‡ indicates genes with neural or sensory annotation.

Although genome-wide differentiation among Chichancanab species was moderately low (*F*_*ST*_ ranging from 0.032 to 0.09; Figure 2A; Table S1), we were able to identify highly differentiated regions of the genome within the trophic specialist zooplanktivore *C. simus*. We identified 19 fixed and 1,127 nearly-fixed (*F*_*ST*_ >0.95) SNPs in *C. simus* relative to other Chichancanab pupfishes (Table S2, 3). In contrast, we found zero fixed or nearly-fixed SNPs within the generalist *C. beltrani* or bivalve-specialist C. *labiosus*. However, we were able to identify some moderately differentiated SNPs within *C. labiosus* (*n* = 97) and *C. beltrani* (*n* = 13) relative to the other Chichancanab species at an *F*_*ST*_ threshold of 0.8 (Table S2).

To search for signals of selection, we calculated the normalized four-taxon population branch statistic (*PBS*_*nj*_; Schmidt et al., 2019) across 10-kb non-overlapping windows because linkage disequilibrium decayed at this distance (Figure S3). Simulation studies demonstrate that normalized *PBS* is effective in identifying both hard and soft selective sweeps (40). Out of the 1,146 SNPs fixed or nearly fixed within *C. simus*, 661 were within a strongly supported selective sweep window (top 1% *PBS*_*nj*_ outlier (40)), including 425 SNPs within 20 kb of the first or last exon of an annotated gene. These 425 candidate adaptive SNPs (“adaptive variants”) were located in the proximity of or within 55 genes (Figure 2A), distributed across 19 out of 24 chromosomes (Figure 2A). Adaptive variants associated with the same gene were often distributed across multiple introns or exons and spaced apart by 1kb or more (Figure S4, Table S4).

### Source and function of adaptive genetic variation in the zooplanktivore

We next characterized the location of the 425 candidate adaptive variants near or within a gene in the zooplanktivore *C. simus*. More than 95% were in intronic or flanking regions (20-kb upstream or downstream of a gene; Figure 2B). Within coding regions, 13 out of 20 adaptive variants were nonsynonymous mutations (Figure 2B). The number of adaptive variants per gene followed an exponential distribution, with a handful of genes containing many variants and most genes with only a single adaptive variant (Figure 2B). We functionally characterized these genes by manually checking online databases and doing a literature search (Table S3; Extended Table S1). 32 out of the 55 candidate genes were associated with craniofacial development (*n* =11; 20%) or sensory/neural function (*n* = 21; 38.2%). We manually checked if any of these genes overlapped with the 176 unique candidate genes identified in the SSI radiation (26), and we found only two overlapping candidate genes (*DNM1* and *SPTLC3*), both of which are associated with motor neuron development (41, 42) and are scale-eater-specific candidate genes.

Next, we characterized the source of the variants and found that 63% of the adaptive SNPs in *C. simus* also existed as standing genetic variation in the coastal Yucatan population of *C. artifrons* (“standing variation”), 31% were *de novo* variants found only in Chichancanab, and 6% were detected as standing genetic variation in other pupfish species but not *C. artifrons* (Figure 2C).

### Limited adaptive introgression within Chichancanab pupfishes

**Figure 3.**
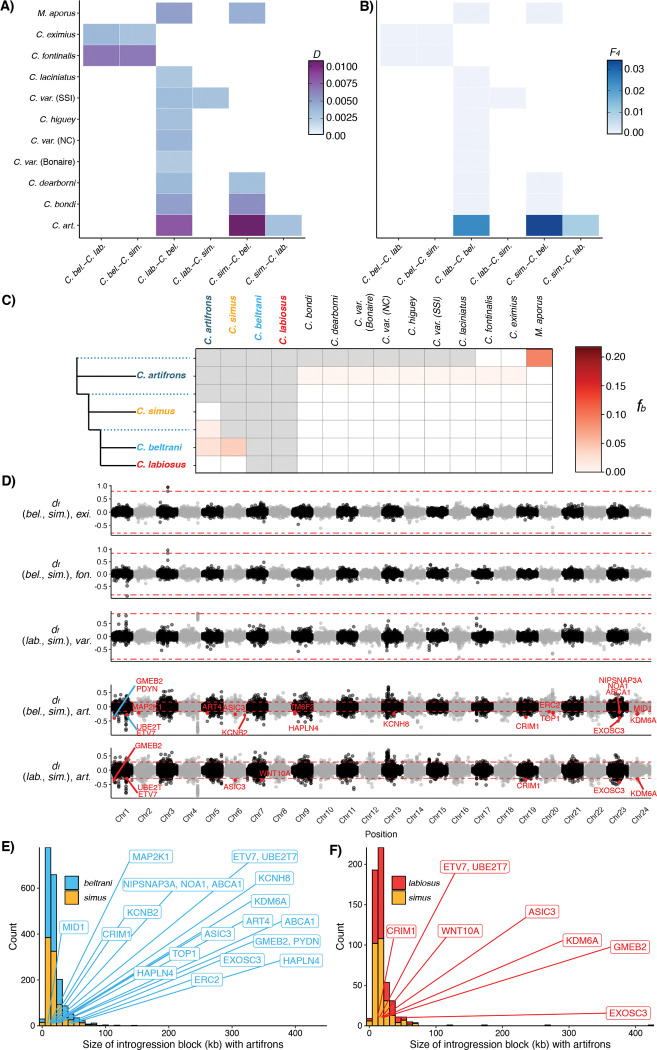
Limited adaptive introgression into *C. simus*. Heatmaps of (A) Patterson’s *D-*statistics and (B) *F*_*4*_ ratios for P1-P2 species combinations within the Chichancanab radiation along the x-axis and P3 focal species along the y-axis. All four-taxon trees used *Cualac tessellatus* as the outgroup (P4). Only significant (*Z*-score > 3) results are colored; combinations not relevant to the Chichancanab radiation are not shown. (C) *f-*branch (*f*_*b*_) statistics indicate introgression occurring before the Chichancanab radiation. Red boxes indicate significant introgression between P3 species (top row) and the focal species or internal branch (dashed lines) on the left. Non-significant *f*_*b*_ statistics are indicated by white boxes. Grey boxes represent comparisons that cannot be made since *f*_*b*_ cannot be calculated for introgression between sister taxa. See Figure S5 for full *f-*branch graphs. (D) Genome-wide plot of the distance fraction (*d*_*f,*_, an introgression test statistic; Pfeifer & Kapan, 2019) calculated in non-overlapping windows of 91 SNPs (approximately 10 kb) for species trios that showed evidence of introgression with *C. simus. d*_*f*_ outlier windows that contained a candidate gene under selection in *C. simus* are colored red and labeled with the gene name. The y-axis label gives the introgression topology: (P1, P2), P3. Positive values indicate regions of introgression between *C. simus* and P3 (from top to bottom: *C. eximius*, *C. fontinalis*, *C. variegatus* (SSI), and *C. artifrons* in the bottom two subplots); negative values indicate regions of introgression between P1 (*C. beltrani* or *C. labiosus*) and P3. Red dashed lines indicate outlier cutoffs, which were determined empirically by the percentage of genome-wide introgression estimated by *F*_*4*_ ratios in panel B. (E, F) Distribution of the size of *d*_*f*_ outlier introgression tract lengths (in 10 kb windows) between *C. artifrons* and Chichancanab species. The introgression tracts containing candidate adaptive genes in *C. simus* are labeled with gene names. SSI: San Salvador Island, Bahamas; NC: North Carolina, USA.

To test for introgression into the Chichancanab radiation, we used *f*-statistics calculated in *Dsuite* (43) to examine gene flow from 11 additional *Cyprinodon* species from across the clade and the sister species to the genus *Megupsilon aporus*. Within Yucatán pupfishes, we found evidence of introgression between *C. artifrons* and both *C. beltrani* and *C. labiosus*, with the strongest signal in *C. beltrani* (Figure 3A, B). We also found evidence for introgression between *C. beltrani* and most outgroup species examined, including the sister species to *Cyprinodon*, *Megupsilon aporus*. Relative to *C. beltrani*, *C. labiosus* and *C. simus* also showed evidence of introgression with pupfishes endemic to the Chihuahua desert (*C. eximius* and *C. fontinalis*; Figure 3A, B). Furthermore, we also found evidence of introgression between the two sympatric radiations (*C. variegatus* from SSI) and *C. simus* (Figure 3A, B). However, additional *f-*branch statistics indicate that all patterns of excess allele sharing between these outgroup species besides *C. artifrons* and the Chichancanab species flock likely result from ancient introgression events arising before the colonization of Laguna Chichancanab (Figure 3C; S5).

To test for adaptive introgression into *C. simus*, we next examined whether the set of adaptive variants (Figure 2) occurred within outlier introgression windows using sliding window scans of distance fraction (*d*_*f*_; Pfeifer & Kapan, 2019) for species that had significant genome-wide introgression into *C. simus* (Figure 3A, B). We found that none of the outlier introgression windows overlapped with *C. simus* adaptive variants (Figure 3D).

Next, we performed *df* scans for introgression between *C. artifrons* and Chichancanab species. Although there were outlier introgression regions between *C. simus* and *C. artifrons* (positive values *d*_*f*_), these regions did not overlap with *C. simus* adaptive variants (Figure 3D). However, the position of several of these *C. simus* adaptive variants was found in outlier introgression windows in *C. labiosus* and *C. beltrani* (Figure 3D). The number of outlier introgression windows was much lower in *C. simus* relative to *C. labiosus* and *C. beltrani* (Figure 3E, F). Furthermore, both *C. labiosus* and *C. beltrani* genomes contained several introgression tracts that were much longer than *C. simus* tract lengths, indicative of much more recent introgression events in these species (Figure 3E, F).

### The stages of adaptive divergence of the zooplanktivore

**Figure 4.**
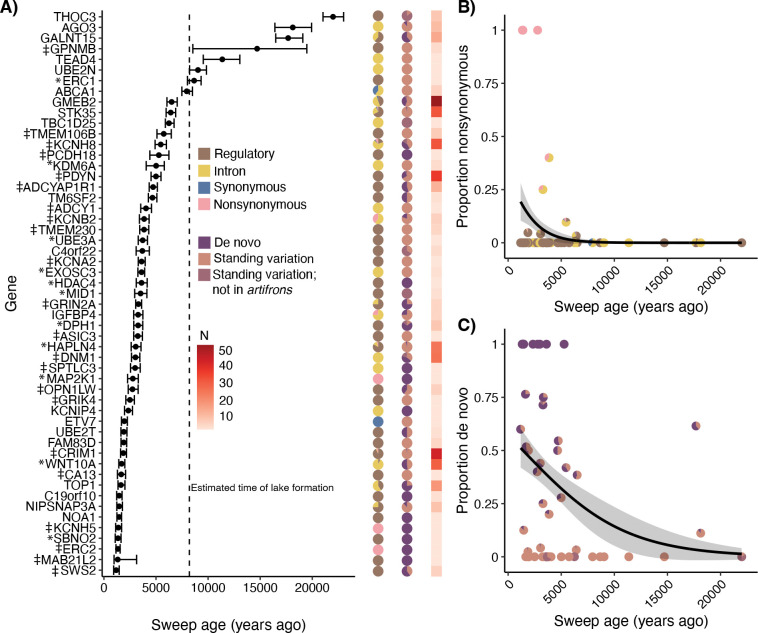
Stages of adaptive divergence in the zooplanktivore. (A) Points indicate median sweep age with 95% credible intervals. The dashed line indicates the approximate age of Laguna Chichancanab (34). For each sweep, the first column of pie charts shows the proportions of flanking/intronic/synonymous/nonsynonymous, second column of pie charts shows the proportions of de novo/standing variation, and third column of squares shows the number of candidate adaptive variants per gene (from Fig. 2). For genes with multiple adaptive variants, we used the variant with the median position as the focal position. * Indicates genes with a craniofacial annotation; ‡ indicates genes with neural or sensory annotation. (B) The proportion of nonsynonymous adaptive variants is significantly larger in more recent selective sweeps, indicated by the best-fit quasibinomial regression line; each pie chart represents a single candidate gene. (C) The proportion of de novo variants is significantly larger in more recent selective sweeps; each pie chart represents a single candidate gene.

**Figure 5.**
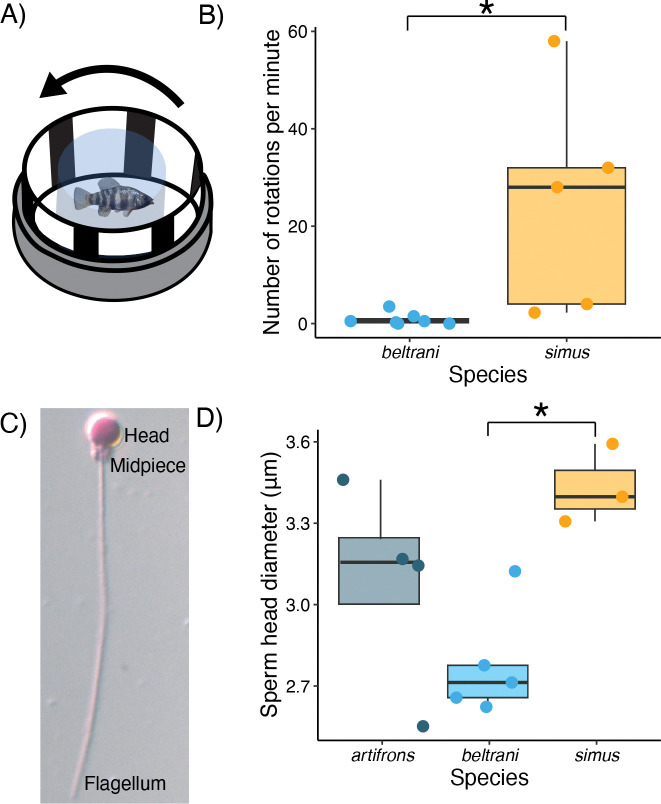
Zooplanktivore *C. simus* has significantly greater visual acuity and divergent sperm morphology relative to the generalist *C. beltrani*. (A) Diagram of the optomotor response experimental apparatus (adapted from Neuhauss et al., 1999). (B) Boxplot and jittered points of the number of complete rotations fish swam per minute, indicative of their optomotor response to black-and-white vertical bars spinning at a constant rate. (C) Representative labeled image of a single *C. beltrani* sperm at 100x stained with rose bengal. (D) Boxplots with jittered points of sperm head diameter for *C. artifrons, beltrani,* and *simus.* Significant Tukey’s post hoc differences are shown with an asterisk.

We used *starTRMCA* (45) to estimate the timing of selective sweeps for the set of candidate adaptive variants near genes within the zooplanktivore *C. simus* (Fig. 4). We estimated that most selective sweeps occurred in a staggered sequence and not simultaneously after colonization of Laguna Chichancanab (Figure 5A)(34). There were also a few older sweeps that we estimate may predate the age of Laguna Chichancanab and divergence from the coastal sister species *C. artifrons* (Figure 1B). Overall, we observed no clear temporal stages of adaptive divergence related to craniofacial genes (asterisks: Figure 5) or neural/sensory genes (‡: Figure 5); instead, we found that selection on these two broad functional categories occurred throughout the period of divergence of *C. simus* following the colonization of Laguna Chichancanab.

Next, we used generalized linear regressions to test if sweep age was correlated with the proportion of nonsynonymous adaptive variants, de novo adaptive variants, or the overall number of adaptive variants per gene region. We found that both nonsynonymous adaptive variants (χ^2^ = 5.852, *p* = 0.016) and de novo mutations (χ^2^ = 6.221, *p* = 0.013) were significantly more likely to occur among the more recent selective sweeps during the final stage of adaptive divergence in *C. simus* relative to other members of the species flock (Figure 5B, C). However, nonsynonymous mutations were not significantly more likely to be de novo mutations (All adaptive variants: χ^2^ = 0.102, *P* = 0.749; singleton adaptive variants: χ^2^ = 2.010, *p* = 0.156), and there was no correlation between estimated sweep age and the number of candidate adaptive variants per gene region (χ^2^ = 0.111, *p* = 0.740).

### Increased visual acuity in the zooplanktivore is consistent with vision-related candidate adaptive genes

We identified many *C. simus* adaptive variants associated with eye development (*ASIC3*, *CRIM1*, *MAB21L2*) and opsin genes (*OPN1LW*, *SWS2*). Because the trophic specialization of *C. simus* requires visually locating zooplankton in the open water before targeted suction-feeding strikes, we hypothesized that this species would have higher visual acuity than the detritivore, *C. beltrani*. We conducted an optomotor response experiment on lab-reared individuals from both species (F1 *C. beltrani* and long-term laboratory colony of *C. simus* (F10+)) and found that *C. simus* has a much stronger optomotor response than *C. beltrani* in response to black-and-white vertical bars spinning at a constant rate of ~89.4 rotations per minute (Figure 5A, B; Wilcoxon signed-rank test: *W* =1, *p* = 0.009). There was no significant difference in rotations per minute between the species for the same individuals during control observation periods of no spinning (Figure S6A; *W*=14, *p* = 0.3105) or during a control treatment with an all-black background spinning at the same frequency (Figure S6B; *W* =9, *p* = 0.147).

### Divergent sperm morphology of the zooplanktivore is consistent with spermatogenesis-related candidate adaptive genes

Four candidate genes (*C4orf22*, *STK35*, *ERC2*, and *TBC1D25*) showed annotations relating to spermatogenesis/fertility. We therefore examined whether three Yucatan species, raised in a common garden laboratory environment (F1 *C. artifrons,* F1 *beltrani,* and a long-term laboratory colony of C. *simus* F10+), differed in sperm morphology. We found that species significantly differed in sperm head length (Figure 5D; *F* = 5.724, *p* = 0.025), but not sperm midpiece length (Figure S7A; *F* = 2.403, *p* = 0.147) or flagellum length (Figure S7B; *F* = 0.414, *p* = 0.673). Tukey’s post hoc pairwise comparisons indicated that *C. simus* had significantly larger sperm heads than *C. beltrani* (*q* = 3.367; *p* = 0.0207; Fig. 5D).

## Discussion

Here, we investigated the genetic architecture, source of adaptive variation, and temporal dynamics of selection underlying speciation and trophic specialization in a young radiation of *Cyprinodon* pupfishes endemic to Laguna Chichancanab, Mexico. By comparing these patterns to our previous work on a recent radiation of *Cyprinodon* pupfishes in SSI, Bahamas (26, 28–32), we can examine the parallel and non-parallel features of these independent radiations in similar environments. Despite replicate radiations of Caribbean pupfishes in predator-free, large, isolated saline lakes with few competitors, we found little parallelism between the radiations. Although craniofacial morphology evolved rapidly within trophic specialists in both radiations, we found almost zero overlap in the adaptive genes underlying trophic specialization, potentially reflecting the divergent specialists within each radiation. There was no behavior-first stage of adaptive divergence in Chichancanab species and gene categories potentially relevant to speciation were not shared, such as pigmentation genes in SSI versus opsin and spermatogenesis genes in Chichancanab. Unlike the SSI radiation and most other well-studied adaptive radiations (e.g., Malawi cichlids (20), Galápagos finches (47)), adaptive introgression likely played a minimal role in the Chichancanab radiation. Instead, over 30% of the candidate adaptive variants in the zooplanktivore, *C. simus*, likely arose from de novo mutations compared to 2% in the SSI scale-eater specialist. The one parallel genetic feature was a clear final stage of refinement in the adaptive divergence of the zooplanktivore, marked by increased rates of de novo and nonsynonymous adaptive mutations occurring later in the radiation.

### Adaptive introgression did not contribute to adaptive radiation of Chichancanab pupfishes

Genomic analyses have shown that hybridization and introgression played a major role in many adaptive radiations (3, 3, 16, 19–27, 48, 49), including the *Cyprinodon* SSI radiation (26, 31). However, in the Chichancanab radiation, we find little evidence for introgression playing a major role, and did not detect any evidence of adaptive introgression following the colonization of Laguna Chichancanab. Although our analyses indicate extensive introgression among Cyprinodontidae lineages, we did not find any introgression events into the root of the radiation, nor has there been gene flow from species other than the sister-species to the radiation, *C. artifrons*. This secondary gene flow with *C. artifrons* is mostly between *C. beltrani* and, to a lesser extent, *C. labiosus.* Gene flow may be higher with these species because *C. artifrons,* a benthic feeding detritivore, is less ecologically divergent from both *C. beltrani,* a benthic feeding detritivore, and *C. labiosus*, a benthic feeding bivalve specialist. This secondary gene flow between *C. artifrons* and *C. beltrani* and *C. labiosus* overlaps with many genes under selection in *C. simus*, suggesting that secondary gene flow is potentially helping maintain divergence between *C. simus* and the benthic Chichancanab species not by bringing in new adaptive variation for trophic specialization, but by maintaining the generalist/omnivore species.

These results are remarkably different from the *Cyprinodon* adaptive radiation of SSI, where there is strong evidence of introgression at the root of the radiation, evidence of ongoing introgression, and the presence of many introgressed adaptive variants in both trophic specialists (26, 31). Laguna Chichancanab is a landlocked lake with no above-ground river connections and is geographically more isolated compared to SSI, a small island in the approximate center of the Caribbean. Furthermore, the sister species, *C. artifrons*, is abundant along the entire coast of the Yucatán, meaning that any gene flow into Chichancanab would likely first have to pass through *C. artifrons.* An additional, non-mutually exclusive explanation could be that the water chemistry of Laguna Chichancanab, with near-saturation of calcite and gypsum, is a greater barrier to non-adapted pupfish species than the hypersaline lakes of SSI. Thus, geography and ecology may play an important role in determining the relative role that introgression may play in radiations.

### De novo mutations play the dominant role in driving adaptive divergence of the zooplanktivore

The importance of de novo mutations in adaptive radiations remains contentious, in part due to the time needed for de novo beneficial mutations to arise (11, 15). Interestingly, we find that over 30% of the zooplanktivore adaptive variants were detected only within Chichancanab species, suggesting their de novo origins within the basin, approximately 10 times greater than the role of de novo mutations in the adaptive divergence of the scale-eater in the SSI radiation. A primary factor could be that the estimated effective population size for the Chichancanab radiation is approximately twice that of the SSI radiation (26), meaning that selection on de novo mutations would be more effective, with a higher probability of fixation, all else being equal (14). There may also be a lack of existing standing genetic variation relevant to the trophic specialization of zooplanktivory. Although radiations should proceed along the genetic lines of least resistance (50), the morphological axis along which the zooplanktivore has diverged is orthogonal to almost all morphological variation that exists in Caribbean pupfish species (29). Evolving along the line of greatest resistance instead of the line of least resistance may require de novo mutations. While the SSI scale-eater is also highly divergent, it is closer to the existing morphological space of Caribbean pupfish species and in the direction of greatest variance, at least within the two phenotypic dimensions of greatest variance (29). Finally, an alternative possibility could be that many of these de novo mutations do exist as standing variation and were missed in our dataset, but this is unlikely to explain all de novo mutations, given our extensive sampling of *Cyprinodon* species across the clade and spanning the Caribbean. Thus, our results highlight that de novo mutations may play a more important role in driving young adaptive radiations than currently appreciated (12, 13), especially when ecotypes are highly divergent from other species within the radiation.

### Extremely limited overlap in the genes underlying adaptive radiations of trophic specialists

We found only two candidate genes (*DNM1* and *SPTLC3*) among the 55 zooplanktivore candidate genes in the Chichancanab radiation that overlapped with the hundreds of candidate genes identified so far in the SSI radiation (26, 28, 30, 32, 51–55). Consistent with both trophic specializations requiring the pursuit of mobile prey, both genes are involved in motor neuron development. *DNM1* is a dynamin with nervous tissue-specific expression that is crucial for the proper formation of motor neuron axons (41). *SPTLC3* is involved in the biosynthesis of sphingolipids (56), and suppression of *SPTLC3* in zebrafish results in motor neuron axon defects (42).

The limited number of shared adaptive genes is somewhat surprising given the high conservation of gene function in both morphological development (57, 58) and behavior (59), and the frequent reuse of the same candidate genes among vertebrate adaptive radiations, particularly for craniofacial genes. For example, *BMP4* is involved in diversification in both Galápagos finches (60) and African cichlids (61, 62). Further, within the SSI radiation, many of the same genes are involved in craniofacial divergence between divergent trophic specialists (26, 53). Selection may still be acting on the same genetic pathways and genetic regulatory networks missed by our direct candidate comparisons.

Another potential reason for this non-parallelism could be that trophic specialists in each radiation have diverged in different sets of traits (29). Specifically, the Chichancanab radiation has diversified in tooth length over 130 times faster than background rates, driven by the extremely short teeth of the zooplanktivore (29). Consistent with divergence in tooth morphology, one of the candidate craniofacial genes with many de novo mutations in flanking and intronic regions was *WNT10A*. Mutations in *WNT10A* are associated with tooth developmental disorders in humans (63). Furthermore, knockout and overexpression experiments in sticklebacks and zebrafish have demonstrated *WNT10A*’s role in teeth development and regeneration (64, 65).

### The parallel role of vision but the non-parallelism of adaptive candidate genes

One of the more striking features of both *Cyprinodon* radiations is the evolution of novel trophic specialists from a generalist ancestor: the scale-eater (*C. desquamator*) in the SSI radiation and the zooplanktivore (*C. simus*) in the Chichancanab radiation. Although these are distinct dietary specializations, they share a similarity in that they require tracking free-swimming prey. Indeed, four of the top 15 enriched GO terms of candidate genes in the SSI scale-eater were related to eye development (26). Similarly, several of the candidate genes we identified in the zooplanktivore were related to eye development (*ASIC3*, *MAB21L2*, and *CRIM1*). In zebrafish, a two-basepair deletion of *CRIM1* results in misshapen lenses as well as a large eye-to-head ratio (66). Interestingly, one of the defining morphological features of *C. simus* is its large eye-to-head ratio (33) (Figure 1A). *CRIM1* is also a promising candidate because it is one of the few candidate genes with multiple nonsynonymous mutations (Figure 2B).

Consistent with candidate genes related to eye development and trophic specialization, *C. simus* does indeed exhibit a stronger optomotor response compared to *C. beltrani*, indicating that this specialist has greatly improved visual acuity to detect rapidly rotating black-and-white bars.

Although both radiations contained many vision-related candidate genes, there was zero overlap in these genes. Most notably, we found several adaptive variants associated with opsins (*SWS2*, blue-sensitive, and *OPN1LW*, red-sensitive) in the zooplanktivore. However, in the SSI radiation, opsins do not appear to be under selection in any species (26). This result is somewhat surprising given that opsins appear to be evolutionarily labile and rapidly evolve in other fish lineages (e.g., sticklebacks (67), cichlids (68, 69), wrasses (70), reviewed in (71)). One plausible explanation could be that SSI lake environments are relatively shallow, so differences in depth—a common selective pressure on opsin evolution (71)—are likely not a strong selective force on SSI. Alternatively, the main selective force acting on opsins may be prey capture efficiency. In many fish species, zooplanktivory is associated with higher expression of shorter wavelength opsins (e.g., *SWS1* (71)), whereas piscivory is not often associated with changes in opsin expression (68, 69). Interestingly, both opsins contain many candidate de novo mutations, and *SWS2* is the youngest estimated sweep in the zooplanktivore. One explanation could be that most Caribbean *Cyprinodon* species are benthic and live in similar habitats, resulting in limited standing genetic variation in opsins.

### Non-parallelism of sexual selection: the relative role of pre- and post-mating sexual selection

Although the role that premating sexual selection plays in speciation has historically been the main focus (72, 73), there is both theoretical and empirical support for the role of postmating sexual selection in maintaining reproductive isolation via postmating prezygotic reproductive barriers (74–79). In the SSI radiation, species differ in pigmentation, and a handful of the candidate adaptive variants fall in regulatory regions of genes associated with pigmentation (26). Furthermore, there is evidence for premating isolation by female preference in the scale-eater (80). However, in Chichancanab, *C. labiosus* does not visually distinguish between different species in the radiation (81). Unlike the SSI radiation, we did not find any candidate adaptive variants associated with pigmentation; instead, we identified adaptive variants associated with spermatogenesis (*ERC2*, *TBC1D25*, *C4orf22*, *STK35*). Consistent with previous work demonstrating the importance of these genes in spermatogenesis (82–85), we find that *C. simus* does indeed have larger sperm heads than *C. beltrani*, suggesting a potential role for post-mating prezygotic reproductive barriers.

### Parallelism in the final stages of adaptation: refinement with de novo and nonsynonymous mutations

Adaptation may occur in stages, with less pleiotropic cis-regulatory changes occurring before more pleiotropic coding changes (26, 86, 87). Selection is also predicted to act on standing variation first due to the waiting time for de novo mutations to arise within a population (14, 88). In both radiations (Richards et al., 2021), we observe strong evidence for these stages of adaptation.

The simplest explanation for why we see parallelism in de novo mutations sweeping later could be due to the waiting time required for a beneficial mutation to (1) arise and (2) reach a substantial frequency within the population (11, 14). Although de novo and nonsynonymous mutations were not statistically associated across all candidate variants, many of the late sweeping adaptive variants are both de novo and nonsynonymous. These variants may also sweep late because nonsynonymous mutations are often pleiotropic and deleterious in an ancestral genetic background (14, 89). Thus, they may not be advantageous until later in the adaptive walk, where compensatory epistasis may mediate negative pleiotropic effects (90–94). Our results highlight that this final refinement stage is likely a general pattern to be expected across adaptive radiations.

## Methods

### Sampling

We collected Laguna Chichancanab specimens in 2022 over two days using a 5 × 1.3 m seine net with 1.6 mm mesh size. We sampled two sites, an entrance road by La Presumida, and a bridge crossing the narrow section of the lake on the road to San Diego. *C. labiosus* and *C. beltrani* were sampled from both sites for this study, whereas *C. simus* was only collected at La Presumida. *C. artifrons* were collected by cast net from the coastal estuary at Sisal. Additional specimens were collected from the Bahamas (Lake Cunningham, New Providence Island and Crescent Pond, San Salvador Island), the Dominican Republic (Laguna Bavaro), or Fort Fisher, North Carolina as described previously (Martin 2016) or sourced from the American Killifish Association, the London Zoological Society, the Dallas Children’s Aquarium, and Frans Vermuelen. All specimens were euthanized in an overdose of buffered MS-222 (Fiquel, Inc.) following approved animal care and use protocols from the University of California, Berkeley and the University of California, Davis.

### Sequencing, Variant calling and filtering

Individual DNA samples were resequenced using Illumina Hiseq 4000 and Novaseq. Raw reads were trimmed with *fastp* (v0.23.4) (95). Reads were mapped to the UCB_CyNevMio_1.0 *Cyprindon nevadensis mionectes* reference genome (GCA_030533445.1) with *bwa mem* (v0.7.17) (96). Duplicate reads in the bam files were marked with *Picard MarkDuplicates* (GATK v4.5.0.0) (118). Coverage and mapping quality were assessed with *qualimap* (v2.2.2) (97). We followed the *GATK* (v.4.5.0.0) genotyping pipeline to call variants (119). We used *HaplotypeCaller* (-ERC GVCF) v4.5.0.0 to call variants for each individual and stored variants in the GenomicsDB datastore format prior to using *GenotypeGVCFs* to perform joint genotyping. We restricted our analyses to biallelic SNPs and applied the recommended *GATK* hard filters (QD < 2, QUAL < 30, SOR > 3, FS > 60, MQ < 40, MQRankSum < −12.5, ReadPosRankSum < −8) to filter SNPs (118, 120). Missing genotypes were reset to ./. from 0/0 due to how *GATK* represents missing genotypes in version 4.5.0.0 (101).

After hard-filtering and variant calling, we filtered the data set further by removing SNPs with minor allele frequency < 0.05, more than 10% missing, depth less than 10x, and GQ less than 20 using *BCFtools* (v1.16)(102). This resulted in a final data set that included 23,960,536 SNPs.

### Population structure of Yucatán Cyprinodon pupfishes

To assess population structure, we first pruned SNPs in linkage disequilibrium using *PLINK* (v. 1.9) with the following parameters: “-indep-pairwise 50 5 0.2” (103). This filtered our data set from 23,960,536 SNPs to 5,476,855 SNPs. With this linkage disequilibrium pruned data set, we first filtered the full VCF file to only include relevant Yucatán *Cyprinodon* species (i.e., *C. artifrons, C. beltrani, C. labiosus,* and *C. simus*), then conducted a PCA using *PLINK*. Next, we used *ADMIXTURE* (v1.3) to determine the optimal number of population clusters that best fit the data and to assign individuals to their corresponding population clusters (104). We performed this analysis and calculated cross-validation error for the number of clusters K = 1–6. Models in ADMIXTURE with K=2–4 were equally supported (Figure S2A). However, given morphological distinctness and the results of the PCA, we present the results for when K = 4 (Figure 1D).

### Demographic history of Yucatán Cyprinodon pupfish

We estimated demographic history using *MSMC2* (105). For each Yucatán pupfish species, we selected three individuals with the highest mean depth and generated single-sample VCF files, individual mask files, and “mapability” mask files. We then ran *MSMC2* with default parameters except we set phasing to “unphased” and “P_PAR=8*1+25*1+1*2+1*3.” For plotting, we used a generation time of 1 year and a mutation rate of 1.56 × 10^−8^ substitutions per base pair, estimated from Caribbean *Cyprinodon* species (26).

### Identifying candidate genes

We calculated *F*_*st*_ genome-wide, per site, and in non-overlapping 10-kb windows using the “--weir-fist-pop” function in *VCFtools*(v0.1.16) for all pairwise combinations of Yucatán *Cyprinodon* species (106). We chose 10-kb windows because linkage disequilibrium was substantially decayed at this distance (Figure S3), and these windows allowed us to quantify finer-scale genomic variation. With the 10-kb windowed *F*_*st*_ values, we calculated a modified population branch statistic (PBS) for four taxa, *PBS*_*nj*_ (107). This statistic is useful because it makes no assumptions about species topology or polarization (107). We then normalized *PBS*_*nj*_ by the total tree length, as normalized *PBS* is most effective and specific at identifying selective sweeps (40). We classified SNPs as candidates if they were fixed or nearly fixed (per-site *F*_*st*_ >0.95) compared to other species within the radiation and were among the top 1% outliers out of all *PBS*_*nj*_ windows, following the threshold used in (40).

### Introgression

Using the full VCF file, which contained a wide range of species in the family Cyprinodontidae, we tested for evidence of introgression in Yucatán *Cyprinodon* species using *Dsuite* (43). We first excluded any samples/species that had an average depth < 4. We then calculated all possible trios with a species tree based on the most recent phylogeny (108) with *Cualac tessellatus* as the outgroup. We calculated Z-scores to assess the significance of *D* statistics using 1,000 jackknife blocks and considered a Z-score greater than three as evidence for significant introgression (109). We only focused our primary analysis on species trios where P1-P2 included species within the Laguna Chichancanab radiation. We used the other trio calculations to calculate *f*-branch (*f*_*b*_) statistics to account for correlated *f*_*4*_-ratio scores and to inform the timing of introgression events (43).

For species trios that showed evidence of introgression where *C. simus* was either P1or P2, we then calculated the distance fraction (*d*_*f*_), a robust metric of introgression for windowed analyses (44), in non-overlapping windows of 91 informative SNPs. We chose this number of SNPs because it resulted in windows of approximately 10 kb. We considered windows to be introgressed if they were in the top X% of absolute *d*_*f*_ value, where X% was the percentage of estimated genome wide introgression (e.g, *f*_*4*_-ratio) (110, 111). To estimate introgression block sizes, we merged neighboring windows that were both introgression outliers and took the total size of the blocks.

### Characterizing candidate genes

After identifying candidate SNPs, we then used *Bedtools* (v2.31) to see if SNPs occurred near (20-kb) or within a gene (112) with a GFF annotation file for the *C. nev. mionectes* reference genome (Tian et al. in prep). We classified SNPs within 20 kb of a gene (upstream or downstream) as flanking. For SNPs within a gene, we further classified them into intronic, synonymous, or nonsynonymous using *SnpEff* (113).

To characterize the origin of candidate SNPs, we extracted the alleles from every species for each candidate SNP. We classified variants that were found in any other species as “standing variation”. We further subdivided this group into variants that were found in other species but not in *C. artifrons*. If “standing variation” variants fell within an introgression window, we classified them as “introgressed.” Finally, if the alternative variant was only found in Chichancanab species, we classified it as de novo.

### Timing of selective sweeps

To gain insight into the timing of selective sweeps in *C. simus*, we used *starTRMCA* (45). Because many genes contained multiple candidate SNPs, for this analysis, we selected the variant that was in the median position of each gene. We then extracted a 1-Mb region surrounding that variant and removed all sites that contained missing data. For simplification, we only used the generalist/detritivore, *C. beltrani*, as the “reference” population and the zooplanktivore, *C. simus*, as the “selected” population. We used a fixed recombination rate of 2×10^−8^ (swordtail fishes; (114)) and a mutation rate of 1.56×10^−8^ substitutions per base pair (Caribbean *Cyprinodon* species; 26). We then ran five separate Markov chains for 30,000 iterations with a proposal standard deviation of 150 (preliminary analyses changing this parameter had minimal influence on results). We then discarded the first 10,000 iterations of each chain as burn-in.

To test for stages of adaptation, we conducted separate generalized linear regressions to test if the sweep age was correlated with the proportion of nonsynonymous SNPs, proportion of de novo SNPs, and the number of SNPs. For the generalized linear regressions of proportion of nonsynonymous SNPs and proportion of de novo SNPs, we used a quasi-binomial family to account for overdispersion with a logit link function. For the number of SNPs, we used a quasi-Poisson family with a log link function. To test for the significance of sweep age, we used a likelihood ratio test comparing a model with the estimated sweep age as an effect compared to a null model.

### Optomotor response

We tested for differences in visual acuity between the generalist/detritivore, *C. beltrani*, and the zooplanktivore, *C. simus*, by conducting an optomotor response behavioral assay. We tested lab-reared *C. beltrani* (F1, *n* = 7) and lab-reared *C. simus* (long-term laboratory colony exceeding ten generations in the lab, *n* = 5). Each fish was tested consecutively with each control or treatment for 1-minute observation periods, with the control observation occurring first, followed by presentation of all-black and black-and-white spinning bars. The order of presentation for the latter to treatments was alternated in each trial. Fish were placed in a suspended cylindrical plastic container 0.3 m in diameter that remained stationary during the course of the trials. Spinning black-and-white or all-black paper was rotated outside the clear plastic container at an RPM of ~90rpm during each observation period.

Due to the data being nonparametric (count, zero-inflated, unequal variance between groups), we conducted a Wilcoxon rank sum test for the on-banded (all black) and on-banded portions separately. Because we had an a priori hypothesis that *C. simus* would display a stronger optomotor response (greater visual acuity), we calculated *p*-values with a one-tailed test.

### Sperm morphology

To test for differences in sperm morphology, we collected sperm samples from five individuals per species in the lab (*C. simus, C. artifrons, C. beltrani*). We first anesthetized the fish with a solution of MS-222. After the fish was anesthetized, we collected 1μL of milt (fish semen) with a 1μL glass microcapillary tube and fixed the sample overnight in a 4% PFA solution stained with Rose Bengal. We then plated sperm on a positively charged slide with a sealed coverslip. We imaged sperm with oil immersion with a 63x objective and measured 10–30 sperm per individual in ImageJ (115). Due to poor sperm samples from some individuals (little sperm), we ended up with the following sample sizes: three *simus*, four *artifrons*, and five *beltrani*.

To retain information about variation within individuals, we fit separate linear mixed-effects models for each trait of interest (sperm head, midpiece, and flagellum lengths) with population as a fixed effect and a random intercept for individual ID, using *LME4* (116). To assess the significance of the model, we used a Type II ANOVA with a Wald *F* test using Kenward-Roger degrees of freedom. If there was a significant effect of population, we conducted post hoc pairwise tests using the *Emmeans* package (117) and corrected for multiple comparisons using the Tukey method. We did not correct for phylogeny due to how closely related the species are, the limited sample size, and the fact that the only significant differences were between sister species.

## Supplementary Material

Supplement 1

## References

[1] Gillespie R. G., , Comparing Adaptive Radiations Across Space, Time, and Taxa. J Hered. 111, 1–20 (2020). 31958131 10.1093/jhered/esz064 PMC7931853

[2] Martin C. H., Richards E. J., The Paradox Behind the Pattern of Rapid Adaptive Radiation: How Can the Speciation Process Sustain Itself Through an Early Burst? Annu. Rev. Ecol. Evol. Syst. 50, 569–593 (2019). 36237480 10.1146/annurev-ecolsys-110617-062443 PMC9555815

[3] Naciri Y., Linder H. P., The genetics of evolutionary radiations. Biol. Rev. 95, 1055–1072 (2020). 32233014 10.1111/brv.12598

[4] Schluter D., The Ecology of Adaptive Radiation (OUP Oxford, 2000).

[5] Stroud J. T., Losos J. B., Ecological Opportunity and Adaptive Radiation. Annu. Rev. Ecol. Evol. Syst. 47, 507–532 (2016).

[6] Butlin R., , What do we need to know about speciation? Trends Ecol. Evol. 27, 27–39 (2012). 21978464 10.1016/j.tree.2011.09.002

[7] Coyne J. A., Orr H. A., Speciation (Oxford University Press, 2004).

[8] Matute D. R., Cooper B. S., Comparative studies on speciation: 30 years since Coyne and Orr. Evolution 75, 764–778 (2021). 33491225 10.1111/evo.14181 PMC8247902

[9] Schluter D., Rieseberg L. H., Three problems in the genetics of speciation by selection. Proc. Natl. Acad. Sci. U.S.A. 119, e2122153119 (2022). 35858397 10.1073/pnas.2122153119 PMC9335311

[10] Berner D., Salzburger W., The genomics of organismal diversification illuminated by adaptive radiations. Trends Genet. 31, 491–499 (2015). 26259669 10.1016/j.tig.2015.07.002

[11] Marques D. A., Meier J. I., Seehausen O., A Combinatorial View on Speciation and Adaptive Radiation. Trends Ecol. Evol. 34, 531–544 (2019). 30885412 10.1016/j.tree.2019.02.008

[12] Pease J. B., Haak D. C., Hahn M. W., Moyle L. C., Phylogenomics Reveals Three Sources of Adaptive Variation during a Rapid Radiation. PLOS Biol. 14, e1002379 (2016). 26871574 10.1371/journal.pbio.1002379 PMC4752443

[13] Stone B. W., Wessinger C. A., Ecological Diversification in an Adaptive Radiation of Plants: The Role of De Novo Mutation and Introgression. Mol. Biol. Evol. 41, msae007 (2024). 38232726 10.1093/molbev/msae007 PMC10826641

[14] Barrett R. D. H., Schluter D., Adaptation from standing genetic variation. Trends Ecol. Evol. 23, 38–44 (2008). 18006185 10.1016/j.tree.2007.09.008

[15] Rousselle M., , Is adaptation limited by mutation? A timescale-dependent effect of genetic diversity on the adaptive substitution rate in animals. PLOS Genet. 16, e1008668 (2020). 32251427 10.1371/journal.pgen.1008668 PMC7162527

[16] Seehausen O., Hybridization and adaptive radiation. Trends Ecol. Evol. 19, 198–207 (2004). 16701254 10.1016/j.tree.2004.01.003

[17] Kagawa K., Takimoto G., Seehausen O., Transgressive segregation in mating traits drives hybrid speciation. Evolution 77, 1622–1633 (2023). 37094817 10.1093/evolut/qpad072

[18] Kagawa K., Takimoto G., Hybridization can promote adaptive radiation by means of transgressive segregation. Ecol. Lett. 21, 264–274 (2018). 29243294 10.1111/ele.12891

[19] De-Kayne R., , Genomic architecture of adaptive radiation and hybridization in Alpine whitefish. Nat. Commun. 13, 4479 (2022). 35918341 10.1038/s41467-022-32181-8 PMC9345977

[20] Joyce D. A., , Repeated colonization and hybridization in Lake Malawi cichlids. Curr. Biol. 21, R108–R109 (2011). 21300271 10.1016/j.cub.2010.11.029

[21] Lamichhaney S., , Evolution of Darwin’s finches and their beaks revealed by genome sequencing. Nature 518, 371–375 (2015). 25686609 10.1038/nature14181

[22] Martin C. H., , Complex histories of repeated gene flow in Cameroon crater lake cichlids cast doubt on one of the clearest examples of sympatric speciation. Evolution 69, 1406–1422 (2015). 25929355 10.1111/evo.12674

[23] Meier J. I., , Ancient hybridization fuels rapid cichlid fish adaptive radiations. Nat. Commun. 8, 14363 (2017). 28186104 10.1038/ncomms14363 PMC5309898

[24] Pouchon C., , Phylogenomic Analysis of the Explosive Adaptive Radiation of the Espeletia Complex (Asteraceae) in the Tropical Andes. Syst. Biol. 67, 1041–1060 (2018). 30339252 10.1093/sysbio/syy022

[25] Richards E. J., Poelstra J. W., Martin C. H., Don’t throw out the sympatric speciation with the crater lake water: fine-scale investigation of introgression provides equivocal support for causal role of secondary gene flow in one of the clearest examples of sympatric speciation. Evol. Lett. 2, 524–540 (2018). 30283699 10.1002/evl3.78 PMC6145409

[26] Richards E. J., , A vertebrate adaptive radiation is assembled from an ancient and disjunct spatiotemporal landscape. Proc. Natl. Acad. Sci. U.S.A. 118, e2011811118 (2021). 33990463 10.1073/pnas.2011811118 PMC8157919

[27] Rosser N., , Hybrid speciation driven by multilocus introgression of ecological traits. Nature 628, 811–817 (2024). 38632397 10.1038/s41586-024-07263-w PMC11041799

[28] John M. E. S., Dunker J. C., Richards E. J., Romero S., Martin C. H., Parallel evolution of integrated craniofacial traits in trophic specialist pupfishes. Ecol. Evol. 14, e11640 (2024). 38979003 10.1002/ece3.11640 PMC11228360

[29] Martin C. H., Wainwright P. C., Trophic novelty is linked to exceptional rates of morphological diversification in two adaptive radiations of cyprinodon pupfish. Evolution 65, 2197–2212 (2011). 21790569 10.1111/j.1558-5646.2011.01294.x

[30] Patton A. H., Richards E. J., Gould K. J., Buie L. K., Martin C. H., Hybridization alters the shape of the genotypic fitness landscape, increasing access to novel fitness peaks during adaptive radiation. eLife 11, e72905 (2022). 35616528 10.7554/eLife.72905 PMC9135402

[31] Richards E. J., Martin C. H., Adaptive introgression from distant Caribbean islands contributed to the diversification of a microendemic adaptive radiation of trophic specialist pupfishes. PLOS Genet. 13, e1006919 (2017). 28796803 10.1371/journal.pgen.1006919 PMC5552031

[32] Richards E. J., Martin C. H., We get by with a little help from our friends: shared adaptive variation provides a bridge to novel ecological specialists during adaptive radiation. Proc. R. Soc. B. 289, 20220613 (2022). 10.1098/rspb.2022.0613 PMC9130792 35611537

[33] Humphries J. M., Miller R. R., A Remarkable Species Flock of Pupfishes, Genus Cyprinodon, from Yucatán, México. Copeia 1981, 52–64 (1981).

[34] Hodell D. A., Curtis J. H., Brenner M., Possible role of climate in the collapse of Classic Maya civilization. Nature 375, 391–394 (1995).

[35] Strecker U., Description of a new species from Laguna Chichancanab, Yucatan, Mexico: Cyprinodon suavium (Pisces: Cyprinodontidae). Hydrobiologia 541, 107–115 (2005).

[36] Gracida-Juárez C. A., Schmitter-Soto J. J., Genner M. J., Community structure of indigenous fishes relative to habitat variation and invasive tilapia in lakes of Quintana Roo, Mexico. Environ. Biol. Fishes 107, 401–414 (2024).

[37] Strecker U., The impact of invasive fish on an endemic Cyprinodon species flock (Teleostei) from Laguna Chichancanab, Yucatan, Mexico. Ecol. Freshw. Fish 15, 408–418 (2006).

[38] Strecker U., Genetic differentiation and reproductive isolation in a Cyprinodon fish species flock from Laguna Chichancanab, Mexico. Mol. Phylogenetics Evol. 39, 865–872 (2006). 10.1016/j.ympev.2006.01.008 16483800

[39] Strecker U., Meyer C. G., Sturmbauer C., Wilkens H., Genetic Divergence and Speciation in an Extremely Young Species Flock in Mexico Formed by the GenusCyprinodon(Cyprinodontidae, Teleostei). Mol. Phylogenetics Evol. 6, 143–149 (1996). 10.1006/mpev.1996.0066 8812314

[40] Shpak M., Lawrence K. N., Pool J. E., The Precision and Power of Population Branch Statistics in Identifying the Genomic Signatures of Local Adaptation. Genome Biol. Evol. 17, evaf080 (2025). 40326284 10.1093/gbe/evaf080 PMC12095133

[41] Bragato C., , Zebrafish dnm1a gene plays a role in the formation of axons and synapses in the nervous tissue. J. Neurosci. Res. 101, 1345–1359 (2023). 37031448 10.1002/jnr.25197

[42] Gonzaga-Jauregui C., , Exome Sequence Analysis Suggests that Genetic Burden Contributes to Phenotypic Variability and Complex Neuropathy. Cell Reports 12, 1169–1183 (2015). 26257172 10.1016/j.celrep.2015.07.023 PMC4545408

[43] Malinsky M., Matschiner M., Svardal H., Dsuite - Fast D-statistics and related admixture evidence from VCF files. Mol. Ecol. Res. 21, 584–595 (2021). 10.1111/1755-0998.13265 PMC7116594 33012121

[44] Pfeifer B., Kapan D. D., Estimates of introgression as a function of pairwise distances. BMC Bioinform. 20, 207 (2019). 10.1186/s12859-019-2747-z PMC6480520 31014244

[45] Smith J., Coop G., Stephens M., Novembre J., Estimating Time to the Common Ancestor for a Beneficial Allele. Mol. Biol. Evol. 35, 1003–1017 (2018). 29361025 10.1093/molbev/msy006 PMC5888984

[46] Neuhauss S. C. F., , Genetic Disorders of Vision Revealed by a Behavioral Screen of 400 Essential Loci in Zebrafish. J. Neurosci. 19, 8603–8615 (1999). 10493760 10.1523/JNEUROSCI.19-19-08603.1999 PMC6783047

[47] Enbody E. D., , Community-wide genome sequencing reveals 30 years of Darwin’s finch evolution. Science 381, eadf6218 (2023). 37769091 10.1126/science.adf6218

[48] Peñalba J. V., , The Role of Hybridization in Species Formation and Persistence. Cold Spring Harb. Perspect. Biol. 16, a041445 (2024). 38438186 10.1101/cshperspect.a041445 PMC11610762

[49] Seehausen O., Conditions when hybridization might predispose populations for adaptive radiation. J. Evol. Biol. 26, 279–281 (2013). 23324007 10.1111/jeb.12026

[50] Schluter D., Adaptive radiation along genetic lines of least resistance. Evolution 50, 1766–1774 (1996). 28565589 10.1111/j.1558-5646.1996.tb03563.x

[51] McGirr J. A., Martin C. H., Few Fixed Variants between Trophic Specialist Pupfish Species Reveal Candidate Cis-Regulatory Alleles Underlying Rapid Craniofacial Divergence. Mol. Biol. Evol. 38, 405–423 (2021). 32877534 10.1093/molbev/msaa218 PMC7826174

[52] McGirr J. A., Martin C. H., Novel Candidate Genes Underlying Extreme Trophic Specialization in Caribbean Pupfishes. Mol. Biol. Evol.34, 873 (2017). 28028132 10.1093/molbev/msw286 PMC5850223

[53] McGirr J. A., Martin C. H., Parallel evolution of gene expression between trophic specialists despite divergent genotypes and morphologies. Evol. Lett. 2, 62–75 (2018). 30283665 10.1002/evl3.41 PMC6089502

[54] McGirr J. A., Martin C. H., Ecological divergence in sympatry causes gene misexpression in hybrids. Mol. Ecol. 29, 2707–2721 (2020). 32557903 10.1111/mec.15512 PMC8209238

[55] Palominos M. F., Muhl V., Martin C. H., Craniofacial-specific transcriptomics uncovers novel genes underlying jaw divergence in dietary specialist pupfishes. Genetics iyaf207 (2025). 41001823 10.1093/genetics/iyaf207 PMC12693500

[56] Hornemann T., , The SPTLC3 Subunit of Serine Palmitoyltransferase Generates Short Chain Sphingoid Bases. J. of Biol. Chem. 284, 26322–26330 (2009). 19648650 10.1074/jbc.M109.023192 PMC2785320

[57] Bronner M. E., LeDouarin N. M., Development and evolution of the neural crest: An overview. Dev. Biol. 366, 2–9 (2012). 22230617 10.1016/j.ydbio.2011.12.042 PMC3351559

[58] Hirth F., Reichert H., Conserved genetic programs in insect and mammalian brain development. BioEssays 21, 677–684 (1999). 10440864 10.1002/(SICI)1521-1878(199908)21:8<677::AID-BIES7>3.0.CO;2-8

[59] Reaume C. J., Sokolowski M. B., Conservation of gene function in behaviour. Phil. Tran. R. Soc. B. 366, 2100–2110 (2011). 10.1098/rstb.2011.0028 PMC3130371 21690128

[60] Abzhanov A., Protas M., Grant B. R., Grant P. R., Tabin C. J., Bmp4 and Morphological Variation of Beaks in Darwin’s Finches. Science 305, 1462–1465 (2004). 15353802 10.1126/science.1098095

[61] Albertson R. C., Streelman J. T., Kocher T. D., Yelick P. C., Integration and evolution of the cichlid mandible: The molecular basis of alternate feeding strategies. Proc. Nat. Acad. Sci. U.S.A. 102, 16287–16292 (2005). 10.1073/pnas.0506649102 PMC1283439 16251275

[62] Terai Y., Morikawa N., Okada N., The Evolution of the Pro-Domain of Bone Morphogenetic Protein 4 (Bmp4) in an Explosively Speciated Lineage of East African Cichlid Fishes. Mol. Biol. Evol. 19, 1628–1632 (2002). 12200490 10.1093/oxfordjournals.molbev.a004225

[63] Nawaz S., , WNT10A missense mutation associated with a complete Odonto-Onycho-Dermal Dysplasia syndrome. Eur. J. Hum. Genet. 17, 1600–1605 (2009). 19471313 10.1038/ejhg.2009.81 PMC2987016

[64] Benard E. L., , wnt10a is required for zebrafish median fin fold maintenance and adult unpaired fin metamorphosis. Dev. Dyn. 253, 566–592 (2024). 37870737 10.1002/dvdy.672 PMC11035493

[65] Square T. A., , Modulation of tooth regeneration through opposing responses to Wnt and BMP signals in teleosts. Development 150, dev202168 (2023). 38059590 10.1242/dev.202168 PMC10730089

[66] Le T., , A zebrafish model of crim1 loss of function has small and misshapen lenses with dysregulated clic4 and fgf1b expression. Front. Cell Dev. Biol. 13 (2025). 10.3389/fcell.2025.1522094 PMC11922885 40114969

[67] Marques D. A., , Convergent evolution of SWS2 opsin facilitates adaptive radiation of threespine stickleback into different light environments. PLOS Biol.15, e2001627 (2017). 28399148 10.1371/journal.pbio.2001627 PMC5388470

[68] Hofmann C. M., , The Eyes Have It: Regulatory and Structural Changes Both Underlie Cichlid Visual Pigment Diversity. PLOS Biol.7, e1000266 (2009). 20027211 10.1371/journal.pbio.1000266 PMC2790343

[69] O’Quin K. E., Hofmann C. M., Hofmann H. A., Carleton K. L., Parallel Evolution of Opsin Gene Expression in African Cichlid Fishes. Mol. Biol. Evol. 27, 2839–2854 (2010). 20601410 10.1093/molbev/msq171

[70] Phillips G. A. C., Carleton K. L., Marshall N. J., Multiple Genetic Mechanisms Contribute to Visual Sensitivity Variation in the Labridae. Mol. Biol. Evol. 33, 201–215 (2016). 26464127 10.1093/molbev/msv213 PMC5009993

[71] Carleton K. L., Escobar-Camacho D., Stieb S. M., Cortesi F., Marshall N. J., Seeing the rainbow: mechanisms underlying spectral sensitivity in teleost fishes. J. Exp. Biol. 223, jeb193334 (2020). 32327561 10.1242/jeb.193334 PMC7188444

[72] Ritchie M. G., Sexual Selection and Speciation. Annu. Rev. Ecol. Evol. Syst.38, 79–102 (2007).

[73] Servedio M. R., Boughman J. W., The Role of Sexual Selection in Local Adaptation and Speciation. Annu. Rev. Ecol. Evol. Syst. 48, 85–109 (2017).

[74] Devigili A., , Possible glimpses into early speciation: the effect of ovarian fluid on sperm velocity accords with post-copulatory isolation between two guppy populations. J. Evol. Biol.31, 66–74 (2018). 29044818 10.1111/jeb.13194

[75] Garlovsky M. D., , Synthesis and Scope of the Role of Postmating Prezygotic Isolation in Speciation. Cold Spring Harb. Perspect. Biol. a041429 (2023). 10.1101/cshperspect.a041429 PMC11444258 38151330

[76] Kustra M. C., Servedio M. R., Alonzo S. H., Cryptic female choice can maintain reproductive isolation. Evolution 79, 2259–2273 (2025). 40728922 10.1093/evolut/qpaf156

[77] Kustra M. C., Alonzo S. H., The coevolutionary dynamics of cryptic female choice. Evol. Lett. 7, 191–202 (2023). 37475752 10.1093/evlett/qrad025 PMC10355280

[78] Lorch P. D., Servedio M. R., The evolution of conspecific gamete precedence and its effect on reinforcement. J. Evol. Biol. 20, 937–949 (2007). 17465905 10.1111/j.1420-9101.2007.01306.x

[79] Marshall J. L., Arnold M. L., Howard D. J., Reinforcement: the road not taken. Trends Ecol. Evol. 17, 558–563 (2002).

[80] Kodric-Brown A., West R. J. D., Asymmetries in premating isolating mechanisms in a sympatric species flock of pupfish (Cyprinodon). Behav. Ecol. 25, 69–75 (2014).

[81] Strecker U., Kodric-Brown A., Mate recognition systems in a species flock of Mexican pupfish. J. Evol. Biol. 12, 927–935 (1999).

[82] Chau M. H. K., , Investigation of the genetic etiology in male infertility with apparently balanced chromosomal structural rearrangements by genome sequencing. Asian J. Androl. 24 (2022). 10.4103/aja2021106 PMC9226698 35017386

[83] Li H., Dai Y., Luo Z., Nie D., Cloning of a new testis-enriched gene C4orf22 and its role in cell cycle and apoptosis in mouse spermatogenic cells. Mol. Biol. Rep. 46, 2029–2038 (2019). 30820741 10.1007/s11033-019-04651-8

[84] Miyamoto Y., , The STK35 locus contributes to normal gametogenesis and encodes a lncRNA responsive to oxidative stress. Biol. Open 7, bio032631 (2018). 29970477 10.1242/bio.032631 PMC6124569

[85] Nawaz S., Hussain S., Basit S., Ahmad W., First evidence of involvement of *TBC1D25* in causing human male infertility. Eur. J. Med. Genet. 64, 104142 (2021). 33460826 10.1016/j.ejmg.2021.104142

[86] Campagna L., , Repeated divergent selection on pigmentation genes in a rapid finch radiation. Sci. Adv. 3, e1602404 (2017). 28560331 10.1126/sciadv.1602404 PMC5443641

[87] Jones F. C., , The genomic basis of adaptive evolution in threespine sticklebacks. Nature 484, 55–61 (2012). 22481358 10.1038/nature10944 PMC3322419

[88] Blount Z. D., Lenski R. E., Losos J. B., Contingency and determinism in evolution: Replaying life’s tape. Science 362, eaam5979 (2018). 30409860 10.1126/science.aam5979

[89] Hedrick P. W., Adaptive introgression in animals: examples and comparison to new mutation and standing variation as sources of adaptive variation. Mol. Ecol. 22, 4606–4618 (2013). 23906376 10.1111/mec.12415

[90] Das S. G., Mungan M., Krug J., Epistasis-mediated compensatory evolution in a fitness landscape with adaptational tradeoffs. Proc. Natl. Acad. Sci. U.S.A. 122, e2422520122 (2025). 40215274 10.1073/pnas.2422520122 PMC12012525

[91] Harms M. J., Thornton J. W., Historical contingency and its biophysical basis in glucocorticoid receptor evolution. Nature 512, 203–207 (2014). 24930765 10.1038/nature13410 PMC4447330

[92] Karageorgi M., , Genome editing retraces the evolution of toxin resistance in the monarch butterfly. Nature 574, 409–412 (2019). 31578524 10.1038/s41586-019-1610-8 PMC7039281

[93] Tarvin R. D., , Interacting amino acid replacements allow poison frogs to evolve epibatidine resistance. Science 357, 1261–1266 (2017). 28935799 10.1126/science.aan5061 PMC5834227

[94] Weinreich D. M., Delaney N. F., DePristo M. A., Hartl D. L., Darwinian Evolution Can Follow Only Very Few Mutational Paths to Fitter Proteins. Science 312, 111–114 (2006). 16601193 10.1126/science.1123539

[95] Chen S., Zhou Y., Chen Y., Gu J., fastp: an ultra-fast all-in-one FASTQ preprocessor. Bioinformatics 34, i884–i890 (2018). 30423086 10.1093/bioinformatics/bty560 PMC6129281

[96] Li H., Aligning sequence reads, clone sequences and assembly contigs with BWA-MEM. [Preprint] (2013). Available at: http://arxiv.org/abs/1303.3997.

[97] Okonechnikov K., Conesa A., García-Alcalde F., Qualimap 2: advanced multi-sample quality control for high-throughput sequencing data. Bioinformatics 32, 292–294 (2016). 26428292 10.1093/bioinformatics/btv566 PMC4708105

[98] McKenna A., , The Genome Analysis Toolkit: A MapReduce framework for analyzing next-generation DNA sequencing data. Genome Res. 20, 1297–1303 (2010). 20644199 10.1101/gr.107524.110 PMC2928508

[99] DePristo M. A., , A framework for variation discovery and genotyping using next-generation DNA sequencing data. Nat. Genet. 43, 491–498 (2011). 21478889 10.1038/ng.806 PMC3083463

[100] Van der Auwera G. A., , From FastQ Data to High-Confidence Variant Calls: The Genome Analysis Toolkit Best Practices Pipeline. Curr. Prot. Bioinf. 43, 11.10.1–11.10.33 (2013). 10.1002/0471250953.bi1110s43 PMC4243306 25431634

[101] Caetano-Anolles D., GenotypeGVCFs and the death of the dot (obsolete as of GATK 4.6.0.0). GATK (2024).

[102] Danecek P., , Twelve years of SAMtools and BCFtools. Gigascience 10, giab008 (2021). 33590861 10.1093/gigascience/giab008 PMC7931819

[103] Purcell S., , PLINK: A Tool Set for Whole-Genome Association and Population-Based Linkage Analyses. Amer. J. Hum. Genet. 81, 559–575 (2007). 17701901 10.1086/519795 PMC1950838

[104] Alexander D. H., Novembre J., Lange K., Fast model-based estimation of ancestry in unrelated individuals. Genome. Res. 19, 1655–1664 (2009). 19648217 10.1101/gr.094052.109 PMC2752134

[105] Schiffels S., Wang K., “MSMC and MSMC2: The Multiple Sequentially Markovian Coalescent” in Statistical Population Genomics, Dutheil J. Y., Ed. (Springer US, 2020), pp. 147–166. 10.1007/978-1-0716-0199-0_7 31975167

[106] Danecek P., , The variant call format and VCFtools. Bioinformatics 27, 2156–2158 (2011). 21653522 10.1093/bioinformatics/btr330 PMC3137218

[107] Schmidt J. M., de Manuel M., Marques-Bonet T., Castellano S., Andrés A. M., The impact of genetic adaptation on chimpanzee subspecies differentiation. PLOS Genet. 15, e1008485 (2019). 31765391 10.1371/journal.pgen.1008485 PMC6901233

[108] Hernández-Ávila S. G., Hoagstrom C. W., Matamoros W. A., Historical biogeography of North American killifishes (Cyprinodontiformes) recapitulates geographical history in the Gulf of México watershed. Zool. J. Linn. Soc. 202, zlae105 (2024).

[109] Durand E. Y., Patterson N., Reich D., Slatkin M., Testing for Ancient Admixture between Closely Related Populations. Mol. Biol. Evol. 28, 2239–2252 (2011). 21325092 10.1093/molbev/msr048 PMC3144383

[110] Feng C., Wang J., Liston A., Kang M., Recombination Variation Shapes Phylogeny and Introgression in Wild Diploid Strawberries. Mol. Biol. Evol. 40, msad049 (2023). 36864629 10.1093/molbev/msad049 PMC10015625

[111] Morales-Cruz A., , Introgression among North American wild grapes (Vitis) fuels biotic and abiotic adaptation. Genome Biol. 22, 254 (2021). 34479604 10.1186/s13059-021-02467-z PMC8414701

[112] Quinlan A. R., Hall I. M., BEDTools: a flexible suite of utilities for comparing genomic features. Bioinformatics 26, 841–842 (2010). 20110278 10.1093/bioinformatics/btq033 PMC2832824

[113] Cingolani P., , A program for annotating and predicting the effects of single nucleotide polymorphisms, SnpEff: SNPs in the genome of Drosophila melanogaster strain w1118; iso-2; iso-3. Fly 6, 80–92 (2012). 22728672 10.4161/fly.19695 PMC3679285

[114] Schumer M., , Natural selection interacts with recombination to shape the evolution of hybrid genomes. Science 360, 656–660 (2018). 29674434 10.1126/science.aar3684 PMC6069607

[115] Rueden C. T., , ImageJ2: ImageJ for the next generation of scientific image data. BMC Bioinform. 18, 529 (2017). 10.1186/s12859-017-1934-z PMC5708080 29187165

[116] Bates D., Mächler M., Bolker B., Walker S., Fitting Linear Mixed-Effects Models Using lme4. J. Stat. Softw. 67, 1–48 (2015).

[117] Lenth R. V., emmeans: Estimated Marginal Means, aka Least-Squares Means (2021).

[118] Van der Auwere G. A. & O’ Connor B. D.. Genomics in the cloud: using docker, GATK, and WDL in Terra. (Sebastopol, CA O’Reilly Media, 2020).

[119] McKenna A. , The genome Analysis Toolkit: a MapReduce framework for analyzing next-generation DNA sequencing data. Genome Res. 20, 1297–1303 (2010). 20644199 10.1101/gr.107524.110 PMC2928508

[120] DePristo M. A. , A framework for variation discovery and genotyping using next-generation DNA sequencing data. Nat. Genet. 43, 491–498 (2011). 21478889 10.1038/ng.806 PMC3083463

