# Parallel and non-parallel features of adaptive radiation in Yucatán pupfishes

**DOI:** 10.1101/2025.11.17.688971

**Published:** 2025-12-27

**Authors:** Matthew C. Kustra, David Tian, M. Fernanda Palominos, Feifei Guo, Dylan Chau, Oskar Golwala, HoWan Chan, Andrés Alvarez Zapata, Reyna Guadalupe Cetz Paredes, Frida Ximena Cortés Sánchez, Sonia Gabriela Hernández, Adan Fernando Mar-Silva, Fernando Mex, Charles Tralka, Maribel Badillo-Alemán, Juan J. Schmitter-Soto, Carlos A. Gracida-Juárez, Christopher M. Martinez, Jairo Arroyave, Christopher Herbert Martin

**Affiliations:** 1 Department of Integrative Biology, University of California, Berkeley, CA, United States; 2 Museum of Vertebrate Zoology, University of California, Berkeley, CA, United States; 3 Miller Institute for Basic Research in Science, University of California, Berkeley, CA, United States; 4 Department of BioSciences, Rice University, Houston, TX, United States; 5 Instituto Tecnologico Superior de Conkal, Yucatan, Mexico; 6 Instituto de Biología, Universidad Nacional Autónoma de México, Mexico City, Mexico; 7 Unidad Multidisciplinaria de Docencia e Investigación, Unidad Sisal, Universidad Nacional Autónoma de México, Sisal, Yucatán, Mexico; 8 Departamento de Sistemática y Ecología Acuática, El Colegio de la Frontera Sur, Chetumal, Quintana Roo, Mexico; 9 Subdirección de Posgrado e Investigación, Tecnológico Nacional de México, Campus Felipe Carrillo Puerto, Felipe Carrillo Puerto, Quintana Roo, Mexico.; 10 Department of Ecology and Evolutionary Biology, University of California, Irvine, CA, United States

**Keywords:** Speciation, De novo mutations, Introgression, Genomics, Adaptation

## Abstract

Understanding the extent of parallelism across adaptive radiations remains a central problem in evolutionary biology. We used whole-genome resequencing of 123 individuals to compare the adaptive radiation of *Cyprinodon* pupfishes in Lake Chichancanab, Mexico, to an independent radiation of San Salvador Island (SSI) pupfishes in the Bahamas, and assess the repeatability of adaptive genetic architecture, sources of adaptive variation, and stages of selection. Despite rapid craniofacial divergence of trophic specialists within 8–15 kya, only two candidate genes (0.5%; 2/426) were shared between Caribbean radiations. Although adaptive introgression played a major role in SSI, we found minimal evidence of adaptive introgression in Chichancanab, likely due to the geographic isolation of this inland lake. Instead, de novo mutations provided a substantial source of adaptive variation (30.6%) for the endemic zooplanktivore, 15 times higher than the endemic scale-eater on SSI. However, in parallel with SSI, we found strong evidence that adaptive divergence occurred in stages, first on regulatory and standing genetic variation, then on de novo and nonsynonymous mutations. Consistent with adaptive variants near opsin and spermatogenesis genes, functional categories unique to Chichancanab, we found greater visual acuity and divergent sperm morphology in lab-reared zooplanktivores relative to generalists using laboratory assays. Consistent with extensive adaptive de novo mutations in *WNT10A* and rapid diversification of tooth size in the zooplanktivore, we found that experimental inhibition of the Wnt pathway in generalists resulted in narrower oral teeth. We conclude that de novo mutations, not introgression, can drive rapid adaptive radiations in isolated environments.

## Introduction

Much of biodiversity results from adaptive radiation—when a clade diversifies rapidly into three or more new species (1–5). Young adaptive radiations provide excellent systems for investigating speciation and adaptation (3, 6–9). However, it remains unclear to what degree radiations occur in parallel given similar starting positions on the adaptive landscape with similar levels of genetic diversity, gene flow, and ecological opportunity (10, 11).

Beneficial de novo mutations can provide novel variation to reach new fitness peaks on the adaptive landscape during adaptive radiation (12, 13). However, the longer time needed for de novo beneficial mutations to arise contradicts the short timescales of rapid divergence (11, 14, 15). Hybridization can introduce large amounts of genetic variation and novel phenotypes (transgressive segregation) through adaptive introgression and hybrid swarms, enabling populations to reach previously inaccessible fitness peaks (16). Support for an important role of hybridization in adaptive radiations has been demonstrated both theoretically (17, 18) and empirically in many systems (3, 19–27). However, we still do not know the relative importance of different sources of genetic variation for triggering and sustaining adaptive radiations across time and space.

Caribbean pupfishes are a remarkable system for assessing the repeatability of adaptive radiations due to two independent, recent adaptive radiations, each containing a generalist and multiple trophic specialists within isolated lake environments that have historically lacked predatory fishes and most competitors except *Gambusia*. The San Salvador Island (SSI), Bahamas radiation proceeded predominantly via the reassembly of pre-existing standing genetic variation found throughout the Caribbean, with substantial contributions of adaptive introgression to both the molluscivore (durophage) and scale-eating (lepidophage) specialists. Only 0% and 2% of adaptive mutations in the molluscivore and scale-eater, respectively, were de novo mutations unique to SSI (26, 28–32). However, it was previously unknown how a second radiation in Lake Chichancanab, Mexico, drawing from the same Caribbean pool of standing genetic variation, unfolded over the same timeframe.

**Figure 1.**
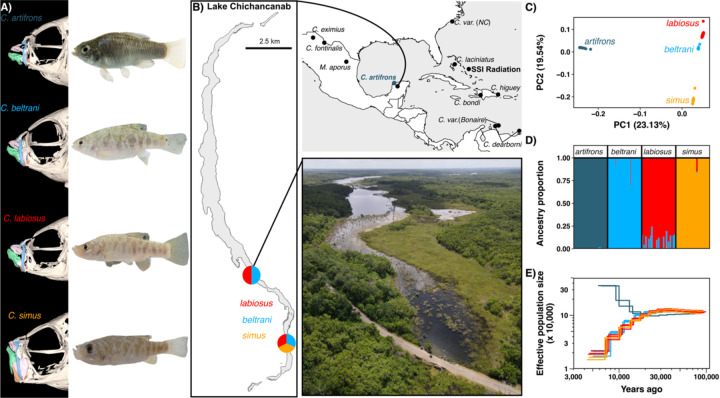
Yucatán *Cyprinodon* pupfishes show strong genetic differentiation. (A) µCT scans of Yucatán pupfish with maxilla colored in blue, premaxilla in pink, dentary in green, and articular in orange (see Figure S1 for different views). (B) Map of the Caribbean showing the sampling sites for all species in our dataset with an inset map of Lake Chichancanab and an aerial view image from one of the sampling sites. (C) Principal component analysis using a set of linkage disequilibrium pruned SNPs. (D) Estimated ancestry proportions for each individual inferred using *ADMIXTURE* with K = 4 (see Figure S3 for K=2–3) from the linkage-pruned dataset. (E) Estimated demographic history with *MSMC2* on a log scale. Each colored line represents an individual, with colors corresponding to different species.

Here, we use whole-genome resequencing of 123 individuals to provide insight into the genomic architecture and genetic origins of the Lake Chichancanab *Cyprinodon* pupfish radiation (33). This recent adaptive radiation (~8000 years old (34)) consisted of at least five species (or up to seven (35)) historically (33, 35, 36), including a detritivore (*C. beltrani*), piscivore (*C. maya*), rocky substrate specialist (*C. verecundus*), bivalve specialist (*C. labiosus*), and zooplanktivore (*C. simus*). At the time of description (33), the relative abundances of these endemic species were 68–85% *C. beltrani*, 6–18% *C. maya*, 2–13% *C. labiosus*, and <1% *C. simus* and *C. verecundus*. However, due to recent colonization by invasive African tilapia (*Oreochromis niloticus x mossambicus*) (37, 38) and by several species native to surrounding basins but not Chichancanab (*Astyanax angustifrons, Mayaheros urophthalmus, Poecilia mexicana*; pers. obs.), all trophic specialist species now appear to be extinct since our collections in 2022, with only the generalist, *C. beltrani*, remaining in 2024. Despite exhibiting one of the fastest rates of trait diversification of any vertebrate in tooth size (130 times faster than background rates across *Cyprinodon* (29)) and rapid divergence in other craniofacial morphology (Figure 1A; S1), previous genetic studies of microsatellite data and mtDNA suggest that this radiation has low genetic differentiation (39, 40), enabling the identification of highly differentiated loci among these species.

## Results

### Genetic differentiation of the species flock

To measure population structure of the Yucatán *Cyprinodon* pupfishes, we conducted whole genome resequencing of the species flock and the coastal sister species (*n* = 15 *C. artifrons*, *n* = 35 *C. beltrani*, *n* = 35 *C. labiosus*, and *n* = 17 *C. simus*) using specimens collected from the wild in 2022 (Figure 1B). We aligned these samples, as well as eleven other previously sequenced Caribbean pupfish species, to the high-quality *Cyprindon nevadensis mionectes* reference genome (GCA_030533455.1). After calling and filtering variants, our dataset included 23,960,536 SNPs (12.4x mean depth). Using a linkage disequilibrium pruned set of SNPs (*n* = 5,476,855 SNPs), we used principal component analysis and *ADMIXTURE* to visualize population structure within Yucatán pupfishes. We found that all species formed distinct, non-overlapping genetic clusters, with *C. artifrons* being the most genetically divergent from and sister to the Chichancanab species (Figure 1C). Within the Chichancanab species flock, *C. simus* was more genetically divergent from *C. labiosus* and *C. beltrani* (Figure 1C). These results were also supported by genome-wide *F*_*ST*_ estimates and a phylogenetic tree constructed with *ADMIXTOOLS2* (Table S1; Figure S2). Four clusters corresponded to the four different species, as determined by *ADMIXTURE* (Figure 1D). All *C. artifrons* individuals showed minimal evidence of admixture with Chichancanab species (Figure 1D; Figure S3).

### Demographic history

Using *MSMC2* for Chichancanab species with a mutation rate of 1.56 × 10^−8^ substitutions per base pair (estimated from a Caribbean *Cyprinodon* species (26)), we found that the three Chichancanab species (*C. beltrani*, *C. labiosus*, and *C. simus*) diverged from the coastal sister species *C. artifrons* at the end of the Pleistocene 10–15 kya, consistent with receding sea levels resulting in the formation of Lake Chichancanab (34). After divergence from *C. artifrons*, the Chichancanab species flock showed a similar demographic history, characterized by a sharp decline in effective population sizes, consistent with endemic sympatric species within an isolated lake (Figure 1E).

### Adaptive loci within trophic specialists

**Figure 2.**
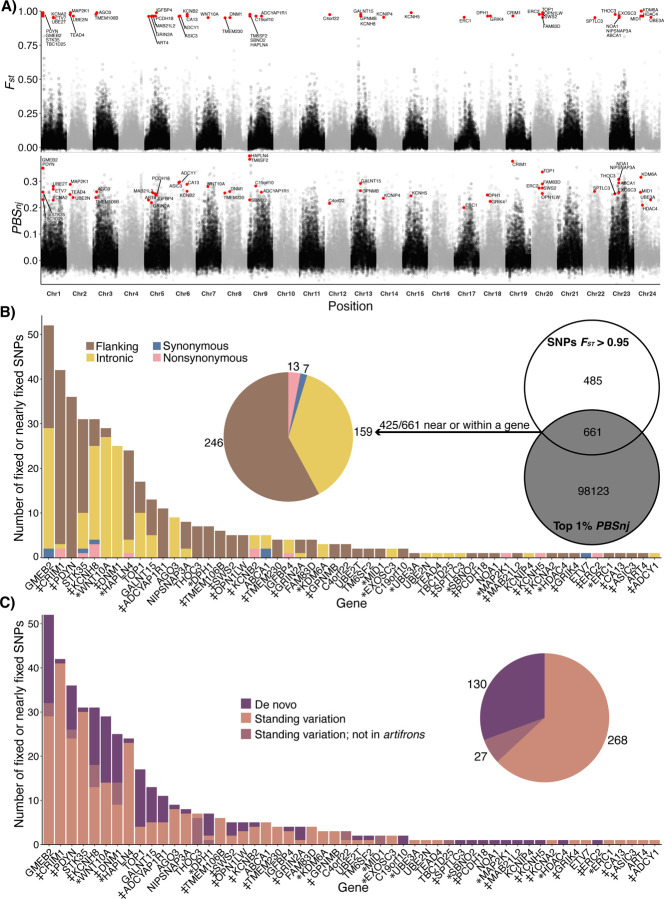
Architecture and source of adaptive genetic variation in the zooplanktivore. (A, top row) *F*_*st*_ between *C. simus* and *C. beltrani* + *C. labiosus* calculated in non-overlapping 10 kb windows. *F*_*st*_ values for candidate SNPs (red circles) were calculated separately per locus to show the location of fixed variants. (A, lower row) Population branch statistics (*PBS*_*nj*_) with *C. simus* as the target population versus *C. beltrani*, *C. labiosus*, and *C. artifrons* in 10-kb non-overlapping windows. Candidate genes within 20 kb of fixed or nearly-fixed (Fst > 0.95) SNPs and within *PBS*_*nj*_ 1% outlier windows are highlighted in red and labeled. Alternating colors represent different chromosomes. (B) Candidate adaptive variants near or within a gene were defined as SNPs that were fixed (*n* = 17) or nearly fixed (*n* = 1,129; *F*_*st*_ > 0.95) and occurred within a 1% population branch statistic (*PBS*_*nj*_) outlier window for *C. simus* (Venn diagram). Pie chart and bar graphs indicate the proportion of these 425 adaptive variants that were found within the flanking, intronic, or coding regions. (C) Pie chart and bar graphs indicate the proportion of these 425 variants that were detected only within Chichancanab (de novo: purple), as standing genetic variation in coastal sister species *C. artifrons* (pink), or in other Caribbean species, but not *C. artifrons*. * Indicates genes with a craniofacial annotation; ‡ indicates genes with neural or sensory annotation.

Although genome-wide differentiation among Chichancanab species was moderately low (*F*_*ST*_ ranging from 0.032 to 0.09; Figure 2A; Table S1), we were able to identify highly differentiated regions of the genome within the trophic specialist zooplanktivore *C. simus*. We identified 19 fixed and 1,127 nearly-fixed (*F*_*ST*_ >0.95) SNPs in *C. simus* relative to other Chichancanab pupfishes (Tables S2–S3). In contrast, we found zero fixed or nearly-fixed SNPs within the generalist *C. beltrani* or bivalve-specialist C. *labiosus*. However, we were able to identify some moderately differentiated SNPs within *C. labiosus* (*n* = 97) and *C. beltrani* (*n* = 13) relative to the other Chichancanab species at an *F*_*ST*_ threshold of 0.8 (Table S2).

To search for signals of selection, we calculated the normalized four-taxon population branch statistic (*PBS*_*nj*_) across 10-kb non-overlapping windows because linkage disequilibrium decayed at this distance (Figure S4). Simulation studies demonstrate that normalized *PBS* is effective for identifying both hard and soft selective sweeps (41). Out of the 1,146 SNPs fixed or nearly fixed within *C. simus*, 661 were within a strongly supported selective sweep window (top 1% *PBS*_*nj*_ outlier (41)), including 425 SNPs within 20 kb of the first or last exon of an annotated gene. These 425 candidate adaptive SNPs (“adaptive variants”) were located in the proximity of, or within, 55 genes (Figure 2A), distributed across 19 out of 24 chromosomes (Figure 2A). Adaptive variants associated with the same gene were often distributed across multiple introns or exons and spaced apart by 1kb or more (Figure S5, Table S4).

### Source and function of adaptive genetic variation in the zooplanktivore

We next characterized the location of the 425 candidate adaptive variants near or within a gene in the zooplanktivore *C. simus*. More than 95% were in intronic or flanking regions (20-kb upstream or downstream of a gene; Figure 2B). Within coding regions, 13 out of 20 adaptive variants were nonsynonymous mutations (Figure 2B). The number of adaptive variants per gene followed an exponential distribution, with a handful of genes containing many variants and most genes with only a single adaptive variant (Figure 2B). We functionally characterized these genes by manually checking online databases and doing a literature search (Table S3; Figure 2B). 32 out of the 55 candidate genes were associated with craniofacial development (*n =*11; 20%) or sensory/neural function (*n =* 21; 38.2%). We manually checked if any of these genes overlapped with the 426 unique candidate genes identified in the SSI radiation (26, 28, 30, 32, 42–46), and we found only two overlapping candidate genes (*DNM1* and *SPTLC3*), both of which are associated with motor neuron development (47, 48) and are scale-eater-specific candidate genes.

Next, we characterized the source of the variants and found that 63% of the adaptive SNPs in *C. simus* also existed as standing genetic variation in the coastal Yucatan population of *C. artifrons* (“standing variation”), 31% were *de novo* variants found only in Chichancanab, and 6% were detected as standing genetic variation in other pupfish species but not *C. artifrons* (Figure 2C).

### Limited adaptive introgression within Chichancanab pupfishes

**Figure 3.**
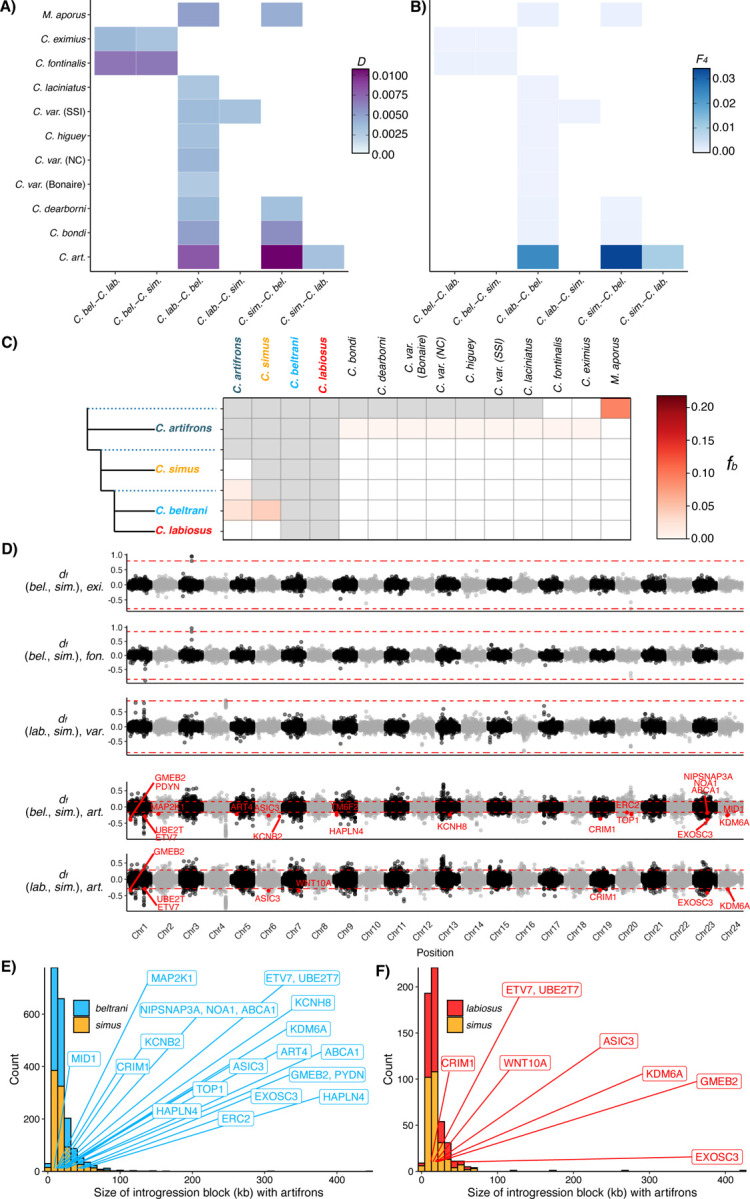
Minimal evidence of adaptive introgression into Chichancanab pupfishes. Heatmaps of (A) Patterson’s *D*-statistics and (B) *F*_*4*_ ratios for P1-P2 species combinations within the Chichancanab radiation along the x-axis and P3 focal species along the y-axis. All four-taxon trees used *Cualac tessellatus* as the outgroup (P4). Only significant (*Z*-score > 3) results are colored; combinations not relevant to the Chichancanab radiation are not shown. (C) *f-*branch (*f*_*b*_) statistics indicate introgression occurring before the Chichancanab radiation. Red boxes indicate significant introgression between P3 species (top row) and the focal species or internal branch (dashed lines) on the left. Non-significant *f*_*b*_ statistics are indicated by white boxes. Grey boxes represent comparisons that cannot be made since *f*_*b*_ cannot be calculated for introgression between sister taxa. See Figure S6 for full *f-*branch graphs. (D) Genome-wide plot of the distance fraction (*d*_*f,*_, an introgression test statistic (50)) calculated in non-overlapping windows of 91 SNPs (approximately 10 kb) for species trios that showed evidence of introgression with *C. simus. d*_*f*_ outlier windows that contained a candidate gene under selection in *C. simus* are colored red and labeled with the gene name. The y-axis label gives the introgression topology: (P1, P2), P3. Positive values indicate regions of introgression between *C. simus* and P3 (from top to bottom: *C. eximius*, *C. fontinalis*, *C. variegatus* (SSI), and *C. artifrons* in the bottom two subplots); negative values indicate regions of introgression between P1 (*C. beltrani* or *C. labiosus*) and P3. Red dashed lines indicate outlier cutoffs, which were determined empirically by the overall percentage of genome-wide introgression estimated from the *F*_*4*_ ratios in panel B. (E, F) Distribution of the size of *d*_*f*_ outlier introgression tract lengths (in 10 kb windows) between *C. artifrons* and Chichancanab species. The introgression tracts containing candidate adaptive genes in *C. simus* are labeled with gene names. SSI: San Salvador Island, Bahamas; NC: North Carolina, USA.

To test for introgression into the Chichancanab radiation, we used *f*-statistics calculated in *Dsuite* (49) to examine gene flow from 11 additional *Cyprinodon* species from across the clade and the sister species to the genus *Megupsilon aporus*. Within Yucatán pupfishes, we found evidence of introgression between *C. artifrons* and both *C. beltrani* and *C. labiosus*, with the strongest signal in *C. beltrani* (Figure 3A, B). We also found evidence for introgression between *C. beltrani* and most outgroup species examined, including the sister species to *Cyprinodon*, *Megupsilon aporus*. Relative to *C. beltrani*, *C. labiosus* and *C. simus* also showed evidence of introgression with pupfishes endemic to the Chihuahua desert (*C. eximius* and *C. fontinalis*; Figure 3A, B). Furthermore, we also found evidence of introgression between the two sympatric radiations (*C. variegatus* from SSI) and *C. simus* (Figure 3A, B). However, additional *f-*branch statistics indicate that all patterns of excess allele sharing between these outgroup species besides *C. artifrons* and the Chichancanab species flock likely resulted from ancient introgression events before the colonization of Lake Chichancanab (Figure 3C; S6).

To test for adaptive introgression into *C. simus*, we next examined whether the set of adaptive variants (Figure 2) occurred within outlier introgression windows using sliding window scans of distance fraction (*d*_*f*_ (50)) for species that had significant genome-wide introgression into *C. simus* (Figure 3A, B). We found that none of the outlier introgression windows overlapped with *C. simus* adaptive variants (Figure 3D).

Next, we performed *d*_*f*_ scans for introgression between *C. artifrons* and Chichancanab species. Although there were outlier introgression regions between *C. simus* and *C. artifrons* (positive values *d*_*f*_), these regions did not overlap with *C. simus* adaptive variants (Figure 3D). However, the position of several of these *C. simus* adaptive variants overlapped with outlier introgression windows for both *C. labiosus* and *C. beltrani* but not *C. simus* (Figure 3D). The number of outlier introgression windows was much lower in *C. simus* relative to *C. labiosus* and *C. beltrani* (Figure 3E, F). Furthermore, both *C. labiosus* and *C. beltrani* populations contained several introgression tracts that were much longer than *C. simus* tract lengths, indicating much more recent introgression events in these species (Figure 3E, F).

### The stages of adaptive divergence of the zooplanktivore

**Figure 4.**
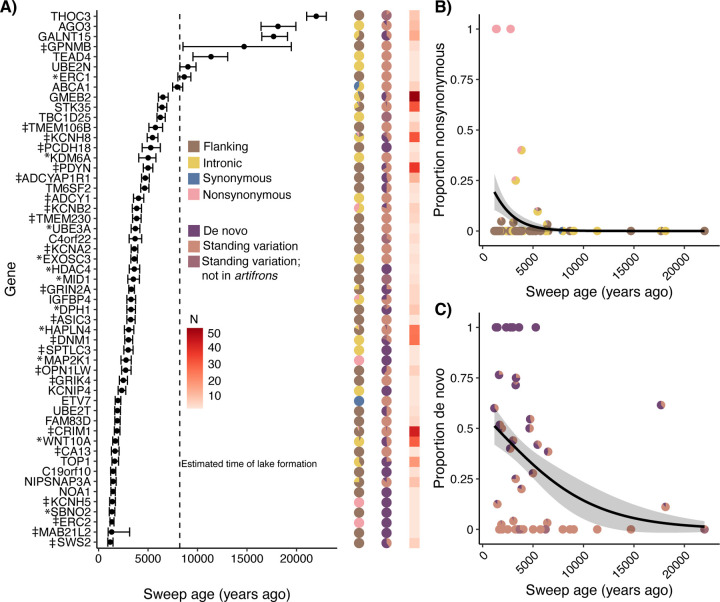
Stages of adaptive divergence in the zooplanktivore. (A) Points indicate median sweep age with 95% credible intervals. The dashed line indicates the approximate age of Lake Chichancanab (34). For each sweep, the first column of pie charts shows the proportions of flanking/intronic/synonymous/nonsynonymous, second column of pie charts shows the proportions of de novo/standing variation, and third column of squares shows the number of candidate adaptive variants per gene (from Figure 2). For genes with multiple adaptive variants, we used the variant with the median position as the focal position. * Indicates genes with a craniofacial annotation; ‡ indicates genes with neural or sensory annotation. (B) The proportion of nonsynonymous adaptive variants is significantly larger in more recent selective sweeps, indicated by the best-fit quasibinomial regression line; each pie chart represents a single candidate gene. (C) The proportion of de novo variants is significantly larger in more recent selective sweeps; each pie chart represents a single candidate gene.

We used *starTMRCA* (51) to estimate the timing of selective sweeps for the set of candidate adaptive variants near genes within the zooplanktivore *C. simus*. We estimated that most selective sweeps occurred in a staggered sequence and not simultaneously after colonization of Lake Chichancanab (Figure 4A)(34). There were also a few older sweeps that we estimate may predate the age of Lake Chichancanab and divergence from the coastal sister species *C. artifrons* (Figure 1B). Overall, we observed no clear temporal stages of adaptive phenotypic divergence related to craniofacial genes (asterisks) or neural/sensory genes (‡; Figure 4A). Instead, we found that selection on these two broad functional categories occurred throughout the period of divergence of *C. simus* following the colonization of Lake Chichancanab, contrary to behavior-first, then craniofacial stages of adaptation (52) found in the SSI radiation (26).

Next, we used generalized linear regressions to test if sweep age was correlated with the proportion of nonsynonymous adaptive variants, de novo adaptive variants, or the overall number of adaptive variants per gene region. We found that both nonsynonymous adaptive variants (χ^2^ = 5.852, *p* = 0.016) and de novo mutations (χ^2^ = 6.221, *p* = 0.013) were significantly more likely to occur in more recent selective sweeps during the final stage of adaptive divergence in *C. simus* (Figure 4B, C). However, nonsynonymous mutations were not significantly more likely to be de novo mutations found only in Chichancanab species (All adaptive variants: χ^2^ = 0.102, *p* = 0.749; singleton adaptive variants: χ^2^ = 2.010, *p* = 0.156), and there was no correlation between estimated sweep age and the number of candidate adaptive variants per gene region (χ^2^ = 0.111, *p* = 0.740).

### Increased visual acuity in the zooplanktivore is consistent with vision-related candidate adaptive genes

**Figure 5.**
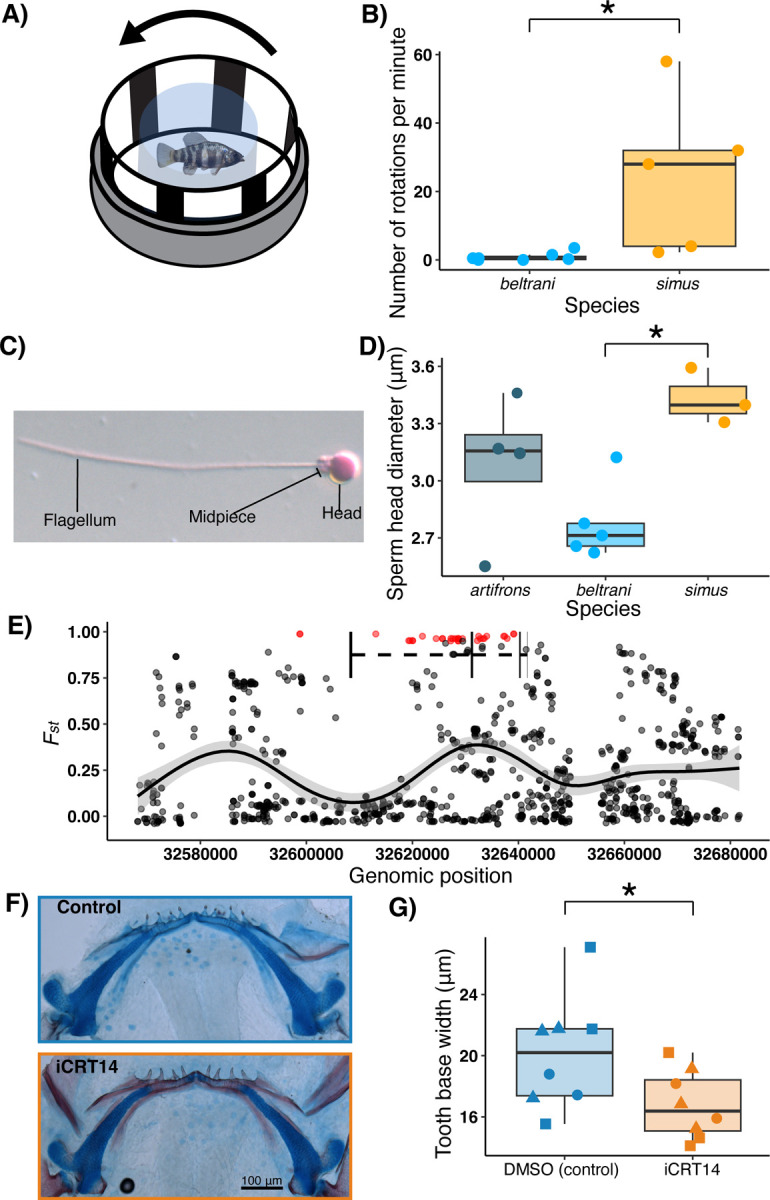
Divergence in visual acuity, sperm morphology, and tooth development reflects candidate adaptive genes in the zooplanktivore. (A) Diagram of the optomotor response experimental apparatus (adapted from (56)). (B) Boxplot and jittered points of the number of complete rotations fish swam per minute, indicative of their optomotor response to black-and-white vertical bars spinning at a constant rate. (C) Representative labeled image of a single *C. beltrani* sperm at 100x stained with rose bengal. (D) Boxplots with jittered points of sperm head diameter for *C. artifrons, beltrani,* and *simus.* Significant Tukey’s post hoc differences are shown with an asterisk. (E) Manhattan plot showing per-site *F*_*st*_ of SNPs within 50 kb of *WNT10A*. Black rectangles indicate exons of *WNT10A*, and dashed lines indicate introns. Red points represent nearly fixed SNPs (*F*_*st*_ > 0.95), and black points represent all other SNPs. The black line is the smoothed average *F*_*st*_ in this region, with grey shading being the 95% confidence intervals. (F) Representative images of *C. beltrani* larvae that were treated with DMSO (control; top) or with iCRT14 Wnt inhibitor + DMSO (bottom) and stained with alizarin red (bone) and alcian blue (cartilage). (G) Boxplot and jittered points of average tooth base width of the left and right teeth closest to the mandibular symphysis for *C. beltrani* larvae treated with a DMSO control or Wnt inhibitor (iCRT14) + DMSO. Shapes represent three independent split-brood replicates.

We identified several *C. simus* adaptive variants associated with eye development (*ASIC3*(53), *CRIM1*(54), *MAB21L2*(55)) and opsin (*OPN1LW*, *SWS2*) genes. Because the trophic specialization of *C. simus* requires visually locating zooplankton in the water column before precise suction-feeding strikes, we hypothesized that this species would have higher visual acuity than the detritivore, *C. beltrani*. We conducted optomotor response trials on lab-reared individuals from both species (F1 *C. beltrani* and long-term laboratory colony of F10+ *C. simus*) and found that *C. simus* has a much stronger optomotor response than *C. beltrani* in response to black-and-white vertical bars spinning at a constant rate of approximately 90 rotations per minute (Figure 5A, B; Wilcoxon signed-rank test: *W* =1, *p* = 0.009). There was no significant difference in rotations per minute between the species for the same individuals during control observation periods with no spinning (Figure S7A; *W*=14, *p* = 0.3105) or during positive control observation periods with an all-black background spinning at the same frequency (Figure S7B; *W* =9, *p* = 0.147).

### Divergent sperm morphology of the zooplanktivore is consistent with spermatogenesis-related candidate adaptive genes

Four candidate genes (*C4orf22*, *STK35*, *ERC2*, and *TBC1D25*) are known to affect spermatogenesis/fertility (57–60). We therefore examined whether three Yucatan species, raised in a common garden laboratory environment (F1 *C. artifrons,* F1 *beltrani,* and a long-term laboratory colony of F10+ C. *simus*), differed in sperm morphology using differential interference contrast microscopy. We found that species significantly differed in sperm head length (Figure 5D; *F* = 5.724, *p* = 0.025), but not sperm midpiece length (Figure S8A; *F* = 2.403, *p* = 0.147) or flagellum length (Figure S8B; *F* = 0.414, *p* = 0.673). *C. simus* had significantly larger sperm heads than *C. beltrani* using Tukey’s post hoc pairwise comparisons (*q* = 3.367; *p* = 0.0207; Fig. 5D).

### Inhibition of WNT10A alters tooth morphology in Chichancanab pupfish

We identified a large number of highly differentiated SNPs surrounding the well-characterized *WNT10A*, which controls tooth, hair, and skin development within the Wnt signaling pathway (Figure 2; Figure 5E (61–63)). This is consistent with the most exceptional morphological difference between *C. simus* and *C. beltrani,* extreme divergence in tooth size (130 times faster than background rates (29)).

To test for the importance of *WNT10A* in tooth development within pupfishes, we used iCRT14 to inhibit the Wnt / β catenin pathway downstream of WNT10A during metamorphosis in *C. beltrani* lab-reared fry (64, 65). Chemical inhibition from 8 dpf to 22 dpf resulted in narrower teeth than control fry treated with DMSO (χ^2^ = 4.840, *p* = 0.0278; Figure 5F, G). There were no significant differences in mean tooth length (χ^2^ = 0.455, *p* = 0.500; Figure S9) or number of ossified teeth when stained with alizarin red (χ^2^ = 0.114, *p* = 0.7357; Figure S10).

## Discussion

Here, we investigated the genetic architecture, sources of adaptive variation, and temporal dynamics of selection underlying speciation and trophic specialization in a young adaptive radiation of *Cyprinodon* pupfishes endemic to Lake Chichancanab, Mexico. By comparing these patterns to our previous work on a recent radiation of *Cyprinodon* pupfishes in SSI, Bahamas (26, 28–32), we discovered parallel and non-parallel features of these independent radiations in similar environments. Despite replicate radiations of Caribbean pupfishes in large, isolated, predator-free, saline lakes with few competitors, we found little parallelism between the radiations. Although craniofacial morphology diverged rapidly within trophic specialists in both radiations, we found almost zero overlap in the adaptive genes underlying trophic specialization (0.5%), potentially reflecting the divergent specialists within each radiation. In contrast to SSI, there was no behavior-first stage of adaptive divergence in Chichancanab specialists and, with the exception of craniofacial development, candidate adaptive gene ontologies potentially relevant to speciation were not shared between radiations, such as pigmentation genes in SSI versus opsin and spermatogenesis genes in Chichancanab. Unlike the SSI radiation and most other well-studied adaptive radiations (e.g., Malawi cichlids (20), Galápagos finches (66)), adaptive introgression likely played a minimal role in the Chichancanab radiation. We found no overlap between adaptive variants in the specialists and tracts of introgression. Instead, over 30% of the candidate adaptive variants in the zooplanktivore, *C. simus*, likely arose from de novo mutations compared to only 2% in the SSI scale-eater specialist and none in the molluscivore specialist. One outstanding parallel feature was a clear stage of refinement in the adaptive divergence of the zooplanktivore, marked by increased rates of de novo and nonsynonymous adaptive mutations occurring among the most recent selective sweeps in this species, similar to the scale-eating specialist on SSI. In contrast to *C. simus*, we identified no sufficiently differentiated SNPs within *C. labiosus*, the bivalve specialist, to identify candidate genes. Nonetheless, minimal divergence of this specialist in Chichancanab parallels the minimal divergence of the molluscivore on SSI, each in contrast to a more divergent trophic specialist.

### Adaptive introgression did not contribute to speciation of Chichancanab pupfishes

Genomic analyses have revealed that hybridization and adaptive introgression played a major role in many adaptive radiations (3, 11, 16, 19–27, 67, 68), including the *Cyprinodon* SSI radiation (26, 31). However, in Chichancanab, we found minimal evidence of introgression overall and did not detect any signatures of adaptive introgression following the colonization of Lake Chichancanab. Although our analyses indicate extensive introgression among *Cyprinodon* and *Megupsilon* lineages, we did not find any introgression events into the root of the radiation, nor has there been secondary gene flow from outgroup species other than the sister species to the radiation, *C. artifrons*. Secondary gene flow from *C. artifrons* was almost entirely detected with *C. beltrani* and, to a lesser extent, *C. labiosus.* Gene flow may be higher with these species because *C. artifrons,* a coastal benthic feeding detritivore, is less ecologically divergent from both *C. beltrani,* a benthic feeding detritivore, and *C. labiosus*, a benthic feeding bivalve specialist. Secondary gene flow between *C. artifrons* and both *C. beltrani* and *C. labiosus* overlapped with many genes under selection in *C. simus*, suggesting that secondary gene flow is helping increase similarities between the benthic detritivores within the lake and the coastal *C. artifrons* population rather than by bringing in adaptive variation for trophic specialization in *C. simus*.

These results are remarkably different from the *Cyprinodon* adaptive radiation of SSI, where there is strong evidence of introgression into the root of the radiation and substantial evidence of secondary adaptive introgression into both trophic specialists from at least three different biogeographic regions of the Caribbean and Atlantic coast (26, 31). Lake Chichancanab is an endorheic basin with no above-ground hydrological connections to the ocean and is substantially more geographically isolated than SSI, a small island in the approximate center of the Caribbean. Furthermore, the sister species to the Chichancanab species flock, *C. artifrons*, is abundant along the entire coast of the Yucatán peninsula, suggesting that any gene flow into Chichancanab would likely first have to pass through *C. artifrons* populations. An additional factor is the water chemistry of Lake Chichancanab which is near-saturation with calcite and gypsum and may be a greater physiological barrier to marine pupfish colonists than the slightly hypersaline lakes of SSI (33–36). Thus, geography and ecology play an important role in determining the relative importance of adaptive introgression in radiations.

### De novo mutations primarily drive adaptive divergence of the zooplanktivore

The importance of de novo mutations in adaptive radiations remains contentious, in part due to the time needed for de novo beneficial mutations to arise (11, 15). Interestingly, we found that 30.6% of the zooplanktivore adaptive variants were detected only within Chichancanab species, suggesting their de novo origins within the basin. This proportion is 15.3 times greater than the contribution of de novo mutations to adaptive divergence of the scale-eater in the SSI radiation. A primary factor could be that the estimated effective population size for the Chichancanab radiation is approximately twice that of the SSI radiation (26), indicating that selection on de novo mutations is more effective, with a higher probability of fixation, all else being equal (14). There may also be a lack of existing standing genetic variation within Caribbean pupfish populations that is adaptive for zooplanktivory. In support of this hypothesis, although radiations should proceed along genetic lines of least resistance (69), the primary axis of craniofacial morphological variation along which the zooplanktivore diverged is far removed from the first two major axes of craniofacial variation within Caribbean pupfish species (29). Evolving along the line of greatest resistance instead of least resistance may require de novo mutations. While the SSI scale-eater is also highly divergent, it is closer to existing craniofacial variation within Caribbean pupfish species and in the direction of greatest variance along the first two principal component axes (29). Similarly, mutational target size for the craniofacial features of a zooplanktivore may be much smaller than the features of a scale-eater, resulting in less standing genetic variation available for adaptation to this trophic niche. Alternatively, we may not have detected these de novo mutations in other populations, but this is unlikely to explain all de novo mutations given our extensive sampling of *Cyprinodon* species across the clade and spanning the Caribbean (Figure 1B). Thus, our results highlight that de novo mutations may play a more important role in driving young adaptive radiations than currently appreciated (12, 13), especially when ecotypes are highly divergent from other species within the radiation.

### Minimal parallelism in the genes underlying adaptive divergence of trophic specialists

We found only two candidate genes (*DNM1* and *SPTLC3*) among the 55 zooplanktivore candidate genes in the Chichancanab radiation that overlapped with the hundreds of candidate genes identified so far in the SSI radiation (26, 28, 30, 32, 42–46). Consistent with trophic specialization requiring the pursuit of mobile prey, both genes are involved in motor neuron development. *DNM1* is a dynamin with nervous tissue-specific expression that is crucial for the proper formation of motor neuron axons (47). *SPTLC3* is involved in the biosynthesis of sphingolipids (70), and suppression of *SPTLC3* in zebrafish results in motor neuron axon defects (48).

The extremely limited number of shared adaptive genes is surprising given the high conservation of gene function in both morphological development (71, 72) and behavior (73), and the frequent parallelism of candidate genes across vertebrate adaptive radiations, particularly for craniofacial genes. For example, *BMP4* is involved in diversification in both Galápagos finches (74) and African cichlids (75, 76). Furthermore, within the SSI radiation, many of the same genes show parallel patterns of differential expression even within divergent trophic specialists (26, 43). Nonetheless, parallelism of shared regulatory networks, rather than specific genes, is still a possibility.

### Rapid divergence in tooth morphology of the zooplanktivore through WNT10A

Another reason for non-parallelism is that trophic specialists in each radiation have diverged in different sets of traits during adaptation to different ecological niches (29). Specifically, the Chichancanab radiation has diversified in tooth size over 130 times faster than background rates, driven by the extremely short and narrow teeth of the zooplanktivore (Figure S1; (29). We identified a well-characterized tooth development gene in the Wnt pathway, *WNT10A*, with many de novo mutations in flanking (*n* = 2) and intronic (*n* = 13) regions. Mutations in *WNT10A* are associated with tooth developmental disorders in humans (62) and both knockout and overexpression experiments in sticklebacks and zebrafish have demonstrated *WNT10A*’s role in teeth development and regeneration (61, 63). Consistent with *WNT10A* playing a role in rapid divergence of tooth morphology, we found that inhibiting the Wnt pathway in the *C. beltrani* generalist during pre-metamorphosis development resulted in narrower teeth, similar to the reduced tooth sizes in the zooplanktivore *C. simus* (Figure 5C). This indicates that the substantial number of de novo mutations in *WNT10A* may be suppressing the Wnt pathway, resulting in a substantial reduction in tooth size in the zooplanktivore.

### Increased visual acuity in the zooplantkivore is consistent with unique selection on vision-related genes

One of the more striking features of both *Cyprinodon* radiations is the evolution of novel trophic specialists from a generalist ancestor: the scale-eater (*C. desquamator*) in the SSI radiation and the zooplanktivore (*C. simus*) in the Chichancanab radiation. Although these are distinct dietary specializations, they share a similarity in that they require tracking free-swimming prey. Indeed, four of the top 15 enriched GO terms of candidate genes in the SSI scale-eater were related to eye development (26). Similarly, several of the candidate genes we identified in the zooplanktivore were related to eye development, including *ASIC3*, *MAB21L2*, and *CRIM1*. *CRIM1* is the most promising candidate because it is one of three candidate genes with multiple nonsynonymous mutations (Figure 2). In zebrafish, a two-base pair deletion in *CRIM1* resulted in abnormal lenses as well as a large eye-to-head ratio (54). Interestingly, one of the defining morphological features of *C. simus* is its large eye-to-head ratio (33) (Figure 1A). Consistent with candidate genes related to eye development, we confirmed that *C. simus* exhibits a much stronger optomotor response than *C. beltrani* (Figure 5B), indicating that this specialist has greatly improved visual acuity, even when reared in a common laboratory environment.

Although both radiations contained many vision-related candidate genes, selection on opsin genes was unique to Chichancanab. Most notably, we found several adaptive variants associated with *SWS2* (blue-sensitive), and *OPN1LW* (red-sensitive) in the zooplanktivore. Interestingly, both opsins contain many candidate de novo mutations, and *SWS2* is the youngest selective sweep in the zooplanktivore. One explanation could be that most Caribbean *Cyprinodon* species are benthic and live in similar habitats, resulting in limited standing genetic variation in opsins. Additionally, most environments (including SSI and Chichancanab) are relatively shallow, so differences in depth—a common selective pressure on opsin evolution (77)—are likely not a strong selective force on SSI. Thus, the main selective force acting on vision in Chichancanab may be prey capture efficiency. In many fish species, zooplanktivory is associated with higher expression of shorter wavelength opsins (e.g., *SWS1* (77)), whereas piscivory is not often associated with changes in opsin expression (78, 79).

### Divergent sperm morphology in Chichancanab species is consistent with selection on spermatogenesis genesis

Although the role that premating sexual selection plays in speciation has historically been the main focus (80, 81), there is both theoretical and empirical support for the role of postmating sexual selection in maintaining reproductive isolation via postmating prezygotic reproductive barriers (82–87). In the SSI radiation, species differ in pigmentation, and a handful of the candidate adaptive variants fall in regulatory regions of genes associated with pigmentation (26), but not spermatogenesis. Furthermore, there is evidence for premating isolation by female preference in the scale-eater (88). However, in Chichancanab, the two species tested in our data set (*C. labiosus* and *C. beltrani*) were not found to visually distinguish between different species in the radiation in laboratory mate choice trials (89).

Unlike the SSI radiation, we did not find any candidate adaptive variants associated with pigmentation in Chichancanab; instead, we identified adaptive variants associated with four spermatogenesis genes, *ERC2*, *TBC1D25*, *C4orf22*, and *STK35*. Consistent with previous work demonstrating the importance of these genes in spermatogenesis (57–60), we found that *C. simus* does indeed have larger sperm heads than *C. beltrani*, suggesting a unique role for post-mating prezygotic reproductive barriers in this radiation (Figure 5D).

### Parallelism in the stages of adaptation: refinement with de novo and nonsynonymous mutations

Adaptation may occur in stages, with less pleiotropic cis-regulatory changes occurring before potentially more pleiotropic amino acid changes (26, 90, 91). Selection is also predicted to act on standing variation first (14, 92). In both radiations, we observed strong evidence for a refinement stage in which coding substitutions were selected on last. In Chichancanab, these late-sweeping coding substitutions were also de novo mutations (Figure 4). The simplest explanation for parallelism in more recent ages of adaptive de novo mutations is the waiting time required for beneficial mutations to (1) arise and (2) reach a substantial frequency within the population (14, 92). These variants may only be selected in the later stages of an adaptive walk because nonsynonymous mutations are often pleiotropic and likely deleterious in the ancestral genetic background (14, 93). Thus, they may not be advantageous until later in the adaptive walk, when sign epistasis may reverse negative pleiotropic effects (94–98). Our results highlight that this final refinement stage is likely a general pattern during adaptive divergence.

## Materials and Methods

### Sampling and genotyping

In 2022, we collected Lake Chichancanab specimens (*C. labiosus*, *C. beltrani*, and *C. simus*) and *C. artifrons* (Figure 1C). Additional species were collected from the wild or sourced from captive breeding populations in previous years. All specimens were euthanized in an overdose of buffered MS-222 (Fiquel, Inc.) following approved animal care and use protocols from the University of California, Berkeley and the University of California, Davis. All specimens used in this study are catalogued in the Museum of Vertebrate Zoology Fishes collection (MVZ:Fish:1410–1499).

Individual DNA samples were sequenced using Illumina HiSeq 4000 and NovaSeq. We mapped reads to the UCB_CyNevMio_1.0 *Cyprindon nevadensis mionectes* reference genome (GCA_030533455.1). Variants were called using *GATK* (v.4.5.0.0) and filtered according to best practices (99), resulting in 23,960,536 SNPs. See SI for more details.

### Population structure and demographic history of Yucatán Cyprinodon pupfishes

To assess population structure, we first pruned SNPs in linkage disequilibrium using *PLINK* (v. 1.9), resulting in 5,476,855 SNPs. With this linkage disequilibrium pruned data set, we visualized population structure with a PCA (*PLINK*) and *ADMIXTURE* (v1.3) (100).

We estimated demographic history using *MSMC2* (101) using three individuals with the highest mean depth. See SI for more details.

### Identifying and characterizing candidate genes

We calculated *F*_*st*_ genome-wide, per site, and in non-overlapping 10-kb windows in *VCFtools* (v0.1.16) for all pairwise combinations of Yucatán *Cyprinodon* species (102). With the 10-kb windowed *F*_*st*_ values, we calculated a modified, normalized population branch statistic (PBS), *PBS*_*nj*_ (103). We classified SNPs as candidate adaptive variants if they were fixed or nearly fixed (per-site *F*_*st*_ >0.95) compared to other species within the radiation and were among the top 1% outliers out of all *PBS*_*nj*_ windows, following the threshold used in (41).

We classified variants within 20 kb of a gene (upstream or downstream) as flanking. For variants within a gene, we further classified them into intronic, synonymous, or nonsynonymous using *SnpEff* (104). To characterize the origin of candidate variants, we extracted the alleles from every species for each candidate variant. We classified variants that were found in any other populations outside of Lake Chichancanab as standing genetic variation. We further subdivided this group into variants that were found in other species but not in *C. artifrons*. Finally, if the alternative variant was only detected within Chichancanab species, we classified it as de novo. See SI for more details.

### Introgression

We tested for evidence of introgression in Yucatán *Cyprinodon* species using *Dsuite* with *Cualac tessellatus* as the outgroup, calculating *f*_*4*_-ratios, *D* statistics, and *f*-branch statistics (49). For species trios that showed evidence of introgression where *C. simus* was either P1or P2, we then looked for introgression regions across the genome by calculating the distance fraction (*d*_*f*_ (50)), in non-overlapping windows of 91 informative SNPs. To estimate introgression block sizes, we merged neighboring windows that were introgression outliers and summed the total size of the adjacent block. See SI for more details.

### Timing of selective sweeps

For each candidate gene, we used *starTMRCA* (51) to estimate the age of selective sweeps, extracting a 1-Mb region surrounding the candidate variant (51). We used a fixed recombination rate of 2×10^−8^ (swordtail fishes; (105)) and a mutation rate of 1.56 × 10^−8^ substitutions per base pair (Caribbean *Cyprinodon* species; 26). We then conducted separate generalized linear models to test if the sweep age was correlated with the proportion of nonsynonymous SNPs, proportion of de novo SNPs, and the number of SNPs. See SI for more details.

### Optomotor response, sperm morphology, and Wnt inhibition experiment

We tested for differences in visual acuity between the generalist/detritivore, *C. beltrani*, and the zooplanktivore, *C. simus*, by conducting an optomotor response behavioral assay (56) on lab-reared *C. beltrani* (F1; *n* = 7) and lab-reared *C. simus* (F10+; *n* = 5). Because the data were nonparametric, we used a Wilcoxon rank-sum test to assess differences in optomotor response.

To test for differences in sperm morphology, we collected sperm samples from five individuals per species in the lab (F10+*C. simus,* F1 *C. artifrons,* F1 *C. beltrani*). After fixing the sperm sample in a 4% PFA solution stained with Rose Bengal, we plated and imaged sperm under oil immersion with a 63x objective. We measured 10–30 sperm per individual in ImageJ (106). We fit separate linear mixed-effects models for each trait of interest (sperm head, midpiece, and flagellum lengths) with species as a fixed effect and a random intercept for ndividual ID.

To confirm the role of the Wnt pathway in proper jaw and teeth development, we conducted an experiment chemically inhibiting the Wnt pathway using iCRT14 during metamorphosis (8dpf to 22 dpf) in *C. beltrani*. To control the exact time of development, we first performed in vitro fertilizations and incubated developing embryos at ~26 °C until hatching (~8dpf). On the day of hatching, we haphazardly split broods into control (DMSO) and experimental groups (100 nanomolar iCRT14) to control for batch and family effects. After euthanizing and staining fish with alizarin red (bone) and alcian blue (cartilage; (107)), we quantified the number of ossified teeth and measured the tooth length and base width for the left and right teeth closest to the mandibular symphysis using ImageJ (106). We fit separate linear mixed-effects models for average tooth length and base width with experimental treatment as a fixed effect and a random intercept for brood/replicate. For the number of ossified teeth, we used a generalized linear mixed-effect model with a Poisson family and log-link function. See SI for more details.

## Supplementary Material

Supplement 1

## References

[1] Gillespie R. G., Comparing adaptive radiations across space, time, and taxa. J. Hered. 111, 1–20 (2020). 31958131 10.1093/jhered/esz064 PMC7931853

[2] Martin C. H., Richards E. J., The paradox behind the pattern of rapid adaptive radiation: how can the speciation process sustain itself through an early burst? Annu. Rev. Ecol. Evol. Syst. 50, 569–593 (2019). 36237480 10.1146/annurev-ecolsys-110617-062443 PMC9555815

[3] Naciri Y., Linder H. P., The genetics of evolutionary radiations. Biol. Rev. 95, 1055–1072 (2020). 32233014 10.1111/brv.12598

[4] Schluter D., The ecology of adaptive radiation (OUP Oxford, 2000).

[5] Stroud J. T., Losos J. B., Ecological opportunity and adaptive radiation. Annu. Rev. of Ecol. Evol. Syst. 47, 507–532 (2016).

[6] Butlin R., What do we need to know about speciation? Trends Ecol. Evol. 27, 27–39 (2012). 21978464 10.1016/j.tree.2011.09.002

[7] Coyne J. A., Orr H. A., Speciation (Oxford University Press, 2004).

[8] Matute D. R., Cooper B. S., Comparative studies on speciation: 30 years since Coyne and Orr. Evolution 75, 764–778 (2021). 33491225 10.1111/evo.14181 PMC8247902

[9] Schluter D., Rieseberg L. H., Three problems in the genetics of speciation by selection. Proc. Natl. Acad. Sci. U.S.A. 119, e2122153119 (2022). 35858397 10.1073/pnas.2122153119 PMC9335311

[10] Berner D., Salzburger W., The genomics of organismal diversification illuminated by adaptive radiations. Trends Genet. 31, 491–499 (2015). 26259669 10.1016/j.tig.2015.07.002

[11] Marques D. A., Meier J. I., Seehausen O., A combinatorial view on speciation and adaptive radiation. Trends Ecol. Evol. 34, 531–544 (2019). 30885412 10.1016/j.tree.2019.02.008

[12] Pease J. B., Haak D. C., Hahn M. W., Moyle L. C., Phylogenomics reveals three sources of adaptive variation during a rapid radiation. PLOS Biol. 14, e1002379 (2016). 26871574 10.1371/journal.pbio.1002379 PMC4752443

[13] Stone B. W., Wessinger C. A., Ecological diversification in an adaptive radiation of plants: The role of de novo mutation and introgression. Mol. Biol. Evol. 41, msae007 (2024). 38232726 10.1093/molbev/msae007 PMC10826641

[14] Barrett R. D. H., Schluter D., Adaptation from standing genetic variation. Trends Ecol. Evol. 23, 38–44 (2008). 18006185 10.1016/j.tree.2007.09.008

[15] Rousselle M., Is adaptation limited by mutation? A timescale-dependent effect of genetic diversity on the adaptive substitution rate in animals. PLOS Genet. 16, e1008668 (2020). 32251427 10.1371/journal.pgen.1008668 PMC7162527

[16] Seehausen O., Hybridization and adaptive radiation. Trends Ecol. Evol. 19, 198–207 (2004). 16701254 10.1016/j.tree.2004.01.003

[17] Kagawa K., Takimoto G., Seehausen O., Transgressive segregation in mating traits drives hybrid speciation. Evolution 77, 1622–1633 (2023). 37094817 10.1093/evolut/qpad072

[18] Kagawa K., Takimoto G., Hybridization can promote adaptive radiation by means of transgressive segregation. Ecol. Lett. 21, 264–274 (2018). 29243294 10.1111/ele.12891

[19] De-Kayne R., Genomic architecture of adaptive radiation and hybridization in Alpine whitefish. Nat. Commun. 13, 4479 (2022). 35918341 10.1038/s41467-022-32181-8 PMC9345977

[20] Joyce D. A., Repeated colonization and hybridization in Lake Malawi cichlids. Curr. Biol. 21, R108–R109 (2011). 21300271 10.1016/j.cub.2010.11.029

[21] Lamichhaney S., Evolution of Darwin’s finches and their beaks revealed by genome sequencing. Nature 518, 371–375 (2015). 25686609 10.1038/nature14181

[22] Martin C. H., Complex histories of repeated gene flow in Cameroon crater lake cichlids cast doubt on one of the clearest examples of sympatric speciation. Evolution 69, 1406–1422 (2015). 25929355 10.1111/evo.12674

[23] Meier J. I., Ancient hybridization fuels rapid cichlid fish adaptive radiations. Nat. Commun. 8, 14363 (2017). 28186104 10.1038/ncomms14363 PMC5309898

[24] Pouchon C., Phylogenomic analysis of the explosive adaptive radiation of the Espeletia complex (Asteraceae) in the tropical Andes. Syst. Biol. 67, 1041–1060 (2018). 30339252 10.1093/sysbio/syy022

[25] Richards E. J., Poelstra J. W., Martin C. H., Don’t throw out the sympatric speciation with the crater lake water: fine-scale investigation of introgression provides equivocal support for causal role of secondary gene flow in one of the clearest examples of sympatric speciation. Evol. Lett. 2, 524–540 (2018). 30283699 10.1002/evl3.78 PMC6145409

[26] Richards E. J., A vertebrate adaptive radiation is assembled from an ancient and disjunct spatiotemporal landscape. Proc. Natl. Acad. Sci. U.S.A. 118, e2011811118 (2021). 33990463 10.1073/pnas.2011811118 PMC8157919

[27] Rosser N., Hybrid speciation driven by multilocus introgression of ecological traits. Nature 628, 811–817 (2024). 38632397 10.1038/s41586-024-07263-w PMC11041799

[28] John M. E. S., Dunker J. C., Richards E. J., Romero S., Martin C. H., Parallel evolution of integrated craniofacial traits in trophic specialist pupfishes. Ecol. Evol. 14, e11640 (2024). 38979003 10.1002/ece3.11640 PMC11228360

[29] Martin C. H., Wainwright P. C., Trophic novelty is linked to exceptional rates of morphological diversification in two adaptive radiations of Cyprinodon pupfish. Evolution 65, 2197–2212 (2011). 21790569 10.1111/j.1558-5646.2011.01294.x

[30] Patton A. H., Richards E. J., Gould K. J., Buie L. K., Martin C. H., Hybridization alters the shape of the genotypic fitness landscape, increasing access to novel fitness peaks during adaptive radiation. eLife 11, e72905 (2022). 35616528 10.7554/eLife.72905 PMC9135402

[31] Richards E. J., Martin C. H., Adaptive introgression from distant Caribbean islands contributed to the diversification of a microendemic adaptive radiation of trophic specialist pupfishes. PLOS Genet. 13, e1006919 (2017). 28796803 10.1371/journal.pgen.1006919 PMC5552031

[32] Richards E. J., Martin C. H., We get by with a little help from our friends: shared adaptive variation provides a bridge to novel ecological specialists during adaptive radiation. Proc. Biol. Sci. 289, 20220613 (2022). 35611537 10.1098/rspb.2022.0613 PMC9130792

[33] Humphries J. M., Miller R. R., A Remarkable Species Flock of Pupfishes, Genus Cyprinodon, from Yucatán, México. Copeia 1981, 52–64 (1981).

[34] Hodell D. A., Curtis J. H., Brenner M., Possible role of climate in the collapse of Classic Maya civilization. Nature 375, 391–394 (1995).

[35] Strecker U., Description of a new species from Laguna Chichancanab, Yucatan, Mexico: Cyprinodon suavium (Pisces: Cyprinodontidae). Hydrobiologia 541, 107–115 (2005).

[36] Humphries J. M., Cyprinodon verecundus, n. sp., a fifth species of pupfish from Laguna Chichancanab. Copeia 1984, 58–68 (1984).

[37] Gracida-Juárez C. A., Schmitter-Soto J. J., Genner M. J., Community structure of indigenous fishes relative to habitat variation and invasive tilapia in lakes of Quintana Roo, Mexico. Environ. Biol. Fish. 107, 401–414 (2024).

[38] Strecker U., The impact of invasive fish on an endemic Cyprinodon species flock (Teleostei) from Laguna Chichancanab, Yucatan, Mexico. Ecol. Freshw. Fish 15, 408–418 (2006).

[39] Strecker U., Genetic differentiation and reproductive isolation in a Cyprinodon fish species flock from Laguna Chichancanab, Mexico. Mol. Phyl. and Evol. 39, 865–872 (2006). 10.1016/j.ympev.2006.01.008 16483800

[40] Strecker U., Meyer C. G., Sturmbauer C., Wilkens H., Genetic divergence and speciation in an extremely young species flock in Mexico formed by the genus Cyprinodon (Cyprinodontidae, Teleostei). Mol. Phyl. Evol. 6, 143–149 (1996). 10.1006/mpev.1996.0066 8812314

[41] Shpak M., Lawrence K. N., Pool J. E., The precision and power of population branch statistics in identifying the genomic signatures of local adaptation. Genome Biol. Evol. 17, evaf080 (2025). 40326284 10.1093/gbe/evaf080 PMC12095133

[42] McGirr J. A., Martin C. H., Novel candidate genes underlying extreme trophic specialization in Caribbean pupfishes. Mol. Biol. Evol. 34, 873 (2017). 28028132 10.1093/molbev/msw286 PMC5850223

[43] McGirr J. A., Martin C. H., Parallel evolution of gene expression between trophic specialists despite divergent genotypes and morphologies. Evol. Lett. 2, 62–75 (2018). 30283665 10.1002/evl3.41 PMC6089502

[44] McGirr J. A., Martin C. H., Ecological divergence in sympatry causes gene misexpression in hybrids. Mol. Ecol. 29, 2707–2721 (2020). 32557903 10.1111/mec.15512 PMC8209238

[45] McGirr J. A., Martin C. H., Few fixed variants between trophic specialist pupfish species reveal candidate cis-regulatory alleles underlying rapid craniofacial divergence. Mol. Biol. Evol. 38, 405–423 (2021). 32877534 10.1093/molbev/msaa218 PMC7826174

[46] Palominos M. F., Muhl V., Martin C. H., Craniofacial-specific transcriptomics uncovers novel genes underlying jaw divergence in dietary specialist pupfishes. Genetics 231, iyaf207 (2025). 41001823 10.1093/genetics/iyaf207 PMC12693500

[47] Bragato C., Zebrafish dnm1a gene plays a role in the formation of axons and synapses in the nervous tissue. J. Neurosci. Res. 101, 1345–1359 (2023). 37031448 10.1002/jnr.25197

[48] Gonzaga-Jauregui C., Exome sequence analysis suggests that genetic burden contributes to phenotypic variability and complex neuropathy. Cell Rep. 12, 1169–1183 (2015). 26257172 10.1016/j.celrep.2015.07.023 PMC4545408

[49] Malinsky M., Matschiner M., Svardal H., Dsuite - Fast D-statistics and related admixture evidence from VCF files. Mol. Ecol. Res. 21, 584–595 (2021). 10.1111/1755-0998.13265 PMC7116594 33012121

[50] Pfeifer B., Kapan D. D., Estimates of introgression as a function of pairwise distances. BMC Bioinform. 20, 207 (2019). 10.1186/s12859-019-2747-z PMC6480520 31014244

[51] Smith J., Coop G., Stephens M., Novembre J., Estimating time to the common ancestor for a beneficial allele. Mol. Biol. Evol. 35, 1003–1017 (2018). 29361025 10.1093/molbev/msy006 PMC5888984

[52] Streelman J. T., Danley P. D., The stages of vertebrate evolutionary radiation. Trends Ecol. Evol. 18, 126–131 (2003).

[53] Li W.-G., Xu T.-L., ASIC3 Channels in multimodal sensory perception. ACS Chem. Neurosci. 2, 26–37 (2011). 22778854 10.1021/cn100094b PMC3369706

[54] Le T., A zebrafish model of crim1 loss of function has small and misshapen lenses with dysregulated clic4 and fgf1b expression. Front. Cell Dev. Biol. 13 (2025). 10.3389/fcell.2025.1522094 PMC11922885 40114969

[55] Kennedy B. N., Zebrafish rx3 and mab21l2 are required during eye morphogenesis. Dev. Biol. 270, 336–349 (2004). 15183718 10.1016/j.ydbio.2004.02.026

[56] Neuhauss S. C. F., Genetic disorders of vision revealed by a behavioral screen of 400 essential loci in zebrafish. J. Neurosci. 19, 8603–8615 (1999). 10493760 10.1523/JNEUROSCI.19-19-08603.1999 PMC6783047

[57] Chau M. H. K., Investigation of the genetic etiology in male infertility with apparently balanced chromosomal structural rearrangements by genome sequencing. Asian J. of Androl. 24 (2022). 10.4103/aja2021106 PMC9226698 35017386

[58] Li H., Dai Y., Luo Z., Nie D., Cloning of a new testis-enriched gene C4orf22 and its role in cell cycle and apoptosis in mouse spermatogenic cells. Mol. Biol. Rep. 46, 2029–2038 (2019). 30820741 10.1007/s11033-019-04651-8

[59] Miyamoto Y., The STK35 locus contributes to normal gametogenesis and encodes a lncRNA responsive to oxidative stress. Biol. Open 7, bio032631 (2018). 29970477 10.1242/bio.032631 PMC6124569

[60] Nawaz S., Hussain S., Basit S., Ahmad W., First evidence of involvement of TBC1D25 in causing human male infertility. Eur. J. Med. Genet. 64, 104142 (2021). 33460826 10.1016/j.ejmg.2021.104142

[61] Square T. A., Modulation of tooth regeneration through opposing responses to Wnt and BMP signals in teleosts. Development 150, dev202168 (2023). 38059590 10.1242/dev.202168 PMC10730089

[62] Nawaz S., WNT10A missense mutation associated with a complete odonto-onycho-dermal dysplasia syndrome. Eur. J. Hum. Genet. 17, 1600–1605 (2009). 19471313 10.1038/ejhg.2009.81 PMC2987016

[63] Benard E. L., WNT10A is required for zebrafish median fin fold maintenance and adult unpaired fin metamorphosis. Dev. Dyn. 253, 566–592 (2024). 37870737 10.1002/dvdy.672 PMC11035493

[64] Catrow J. L., Zhang Y., Zhang M., Ji H., Discovery of selective small-molecule inhibitors for the β-catenin/T-cell factor protein–protein interaction through the optimization of the acyl hydrazone moiety. J. Med. Chem. 58, 4678–4692 (2015). 25985283 10.1021/acs.jmedchem.5b00223

[65] Gonsalves F. C., An RNAi-based chemical genetic screen identifies three small-molecule inhibitors of the Wnt/wingless signaling pathway. Proc. Natl. Acad. Sci. U.S.A. 108, 5954–5963 (2011). 21393571 10.1073/pnas.1017496108 PMC3076864

[66] Enbody E. D., Community-wide genome sequencing reveals 30 years of Darwin’s finch evolution. Science 381, eadf6218 (2023). 37769091 10.1126/science.adf6218

[67] Peñalba J. V., The role of hybridization in species formation and persistence. Cold Spring Harb. Perspect. Biol. 16, a041445 (2024). 38438186 10.1101/cshperspect.a041445 PMC11610762

[68] Seehausen O., Conditions when hybridization might predispose populations for adaptive radiation. J. Evol. Biol. 26, 279–281 (2013). 23324007 10.1111/jeb.12026

[69] Schluter D., Adaptive radiation along genetic lines of least resistance. Evolution 50, 1766–1774 (1996). 28565589 10.1111/j.1558-5646.1996.tb03563.x

[70] Hornemann T., The SPTLC3 subunit of serine palmitoyltransferase generates short chain sphingoid bases. J. of Biol. Chem. 284, 26322–26330 (2009). 19648650 10.1074/jbc.M109.023192 PMC2785320

[71] Bronner M. E., LeDouarin N. M., Development and evolution of the neural crest: An overview. Dev. Biol. 366, 2–9 (2012). 22230617 10.1016/j.ydbio.2011.12.042 PMC3351559

[72] Hirth F., Reichert H., Conserved genetic programs in insect and mammalian brain development. BioEssays 21, 677–684 (1999). 10440864 10.1002/(SICI)1521-1878(199908)21:8<677::AID-BIES7>3.0.CO;2-8

[73] Reaume C. J., Sokolowski M. B., Conservation of gene function in behaviour. Philos. Trans. R. Soc. B. 366, 2100–2110 (2011). 10.1098/rstb.2011.0028 PMC3130371 21690128

[74] Abzhanov A., Protas M., Grant B. R., Grant P. R., Tabin C. J., Bmp4 and morphological variation of beaks in Darwin’s finches. Science 305, 1462–1465 (2004). 15353802 10.1126/science.1098095

[75] Albertson R. C., Streelman J. T., Kocher T. D., Yelick P. C., Integration and evolution of the cichlid mandible: The molecular basis of alternate feeding strategies. Proc. Natl. Acad. Sci. U.S.A. 102, 16287–16292 (2005). 16251275 10.1073/pnas.0506649102 PMC1283439

[76] Terai Y., Morikawa N., Okada N., The evolution of the pro-domain of bone morphogenetic protein 4 (Bmp4) in an explosively speciated lineage of East African cichlid fishes. Mol. Biol. Evol. 19, 1628–1632 (2002). 12200490 10.1093/oxfordjournals.molbev.a004225

[77] Carleton K. L., Escobar-Camacho D., Stieb S. M., Cortesi F., Marshall N. J., Seeing the rainbow: mechanisms underlying spectral sensitivity in teleost fishes. J. Exp. Biol. 223, jeb193334 (2020). 32327561 10.1242/jeb.193334 PMC7188444

[78] Hofmann C. M., The eyes have it: regulatory and structural changes both underlie cichlid visual pigment diversity. PLOS Biol. 7, e1000266 (2009). 20027211 10.1371/journal.pbio.1000266 PMC2790343

[79] O’Quin K. E., Hofmann C. M., Hofmann H. A., Carleton K. L., Parallel evolution of opsin gene expression in African cichlid fishes. Mol. Biol. Evol. 27, 2839–2854 (2010). 20601410 10.1093/molbev/msq171

[80] Ritchie M. G., Sexual selection and speciation. Annu. Rev. Ecol. Evol. Syst. 38, 79–102 (2007).

[81] Servedio M. R., Boughman J. W., The role of sexual selection in local adaptation and speciation. Annu. Rev. Ecol. Evol. Syst. 48, 85–109 (2017).

[82] Devigili A., Possible glimpses into early speciation: the effect of ovarian fluid on sperm velocity accords with post-copulatory isolation between two guppy populations. J. Evol. Biol. 31, 66–74 (2018). 29044818 10.1111/jeb.13194

[83] Garlovsky M. D., Synthesis and scope of the role of postmating prezygotic isolation in speciation. Cold Spring Harb. Perspect. Biol. 16, a041429 (2023). 10.1101/cshperspect.a041429 PMC11444258 38151330

[84] Kustra M. C., Servedio M. R., Alonzo S. H., Cryptic female choice can maintain reproductive isolation. Evolution 79, 2259–2273 (2025). 40728922 10.1093/evolut/qpaf156

[85] Kustra M. C., Alonzo S. H., The coevolutionary dynamics of cryptic female choice. Evol. Lett. 7, 191–202 (2023). 37475752 10.1093/evlett/qrad025 PMC10355280

[86] Lorch P. D., Servedio M. R., The evolution of conspecific gamete precedence and its effect on reinforcement. J. Evol. Biol. 20, 937–949 (2007). 17465905 10.1111/j.1420-9101.2007.01306.x

[87] Marshall J. L., Arnold M. L., Howard D. J., Reinforcement: the road not taken. Trends Ecol. Evol. 17, 558–563 (2002).

[88] Kodric-Brown A., West R. J. D., Asymmetries in premating isolating mechanisms in a sympatric species flock of pupfish (Cyprinodon). Behav. Ecol. 25, 69–75 (2014).

[89] Strecker U., Kodric-Brown A., Mate recognition systems in a species flock of Mexican pupfish. Journal of Evolutionary Biology 12, 927–935 (1999).

[90] Campagna L., Repeated divergent selection on pigmentation genes in a rapid finch radiation. Science Advances 3, e1602404 (2017). 28560331 10.1126/sciadv.1602404 PMC5443641

[91] Jones F. C., The genomic basis of adaptive evolution in threespine sticklebacks. Nature 484, 55–61 (2012). 22481358 10.1038/nature10944 PMC3322419

[92] Blount Z. D., Lenski R. E., Losos J. B., Contingency and determinism in evolution: Replaying life’s tape. Science 362, eaam5979 (2018). 30409860 10.1126/science.aam5979

[93] Hedrick P. W., Adaptive introgression in animals: examples and comparison to new mutation and standing variation as sources of adaptive variation. Mol. Ecol. 22, 4606–4618 (2013). 23906376 10.1111/mec.12415

[94] Das S. G., Mungan M., Krug J., Epistasis-mediated compensatory evolution in a fitness landscape with adaptational tradeoffs. Proc. Natl. Acad. Sci. U.S.A. 122, e2422520122 (2025). 40215274 10.1073/pnas.2422520122 PMC12012525

[95] Harms M. J., Thornton J. W., Historical contingency and its biophysical basis in glucocorticoid receptor evolution. Nature 512, 203–207 (2014). 24930765 10.1038/nature13410 PMC4447330

[96] Karageorgi M., Genome editing retraces the evolution of toxin resistance in the monarch butterfly. Nature 574, 409–412 (2019). 31578524 10.1038/s41586-019-1610-8 PMC7039281

[97] Tarvin R. D., Interacting amino acid replacements allow poison frogs to evolve epibatidine resistance. Science 357, 1261–1266 (2017). 28935799 10.1126/science.aan5061 PMC5834227

[98] Weinreich D. M., Delaney N. F., DePristo M. A., Hartl D. L., Darwinian evolution can follow only very few mutational paths to fitter proteins. Science 312, 111–114 (2006). 16601193 10.1126/science.1123539

[99] McKenna A., The genome analysis toolkit: A MapReduce framework for analyzing next-generation DNA sequencing data. Genome Res. 20, 1297–1303 (2010). 20644199 10.1101/gr.107524.110 PMC2928508

[100] Alexander D. H., Novembre J., Lange K., Fast model-based estimation of ancestry in unrelated individuals. Genome Res. 19, 1655–1664 (2009). 19648217 10.1101/gr.094052.109 PMC2752134

[101] Schiffels S., Wang K., “MSMC and MSMC2: The multiple sequentially markovian coalescent” in Statistical Population Genomics, Dutheil J. Y., Ed. (Springer US, 2020), pp. 147–166. 10.1007/978-1-0716-0199-0_7 31975167

[102] Danecek P., The variant call format and VCFtools. Bioinformatics 27, 2156–2158 (2011). 21653522 10.1093/bioinformatics/btr330 PMC3137218

[103] Schmidt J. M., de Manuel M., Marques-Bonet T., Castellano S., Andrés A. M., The impact of genetic adaptation on chimpanzee subspecies differentiation. PLOS Genet. 15, e1008485 (2019). 31765391 10.1371/journal.pgen.1008485 PMC6901233

[104] Cingolani P., A program for annotating and predicting the effects of single nucleotide polymorphisms, SnpEff: SNPs in the genome of Drosophila melanogaster strain w1118; iso-2; iso-3. Fly 6, 80–92 (2012). 22728672 10.4161/fly.19695 PMC3679285

[105] Schumer M., Natural selection interacts with recombination to shape the evolution of hybrid genomes. Science 360, 656–660 (2018). 29674434 10.1126/science.aar3684 PMC6069607

[106] Rueden C. T., ImageJ2: ImageJ for the next generation of scientific image data. BMC Bioinform. 18, 529 (2017). 10.1186/s12859-017-1934-z PMC5708080 29187165

[107] Walker M., Kimmel C., A two-color acid-free cartilage and bone stain for zebrafish larvae. Biotech. Histochem. 82, 23–28 (2007). 17510811 10.1080/10520290701333558

