# Supplementary material for: Parallel and non-parallel features of adaptive radiation in Yucatán pupfishes
Source: bioRxiv. 2025 Dec 27:2025.11.17.688971. Originally published 2025 Nov 18. Preprint. [Version 3] doi: 10.1101/2025.11.17.688971 (PMC12667942; doi:10.1101/2025.11.17.688971)
Supplement: Supplement 1 [file NIHPP2025.11.17.688971v3-supplement-1.pdf]

bioRxiv preprint doi: https://doi.org/10.1101/2025.11.17.688971; this version posted December 27, 2025. The copyright holder for this preprint (which was not certified by peer review) is the author/funder, who has granted bioRxiv a license to display the preprint in perpetuity. It is made available under aCC-BY 4.0 International license.

Supporting Information for

Parallel and non-parallel features of adaptive radiation in Yucatán pupfishes

Matthew C. Kustra [*], David Tian, M. Fernanda Palominos, Feifei Guo, Dylan Chau, Oskar Golwala, HoWan Chan, Andrés Alvarez Zapata, Reyna Guadalupe Cetz Paredes, Frida Ximena Cortés Sánchez, Sonia Gabriela Hernández, Adan Fernando Mar-Silva, Fernando Mex, Charles Tralka, Maribel Badillo-Alemán, Juan J. Schmitter-Soto, Carlos A. Gracida-Juárez, Christopher M. Martinez, Jairo Arroyave, and Christopher H. Martin

[*]**Corresponding author:** Matthew Kustra

Email: matthewckustra@berkeley.edu

The supporting information includes:

Supporting text (SI methods)

Tables S1 to S4

Figures S1 to S10

SI References

bioRxiv preprint doi: https://doi.org/10.1101/2025.11.17.688971; this version posted December 27, 2025. The copyright holder for this preprint (which was not certified by peer review) is the author/funder, who has granted bioRxiv a license to display the preprint in perpetuity. It is made available under aCC-BY 4.0 International license.

SI Methods

Sampling

We collected Lake Chichancanab specimens in 2022 over two days using a 5 x 1.3 m seine net with 1.6 mm mesh size. We sampled two sites, an entrance road by La Presumida, and a bridge crossing the narrow section of the lake on the road to San Diego (Fig. 1C). *C. labiosus* and *C. beltrani* were sampled from both sites for this study, whereas *C. simus* was only found at La Presumida. *C. artifrons* were collected by cast net from a coastal estuary at Sisal. Additional specimens were collected from the Bahamas (Lake Cunningham, New Providence Island and Crescent Pond, San Salvador Island), the Dominican Republic (Laguna Bavaro), or Fort Fisher, North Carolina as described previously (Martin 2016) or sourced from the American Killifish Association, the London Zoological Society, the Dallas Children's Aquarium (*Megupsilon aporus*), and Frans Vermuelen (*Cyprinodon dearborni*). All specimens were euthanized in an overdose of buffered MS-222 (Fiquel, Inc.) following approved animal care and use protocols from the University of California, Berkeley and the University of California, Davis. All specimens used in this study are catalogued in the Museum of Vertebrate Zoology Fishes collection (MVZ:Fish:1410-1499).

The London Zoological Society provided our live colony of *C. simus* in 2015, originating from a much earlier field collection, which we subsequently raised for ten generations. We collected live colonies of *C. beltrani* and *C. artifrons* in 2024 from La Presumida, Lake Chichancanab and Sisal, respectively, over four days using a 5 x 1.3 m seine net with 1.6 mm mesh size.

Sequencing, Variant calling and filtering

bioRxiv preprint doi: https://doi.org/10.1101/2025.11.17.688971; this version posted December 27, 2025. The copyright holder for this preprint (which was not certified by peer review) is the author/funder, who has granted bioRxiv a license to display the preprint in perpetuity. It is made available under aCC-BY 4.0 International license.

Individual DNA samples were resequenced using Illumina Hiseq 4000 and Novaseq. Raw reads were trimmed with *fastp* (v0.23.4) (1). Reads were mapped to the UCB_CyNevMio_1.0 *Cyprindon nevadensis mionectes* reference genome (GCA_030533455.1) with *bwa mem* (v0.7.17) (2). We followed the *GATK* (v.4.5.0.0) genotyping pipeline to call variants (119). Duplicate reads in the bam files were marked with *Picard MarkDuplicates* (GATK v4.5.0.0) (118). Coverage and mapping quality were assessed with *qualimap* (v2.2.2) (4). We used *HaplotypeCaller* (-ERC GVCF) v4.5.0.0 to call variants for each individual and stored variants in the GenomicsDB datastore format prior to using *GenotypeGVCFs* to perform joint genotyping. We restricted our analyses to biallelic SNPs and applied the recommended *GATK* hard filters (QD < 2, QUAL < 30, SOR > 3, FS > 60, MQ < 40, MQRankSum < -12.5, ReadPosRankSum < -8) to filter SNPs (118, 120). Missing genotypes were reset to ./. from 0/0 due to how *GATK* represents missing genotypes in version 4.5.0.0 (7).

After hard-filtering and variant calling, we filtered the data set further by removing SNPs with minor allele frequency < 0.05, more than 10% missing, depth less than 10x, and GQ less than 20 using *BCFtools* (v1.16)(8). This resulted in a final data set that included 23,960,536 SNPs.

Population structure of Yucatán Cyprinodon *pupfishes*

To assess population structure, we first pruned SNPs in linkage disequilibrium using *PLINK* (v. 1.9) with the following parameters: "-indep-pairwise 50 5 0.2" (9). This filtered our data set from 23,960,536 SNPs to 5,476,855 SNPs. With this linkage disequilibrium pruned data set, we first filtered the full VCF file to only include relevant Yucatán *Cyprinodon* species (i.e., *C. artifrons, C. beltrani, C. labiosus,* and *C. simus*), then conducted a PCA using *PLINK*. Next,

bioRxiv preprint doi: https://doi.org/10.1101/2025.11.17.688971; this version posted December 27, 2025. The copyright holder for this preprint (which was not certified by peer review) is the author/funder, who has granted bioRxiv a license to display the preprint in perpetuity. It is made available under aCC-BY 4.0 International license.

we used *ADMIXTURE* (v1.3) to determine the optimal number of population clusters that best fit the data and to assign individuals to their corresponding population clusters (10). We performed this analysis and calculated cross-validation error for the number of clusters K = 1-6. Models in ADMIXTURE with K=2-4 were equally supported (Figure S2A). However, given morphological distinctness and the results of the PCA, we present the results for when K = 4 (Figure 1D).

Demographic history of Yucatán Cyprinodon *pupfish*

We estimated demographic history using *MSMC2* (11). For each Yucatán pupfish species, we selected three individuals with the highest mean depth and generated single-sample VCF files, individual mask files, and "mapability" mask files. We then ran *MSMC2* with default parameters except we set phasing to "unphased" and "P_PAR=8*1+25*1+1*2+1*3." For plotting, we used a generation time of 1 year and a mutation rate of 1.56 x 10^{-8} substitutions per base pair, estimated from lab-reared pedigrees of Caribbean *Cyprinodon* species (12).

Identifying candidate genes

We calculated F_{st} genome-wide, per site, and in non-overlapping 10-kb windows using the "--weir-fist-pop" function in *VCFtools*(v0.1.16) for all pairwise combinations of Yucatán *Cyprinodon* species (13). We chose 10-kb windows because linkage disequilibrium substantially decayed at this distance (Figure S3), and these windows allowed us to quantify fine-scale genomic variation. With the 10-kb windowed F_{st} values, we calculated a modified population branch statistic (PBS) for four taxa, PBS_{nj} (14). This statistic is useful because it makes no assumptions about species topology or polarization (14). We then normalized PBS_{nj} by the total tree length, as normalized *PBS* is most effective and specific at identifying selective sweeps (15).

bioRxiv preprint doi: https://doi.org/10.1101/2025.11.17.688971; this version posted December 27, 2025. The copyright holder for this preprint (which was not certified by peer review) is the author/funder, who has granted bioRxiv a license to display the preprint in perpetuity. It is made available under aCC-BY 4.0 International license.

We classified SNPs as candidate adaptive variants if they were fixed or nearly fixed (per-site F_{st} >0.95) compared to other species within the radiation and were among the top 1% outliers out of all PBS_{nj} windows, following the threshold used in (15).

Introgression

Using the full VCF file, which contained a wide range of species in the family Cyprinodontidae, we tested for evidence of introgression in Yucatán *Cyprinodon* species using *Dsuite* (16). We first excluded any samples/species that had an average depth < 4. We then calculated all possible trios with a species tree based on the most recent phylogeny (17) with *Cualac tessellatus* as the outgroup. We calculated Z-scores to assess the significance of *D* statistics using 1,000 jackknife blocks and considered a Z-score greater than three as evidence for significant introgression (18). We only focused our primary analysis on species trios where P1-P2 included species within the Lake Chichancanab radiation. We used the other trio calculations to calculate *f*-branch (f_b) statistics to account for correlated f_4-ratio scores and to inform the timing of introgression events (16).

For species trios that showed evidence of introgression where *C. simus* was either P1or P2, we then calculated the distance fraction (d_f), a robust metric of introgression for windowed analyses (19), in non-overlapping windows of 91 informative SNPs. We chose this number of SNPs because it resulted in windows of approximately 10 kb. We considered windows to be introgressed if they were in the top X% of absolute d_f value, where X% was the percentage of estimated genome wide introgression (e.g, f_4-ratio) for that focal group, following (20, 21). To estimate introgression block sizes, we merged neighboring windows that were introgression outliers and summed the total size of the adjacent blocks.

bioRxiv preprint doi: https://doi.org/10.1101/2025.11.17.688971; this version posted December 27, 2025. The copyright holder for this preprint (which was not certified by peer review) is the author/funder, who has granted bioRxiv a license to display the preprint in perpetuity. It is made available under aCC-BY 4.0 International license.

Characterizing candidate genes

After identifying candidate variants, we then used *Bedtools* (v2.31) to see if variants occurred near a gene (within 20 kb) or within a gene (22) with a GFF annotation file for the *C. nev. mionectes* reference genome (Tian et al. in prep). We classified variants within 20 kb of a gene (upstream or downstream) as flanking. For SNPs within a gene, we further classified them into intronic, synonymous, or nonsynonymous using *SnpEff* (23).

To characterize the origin of candidate variants, we extracted the alleles from every species for each candidate variant. We classified variants that were found in any other populations outside of Lake Chichancanab as standing genetic variation. We further subdivided this group into variants that were found in other species but not in *C. artifrons*. If variants fell within an introgression window, we classified them as “introgressed.” Finally, if the alternative variant was only detected within Chichancanab species, we classified it as de novo.

Timing of selective sweeps

To gain insight into the timing of selective sweeps in *C. simus*, we used *starTMRCA* (24). Because many genes contained multiple candidate variants, for this analysis, we selected the variant that was in the median position of each gene. We then extracted a 1-Mb region surrounding that variant and removed all sites that contained missing data. For simplification, we only used the generalist/detritivore, *C. beltrani*, as the “reference” population and the zooplanktivore, *C. simus*, as the “selected” population. We used a fixed recombination rate of $2 x 10^{-8}$ (swordtail fishes; (25)) and a mutation rate of 1.56×10^{-8} substitutions per base pair (Caribbean *Cyprinodon* species; 26). We then ran five separate Markov chains for 30,000

bioRxiv preprint doi: https://doi.org/10.1101/2025.11.17.688971; this version posted December 27, 2025. The copyright holder for this preprint (which was not certified by peer review) is the author/funder, who has granted bioRxiv a license to display the preprint in perpetuity. It is made available under aCC-BY 4.0 International license.

iterations with a proposal standard deviation of 150 (preliminary analyses changing this parameter had minimal influence on results). We then discarded the first 10,000 iterations of each chain as burn-in.

To test for stages of adaptation, we conducted separate generalized linear models to test if the sweep age was correlated with the proportion of nonsynonymous SNPs, proportion of de novo SNPs, and the number of SNPs. For the generalized linear regressions of proportion of nonsynonymous SNPs and proportion of de novo SNPs, we used a quasi-binomial family to account for overdispersion with a logit link function. For the number of SNPs, we used a quasi-Poisson family with a log link function. To test for the significance of sweep age, we used a likelihood ratio test comparing a model with the estimated sweep age as an effect compared to a null model.

Optomotor response

We tested for differences in visual acuity between the generalist/detritivore, *C. beltrani*, and the zooplanktivore, *C. simus*, by conducting an optomotor response behavioral assay (26). We tested lab-reared *C. beltrani* (F1, $n = 7$) and lab-reared *C. simus* (long-term laboratory colony exceeding ten generations in the lab, $n = 5$). Each fish was tested consecutively with each control or treatment for 1-minute observation periods, with the control observation occurring first, followed by presentation of all-black (positive control) and black-and-white spinning bars (treatment: acuity trial). The order of presentation for positive control and treatment was alternated in each trial. Fish were placed in a suspended cylindrical plastic container 0.3 m in diameter that remained stationary during the course of the trials. Spinning black-and-white or all-

bioRxiv preprint doi: https://doi.org/10.1101/2025.11.17.688971; this version posted December 27, 2025. The copyright holder for this preprint (which was not certified by peer review) is the author/funder, who has granted bioRxiv a license to display the preprint in perpetuity. It is made available under aCC-BY 4.0 International license.

black paper was rotated outside the clear plastic container at ~90 RPM during each observation period.

Due to the data being nonparametric (count, zero-inflated, unequal variance between groups), we conducted a Wilcoxon rank sum test for the on-banded (all black) and on-banded portions separately. Because we had an a priori hypothesis that *C. simus* would display a stronger optomotor response (greater visual acuity), we calculated *p*-values with a one-tailed test.

Sperm morphology

To test for differences in sperm morphology, we collected sperm samples from five individuals per species in the lab (*C. simus, C. artifrons, C. beltrani*). We first anesthetized the fish with a solution of MS-222. After the fish was anesthetized, we collected 1µL of milt (fish semen) with a 1µL glass microcapillary tube and fixed the sample overnight in a 4% PFA solution stained with Rose Bengal. We then plated sperm on a positively charged slide with a sealed coverslip. We imaged sperm with oil immersion with a 63x objective and measured 10-30 sperm per individual in ImageJ (27). Due to poor sperm samples from some individuals (not sufficient quantities), we ended up with the following sample sizes: three *simus*, four *artifrons*, and five *beltrani*.

To retain information about variation within individuals, we fit separate linear mixed-effects models for each trait of interest (sperm head, midpiece, and flagellum lengths) with population as a fixed effect and a random intercept for individual ID, using *LME4* (28). To assess the significance of the model, we used a Type II ANOVA with a Wald *F* test using Kenward-Roger degrees of freedom. If there was a significant effect of population, we conducted post hoc pairwise tests using the *Emmeans* package (29) and corrected for multiple comparisons using the

bioRxiv preprint doi: https://doi.org/10.1101/2025.11.17.688971; this version posted December 27, 2025. The copyright holder for this preprint (which was not certified by peer review) is the author/funder, who has granted bioRxiv a license to display the preprint in perpetuity. It is made available under aCC-BY 4.0 International license.

Tukey method. We did not correct for phylogeny due to how closely related the species are, the limited sample size, and the fact that the only significant differences were between sister species.

WNT experiment

To confirm the role of the WNT pathway in proper jaw and teeth development, we conducted an experiment chemically inhibiting the WNT pathway using iCRT14 during metamorphosis in *C. beltrani*. To control the exact time of development, we first performed in vitro fertilizations and incubated developing embryos at ~26 until hatching (~8dpf). On the day of hatching, we haphazardly split broods into control and experimental groups to control for batch and family effects. We raised experimental groups in tank water with a 100 nanomolar concentration of iCRT14 and control groups in a 100 nanomolar concentration of DMSO (solution used to dilute stock iCRT14). We ran the experiment for two weeks until individuals were 22dpf, changing the solution every 3-4 days. We performed this experiment on four separate broods (~12 individuals/treatment). One brood was excluded due to improper development (no ossification of the spine) in the controls.

After the experiment, we euthanized fish with MS-222 and stained fish with alizarin red (bone) and alcian blue (cartilage) following a modified published protocol (30). After staining, we prepared whole mounts and imaged fish with fluorescent microscopy (Zeiss LSM880 FCS) to quantify ossification of the jaw and teeth (31). We then dissected out the lower jaw and prepared flat mounts to measure teeth morphology. Specifically, we measured the tooth length and base width for the left and right teeth closest to the mandibular symphysis using ImageJ (27). For statistical analyses, we took the mean of the left and right tooth length and base width.

bioRxiv preprint doi: https://doi.org/10.1101/2025.11.17.688971; this version posted December 27, 2025. The copyright holder for this preprint (which was not certified by peer review) is the author/funder, who has granted bioRxiv a license to display the preprint in perpetuity. It is made available under aCC-BY 4.0 International license.

We fit separate linear mixed-effects models for tooth length and base width with experimental treatment as a fixed effect and a random intercept for brood/replicate, using *LME4* (28). For the number of ossified teeth, we used a generalized linear mixed-effect model with a Poisson family and log-link function. To assess the significance of these models, we used a likelihood ratio test comparing it to a null model without treatment as a fixed effect.

SI Tables

Table S1. Mean genome-wide pairwise F_{ST} statistics within the Chichancanab radiation and with sister outgroup (*artifrons*).

	labiosus	***beltrani***	***artifrons***
simus	0.090	0.068	0.182
labiosus		0.032	0.184
beltrani			0.168

Table S2. F_{ST} statistics within the Chichancanab radiation.

**Comparison**	**F_{ST} = 1 (fixed)**	**1> $F_{ST} \geq$ 0.95 (nearly fixed)**	**0.95> $F_{ST} \geq$ 0.9**	**0.9> $F_{ST} \geq$0.8**	**Other**	**Mean F_{ST}**
simus vs beltrani + labiosus	19	1127	3304	20173	10359605	0.07
labiosus vs *beltrani + simus*	0	0	3	94	10384131	0.04
beltrani vs *simus + labiosus*	0	0	0	13	10384215	0.02

Table S3. Fixed SNPs between *C. simus* and *C. beltrani* + *C. labiosus*. Bold rows indicate SNPs within top 1% of PBS windows. Bolded genes only are genes that have regions that fall within the top 1% PBS percentile.

Position	Gene	Distance to gene	PBS percentile	Annotation
Chr 1: 5290600	**STK35**	12594	1.85	
Chr 1:5290602	**STK35**	12592	1.85	Fertility, eye development and oxidative stress (32).
Chr 1:5290604	**STK35**	12590	1.85	
Chr 2: 17498913	SLC17A8	Intron	1.24	Glutamate neurotransmitter, inner hair cell dysfunction
Chr 3: **11687002**	**AGO3**	**Intron**	**0.34**	**RNA regulator**
Chr 3: 32286614	RBMS3	14363	22.93	
Chr 3: 32286627	RBMS3	14350	22.93	Involved in chondrogenic craniofacial development of zebrafish (33).
Chr 3: 32286638	RBMS3	14339	22.93	
Chr 3: 32286614	RBMS3	14363	22.93	
Chr 11: 27324192	GRIA3	Intron	2.85	
Chr 11: 27336469	GRIA3	2772	3.18	Glutamate receptor
Chr 11: 27336472	GRIA3	2775	3.18	
Chr 11: 27336476	GRIA3	2779	3.18	
Chr 11: **28255536**	**NA**	**3026**	**0.98**	**NA**
Chr 11: **28255616**	**NA**	**3106**	**0.98**	**NA**
Chr 11: **28256343**	**NA**	**3833**	**0.98**	**NA**
Chr 11: **28257367**	**NA**	**4857**	**0.98**	**NA**
Chr 11: 28260047	NA	7537	2.87	NA
Chr 12: 13854139	**C4orf22**	11903	1.30	Spermatogenesis, cilia, flagellum (34)
Chr 24: **12693158**	**KDM6A**	**Intron**	**0.05**	**Kabuki syndrome (craniofacial development; 35)**

bioRxiv preprint doi: https://doi.org/10.1101/2025.11.17.688971; this version posted December 27, 2025. The copyright holder for this preprint (which was not certified by peer review) is the author/funder, who has granted bioRxiv a license to display the preprint in perpetuity. It is made available under aCC-BY 4.0 International license.

Table S4. Summary statistics of the distance between closest adaptive candidate SNPs within the same gene

Gene	Median Distance	Mean Distance	SD	
TM6SF2	7430	7430	NA	2
C19orf10	4789	4789	NA	2
KDM6A	2892.5	2892.5	2410.52702	3
KCNB2	1803	1738.75	1614.92299	5
UBE2T	1742	1742	NA	2
TMEM230	1734	3039	3599.52427	4
ABCA1	1037.5	1097.5	959.375665	5
HAPLN4	974	1608.21739	9927.05273	24
GRIN2A	904	1270.6667	4273.38008	4
DPH1	608	1224.5	1999.25934	7
WNT10A	402	1442.17857	3986.39626	29
OPN1LW	386.5	359.5	225.229513	5
NIPSNAP3A	253	1451.71429	2819.19065	8
GALNT15	246.5	675.166667	3156.86746	13
SWS2	246	1381.25	2423.03892	5
STK35	217	793.366667	1992.8543	31
CRIM1	201	47.829268	2176.05381	42
PDYN	187	326.371429	355.925561	36
ADCYAP1R1	186.5	833	1304.35501	11
DNM1	186.5	459.708333	677.261781	25
DNM1	186.5	459.708333	677.261781	25
KCNH8	131.5	584.366667	1030.65532	31
TOP1	124	332.375	6687.63393	17
AGO3	115	357.625	552.097284	9
GMEB2	112	319.490196	2485.05809	52
GPNMB	57	57	77.781746	3
IGFBP4	26	2254	3880.67997	4
FAM83D	23	2523.66667	4347.74658	4
EXOSC3	17	17	NA	2
C4orf22	15	15	9.899495	3
TMEM106B	7	11.4	10.358571	6
THOC3	6	5.833333	3.600926	7
MID1	1	1	NA	2

bioRxiv preprint doi: https://doi.org/10.1101/2025.11.17.688971; this version posted December 27, 2025. The copyright holder for this preprint (which was not certified by peer review) is the author/funder, who has granted bioRxiv a license to display the preprint in perpetuity. It is made available under aCC-BY 4.0 International license.

1166 **SI Figures**

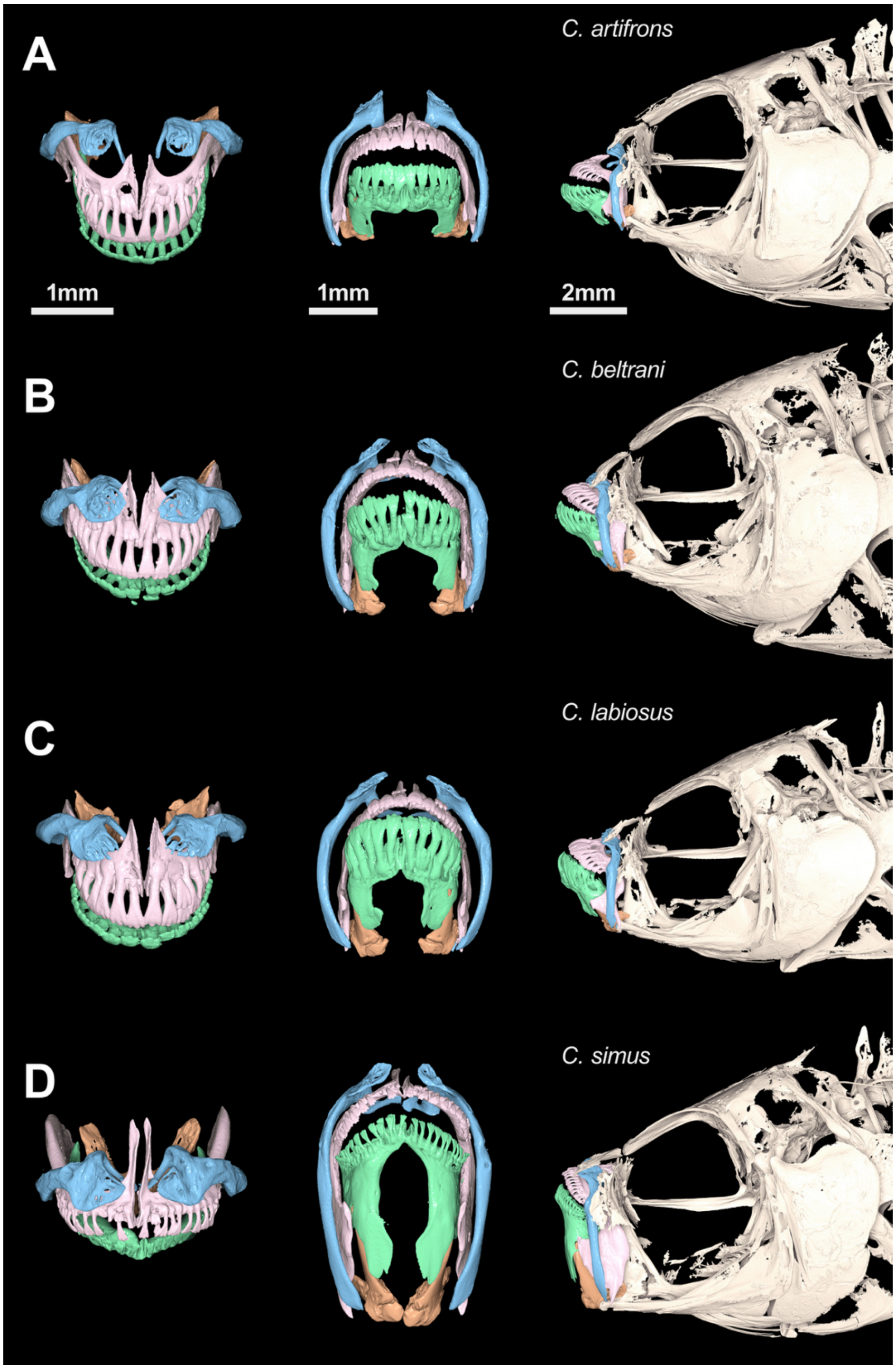

1167

1168 **Figure S1.** µCT scans of Yucatán pupfish with maxilla colored in blue, premaxilla in pink,

1169 dentary in green, and articular in orange.

bioRxiv preprint doi: https://doi.org/10.1101/2025.11.17.688971; this version posted December 27, 2025. The copyright holder for this preprint (which was not certified by peer review) is the author/funder, who has granted bioRxiv a license to display the preprint in perpetuity. It is made available under aCC-BY 4.0 International license.

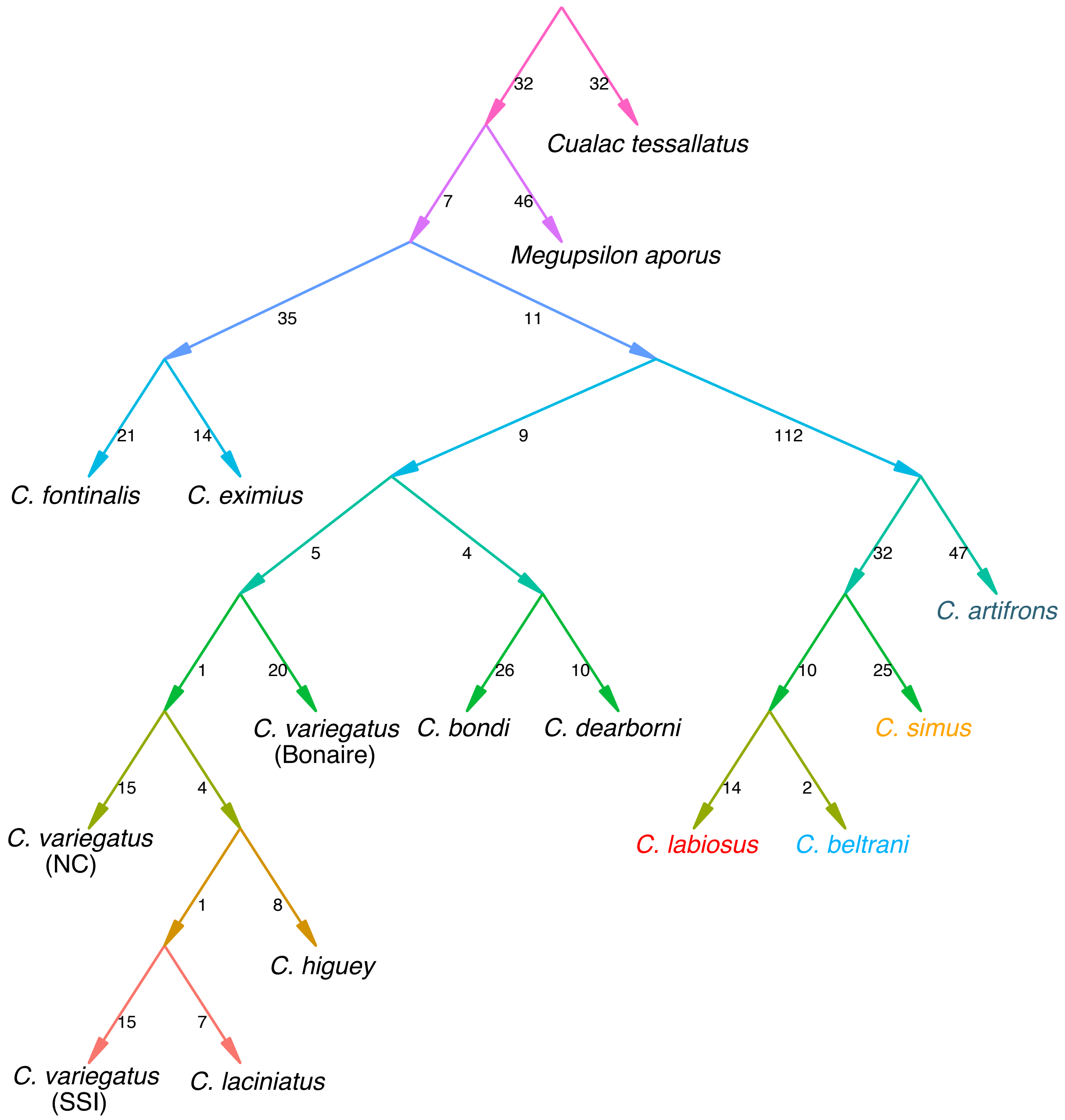

Figure S2. Best fitting admixture graph assuming zero admixture events. Admixture graph was found using the *findGraphs* function in *ADMIXTOOLS2* (36). Number on edges represent drift weights.

bioRxiv preprint doi: https://doi.org/10.1101/2025.11.17.688971; this version posted December 27, 2025. The copyright holder for this preprint (which was not certified by peer review) is the author/funder, who has granted bioRxiv a license to display the preprint in perpetuity. It is made available under aCC-BY 4.0 International license.

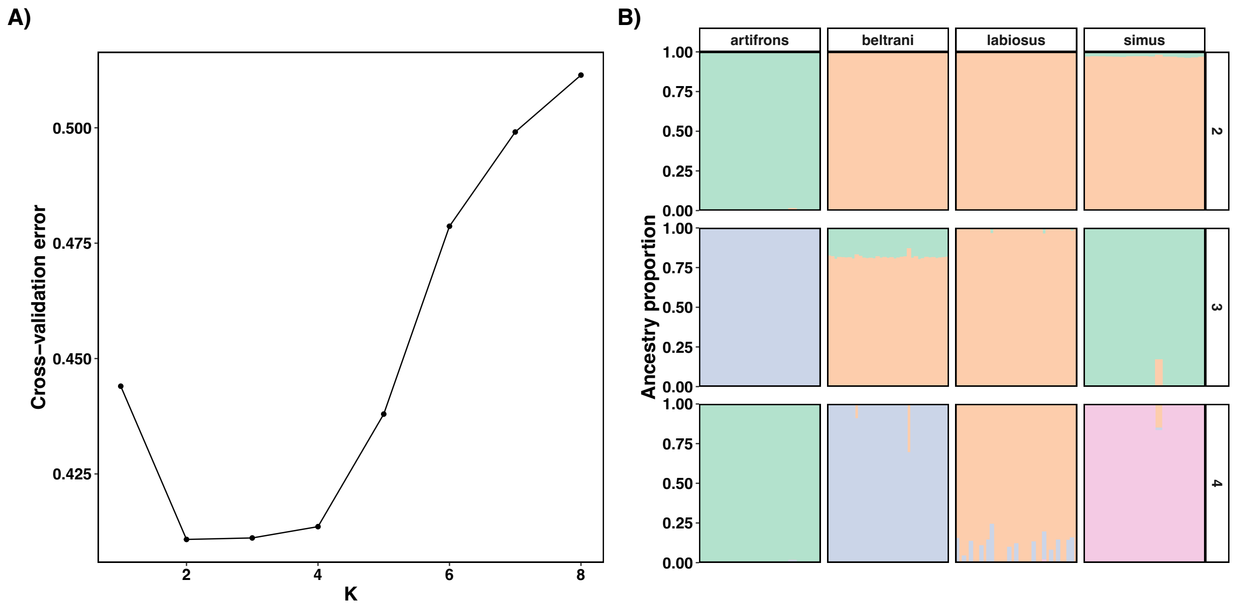

Figure S3. ADMIXTURE results support genetic differentiation of Yucatán Cyprinodon pupfish species. (A) Cross-validation error for different values of K clusters, equally support 2, 3, and 4 clusters. (B) *ADMIXTURE* plots showing the clusters at different values of K.

bioRxiv preprint doi: https://doi.org/10.1101/2025.11.17.688971; this version posted December 27, 2025. The copyright holder for this preprint (which was not certified by peer review) is the author/funder, who has granted bioRxiv a license to display the preprint in perpetuity. It is made available under aCC-BY 4.0 International license.

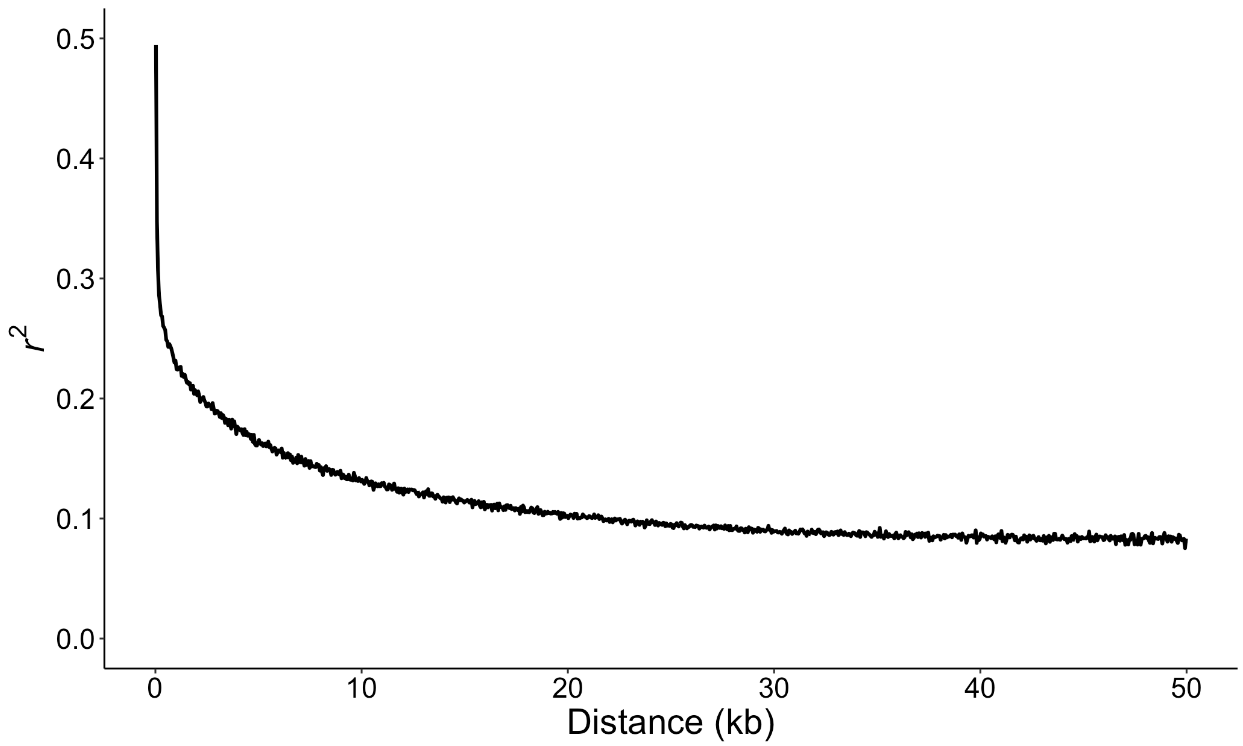

Figure S4. Linkage decay occurs rapidly in *Cyprinodon simus*. Linkage decay was calculated in windows of 1000 kb using *PLINK* (v1.9). The results graphed are a random sample of all calculated r^2 values taken for plotting purposes (n = 9,645 r^2 values).

bioRxiv preprint doi: https://doi.org/10.1101/2025.11.17.688971; this version posted December 27, 2025. The copyright holder for this preprint (which was not certified by peer review) is the author/funder, who has granted bioRxiv a license to display the preprint in perpetuity. It is made available under aCC-BY 4.0 International license.

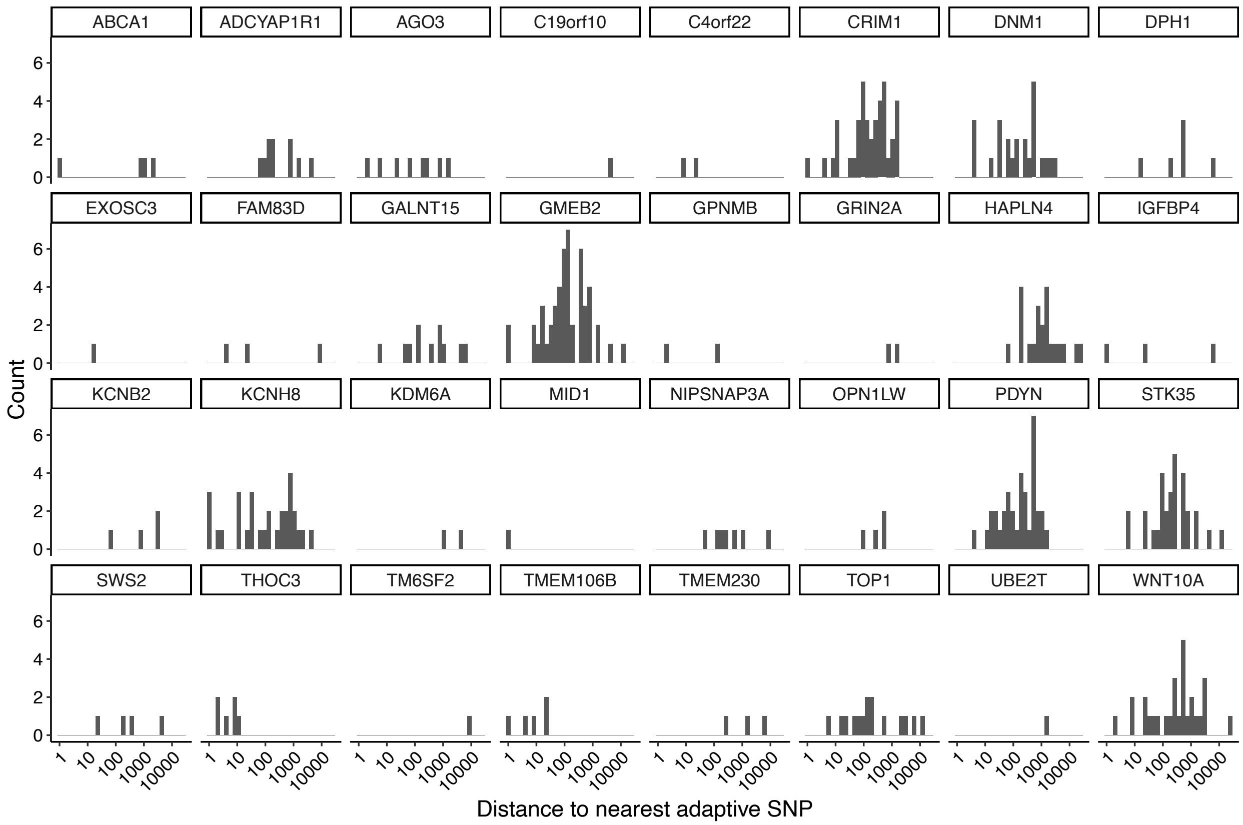

Figure S5. Adaptative SNPs associated with the same gene are generally spaced apart.

Distribution of the distance to nearest adaptive SNP within a gene. Note the log scale of the x-axis.

bioRxiv preprint doi: https://doi.org/10.1101/2025.11.17.688971; this version posted December 27, 2025. The copyright holder for this preprint (which was not certified by peer review) is the author/funder, who has granted bioRxiv a license to display the preprint in perpetuity. It is made available under aCC-BY 4.0 International license.

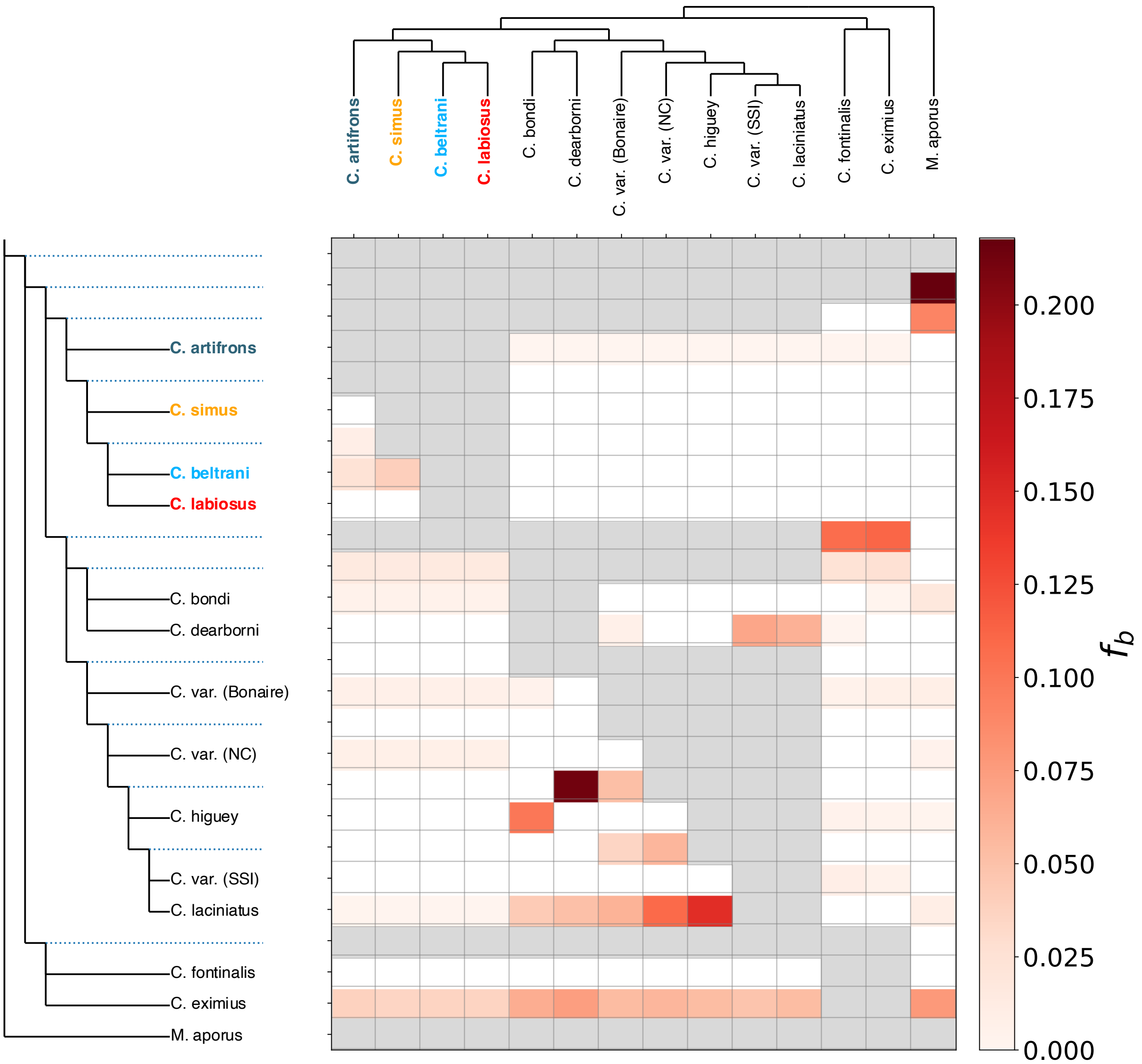

Figure S6. *f*-branch (f_b) show that introgression with desert pupfish occurred before the Chichancanab radiation. Boxes with red color represent significant introgression between P3 species (top) and the species/internal branch on the left. For visualization, non-significant f_b were changed to zero and are completely white boxes. Grey boxes represent comparisons that cannot be made, as f_b cannot be calculated for introgression between sister taxon. Dashed lines on the left are internal branches. SSI: San Salvador Island, Bahamas; NC: North Carolina, USA.

bioRxiv preprint doi: https://doi.org/10.1101/2025.11.17.688971; this version posted December 27, 2025. The copyright holder for this preprint (which was not certified by peer review) is the author/funder, who has granted bioRxiv a license to display the preprint in perpetuity. It is made available under aCC-BY 4.0 International license.

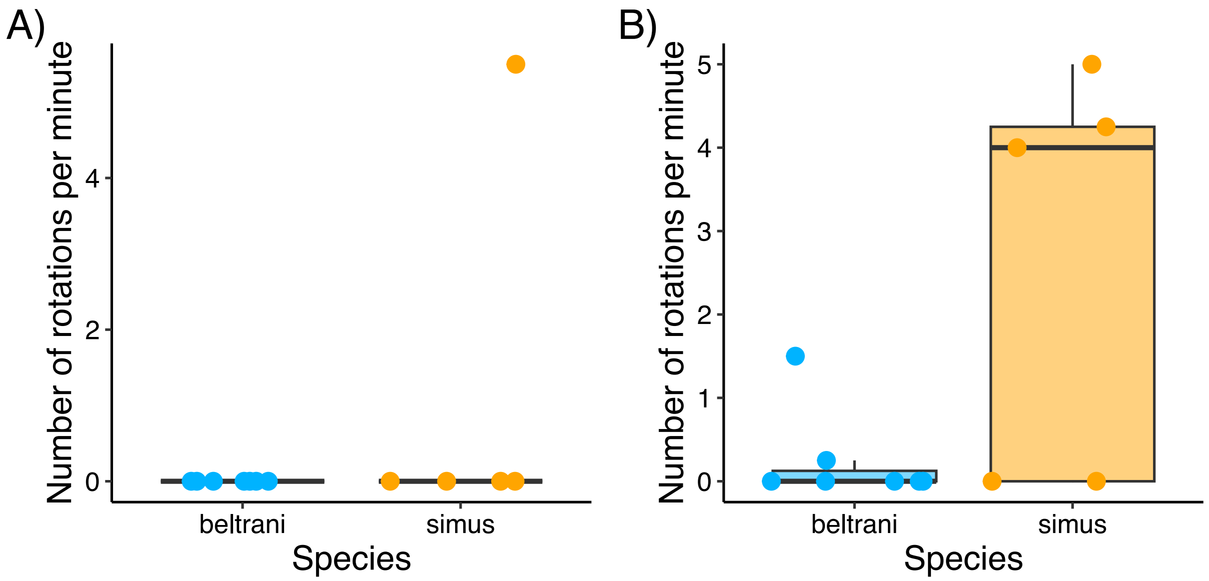

Figure S7. **Both species did not have a strong response to either control scenario in the optomotor response experiment. (A)** Number of complete rotations fish swam when the machine was off and **(B)** when the machine was on but the apparatus was all black**.** Boxplots and points of the number of complete rotations individuals performed during the “off-banded” portion of the experiment. Note that some boxplots are compressed to a single line because most individuals had no response.

bioRxiv preprint doi: https://doi.org/10.1101/2025.11.17.688971; this version posted December 27, 2025. The copyright holder for this preprint (which was not certified by peer review) is the author/funder, who has granted bioRxiv a license to display the preprint in perpetuity. It is made available under aCC-BY 4.0 International license.

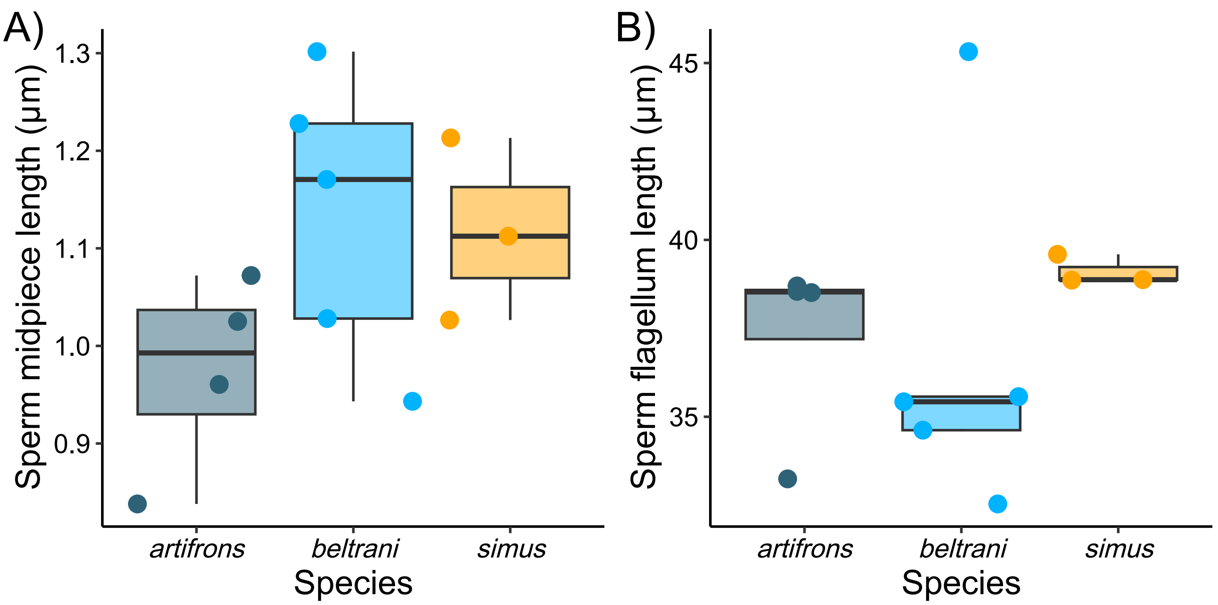

Figure S8. **Species do not differ in sperm midpiece size or sperm flagellum length.** Boxplots and points of the mean sperm morphological estimate for an individual.

bioRxiv preprint doi: https://doi.org/10.1101/2025.11.17.688971; this version posted December 27, 2025. The copyright holder for this preprint (which was not certified by peer review) is the author/funder, who has granted bioRxiv a license to display the preprint in perpetuity. It is made available under aCC-BY 4.0 International license.

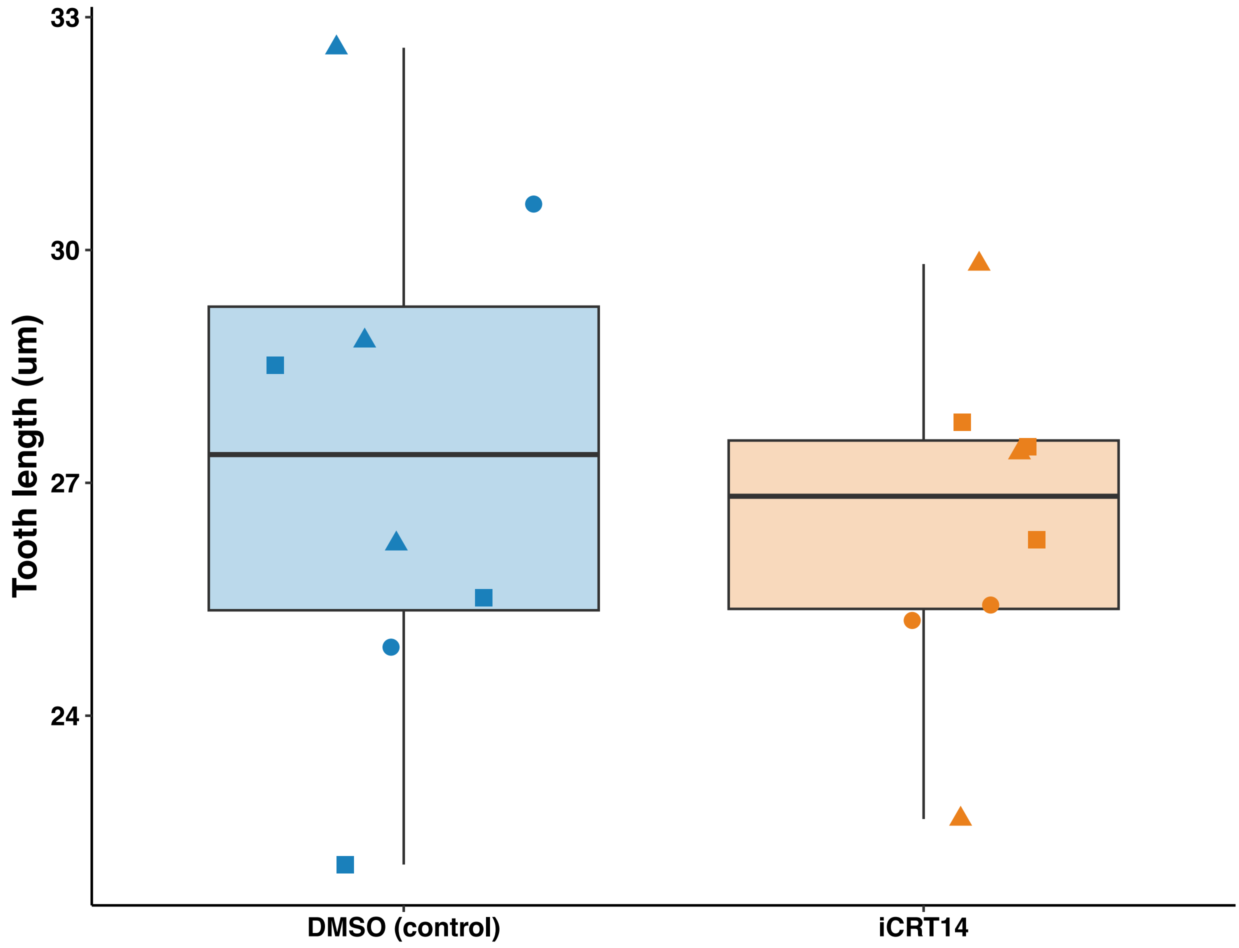

Figure S9. Tooth length was not affected by Wnt inhibition. Boxplot and jittered points of average tooth length of the left and right teeth closest to the mandibular symphysis for *C. beltrani* larvae treated with either a control or Wnt inhibitor (iCRT14). Shapes represent different split-brood replicates.

bioRxiv preprint doi: https://doi.org/10.1101/2025.11.17.688971; this version posted December 27, 2025. The copyright holder for this preprint (which was not certified by peer review) is the author/funder, who has granted bioRxiv a license to display the preprint in perpetuity. It is made available under aCC-BY 4.0 International license.

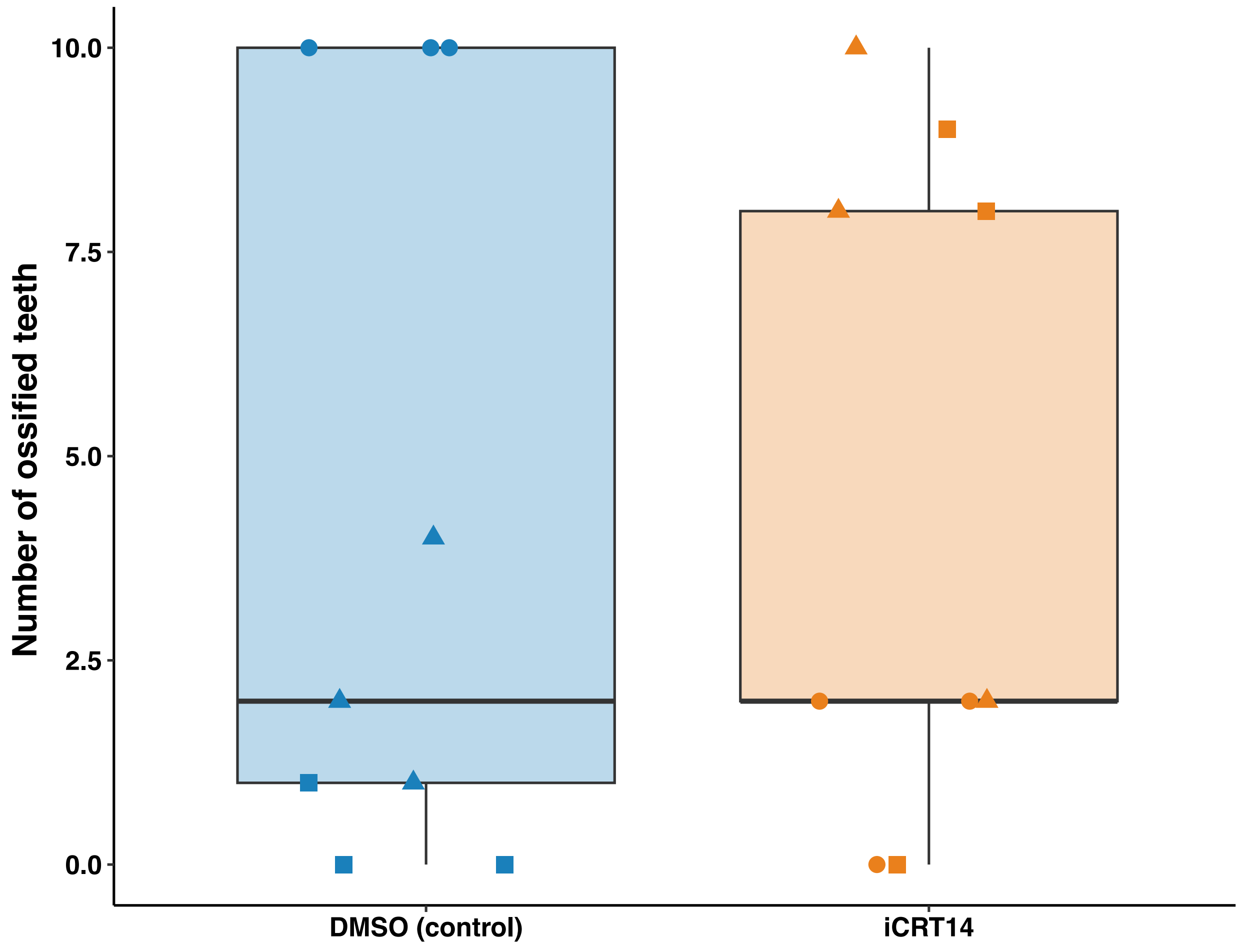

Figure S10. Number of ossified teeth was not affected by Wnt inhibition. Boxplot and jittered points of number of ossified teeth in *C. beltrani* larvae treated with either a control or Wnt inhibitor (iCRT14). Shapes represent different split-brood replicates.

bioRxiv preprint doi: https://doi.org/10.1101/2025.11.17.688971; this version posted December 27, 2025. The copyright holder for this preprint (which was not certified by peer review) is the author/funder, who has granted bioRxiv a license to display the preprint in perpetuity. It is made available under aCC-BY 4.0 International license.

SI References:

1. S. Chen, Y. Zhou, Y. Chen, J. Gu, fastp: an ultra-fast all-in-one FASTQ preprocessor. *Bioinformatics* **34**, i884–i890 (2018).

2. H. Li, Aligning sequence reads, clone sequences and assembly contigs with BWA-MEM. [Preprint] (2013). Available at: http://arxiv.org/abs/1303.3997.

3. A. McKenna, *et al.*, The Genome Analysis Toolkit: A MapReduce framework for analyzing next-generation DNA sequencing data. *Genome Res.* **20**, 1297–1303 (2010).

4. K. Okonechnikov, A. Conesa, F. García-Alcalde, Qualimap 2: advanced multi-sample quality control for high-throughput sequencing data. *Bioinformatics* **32**, 292–294 (2016).

5. M. A. DePristo, *et al.*, A framework for variation discovery and genotyping using next-generation DNA sequencing data. *Nat. Genet.* **43**, 491–498 (2011).

6. G. A. Van der Auwera, *et al.*, From FastQ Data to High-Confidence Variant Calls: The Genome Analysis Toolkit Best Practices Pipeline. *Curr. Protoc. Bioinform.* **43**, 11.10.1-11.10.33 (2013).

7. D. Caetano-Anolles, GenotypeGVCFs and the death of the dot (obsolete as of GATK 4.6.0.0). *GATK* (2024). Available at: https://gatk.broadinstitute.org/hc/en-us/articles/6012243429531-GenotypeGVCFs-and-the-death-of-the-dot-obsolete-as-of-GATK-4-6-0-0.

8. P. Danecek, *et al.*, Twelve years of SAMtools and BCFtools. *Gigascience* **10**, giab008 (2021).

9. S. Purcell, *et al.*, PLINK: A Tool Set for Whole-Genome Association and Population-Based Linkage Analyses. *Amer. J. Hum. Genet.* **81**, 559–575 (2007).

10. D. H. Alexander, J. Novembre, K. Lange, Fast model-based estimation of ancestry in unrelated individuals. *Genome Res.* **19**, 1655–1664 (2009).

11. S. Schiffels, K. Wang, "MSMC and MSMC2: The Multiple Sequentially Markovian Coalescent" in *Statistical Population Genomics*, J. Y. Dutheil, Ed. (Springer US, 2020), pp. 147–166.

12. E. J. Richards, *et al.*, A vertebrate adaptive radiation is assembled from an ancient and disjunct spatiotemporal landscape. *Proc. Natl. Acad. Sci. U.S.A.* **118**, e2011811118 (2021).

13. P. Danecek, *et al.*, The variant call format and VCFtools. *Bioinformatics* **27**, 2156–2158 (2011).

bioRxiv preprint doi: https://doi.org/10.1101/2025.11.17.688971; this version posted December 27, 2025. The copyright holder for this preprint (which was not certified by peer review) is the author/funder, who has granted bioRxiv a license to display the preprint in perpetuity. It is made available under aCC-BY 4.0 International license.

14. J. M. Schmidt, M. de Manuel, T. Marques-Bonet, S. Castellano, A. M. Andrés, The impact of genetic adaptation on chimpanzee subspecies differentiation. *PLOS Genet.* **15**, e1008485 (2019).

15. M. Shpak, K. N. Lawrence, J. E. Pool, The precision and power of population branch statistics in identifying the genomic signatures of local adaptation. *Genome Biol. Evol.* **17**, evaf080 (2025).

16. M. Malinsky, M. Matschiner, H. Svardal, Dsuite - Fast D-statistics and related admixture evidence from VCF files. *Mol. Ecol. Res.* **21**, 584–595 (2021).

17. S. G. Hernández-Ávila, C. W. Hoagstrom, W. A. Matamoros, Historical biogeography of North American killifishes (Cyprinodontiformes) recapitulates geographical history in the Gulf of México watershed. *Zool J. Linn. Soc.* **202**, zlae105 (2024).

18. E. Y. Durand, N. Patterson, D. Reich, M. Slatkin, Testing for ancient admixture between closely related populations. *Mol. Biol. Evol.* **28**, 2239–2252 (2011).

19. B. Pfeifer, D. D. Kapan, Estimates of introgression as a function of pairwise distances. *BMC Bioinform.***20**, 207 (2019).

20. C. Feng, J. Wang, A. Liston, M. Kang, Recombination variation shapes phylogeny and introgression in wild diploid strawberries. *Mol. Biol. Evol.* **40**, msad049 (2023).

21. A. Morales-Cruz, *et al.*, Introgression among North American wild grapes (*Vitis*) fuels biotic and abiotic adaptation. *Genome Biol.* **22**, 254 (2021).

22. A. R. Quinlan, I. M. Hall, BEDTools: a flexible suite of utilities for comparing genomic features. *Bioinformatics* **26**, 841–842 (2010).

23. P. Cingolani, *et al.*, A program for annotating and predicting the effects of single nucleotide polymorphisms, SnpEff: SNPs in the genome of *Drosophila melanogaster* strain w1118; iso-2; iso-3. *Fly* **6**, 80–92 (2012).

24. J. Smith, G. Coop, M. Stephens, J. Novembre, Estimating time to the common ancestor for a beneficial allele. *Mol. Biol. Evol.* **35**, 1003–1017 (2018).

25. M. Schumer, *et al.*, Natural selection interacts with recombination to shape the evolution of hybrid genomes. *Science* **360**, 656–660 (2018).

26. S. C. F. Neuhauss, *et al.*, Genetic disorders of vision revealed by a behavioral screen of 400 essential loci in zebrafish. *J. Neurosci.* **19**, 8603–8615 (1999).

27. C. T. Rueden, *et al.*, ImageJ2: ImageJ for the next generation of scientific image data. *BMC Bioinform.* **18**, 529 (2017).

28. D. Bates, M. Mächler, B. Bolker, S. Walker, Fitting linear mixed-effects models using lme4. *J. Stat. Softw.* **67**, 1–48 (2015).

bioRxiv preprint doi: https://doi.org/10.1101/2025.11.17.688971; this version posted December 27, 2025. The copyright holder for this preprint (which was not certified by peer review) is the author/funder, who has granted bioRxiv a license to display the preprint in perpetuity. It is made available under aCC-BY 4.0 International license.

29. R. V. Lenth, *emmeans: Estimated Marginal Means, aka Least-Squares Means* (2021).

30. M. Walker, C. Kimmel, A two-color acid-free cartilage and bone stain for zebrafish larvae. *Biotech. Histochem.* **82**, 23–28 (2007).

31. R. Koita, S. Oikawa, T. Tani, M. Matsuda, A. Kawamura, Live visualization of calcified bones in zebrafish and medaka larvae and juveniles using calcein and alizarin red S. *Bio-protocol* **14** (2024).

32. Y. Miyamoto, *et al.*, The STK35 locus contributes to normal gametogenesis and encodes a lncRNA responsive to oxidative stress. *Biol. Open* **7**, bio032631 (2018).

33. C.S. Jayasena, M.E. Bronner, Rbms3 functions in craniofacial development by posttranscriptionally modulating TGF-β signaling. *J. Cell. Biol.* **199**(2012)

34. H. Li, Y. Dai, Z. Luo, D. Nie, Cloning of a new testis-enriched gene C4orf22 and its role in cell cycle and apoptosis in mouse spermatogenic cells. *Mol. Biol. Rep.* **46**, 2029–2038 (2019).

35. P.M. Van Laarhoven, Kabuki syndrome genes KMT2D and KDM6A: functional analyses demonstrate critical roles in craniofacial, heart and brain development. *Hum. Mol. Genet.* **24**, (2015).

36. R. Maier, *et al.*, On the limits of fitting complex models of population history to f-statistics. *eLife* **12**, e85492 (2023).
