# Supplementary material for: Parallel and non-parallel features of adaptive radiation in Yucatán pupfishes
Source: bioRxiv. 2025 Nov 18:2025.11.17.688971. Preprint. [Version 1] doi: 10.1101/2025.11.17.688971 (PMC12667942; doi:10.1101/2025.11.17.688971)
Supplement: Supplementary file 1 [file NIHPP2025.11.17.688971v1-supplement-1.pdf]

bioRxiv preprint doi: https://doi.org/10.1101/2025.11.17.688971; this version posted November 18, 2025. The copyright holder for this preprint (which was not certified by peer review) is the author/funder, who has granted bioRxiv a license to display the preprint in perpetuity. It is made available under aCC-BY 4.0 International license.

Supplementary materials

Table S1. Mean genome-wide pairwise F_{ST} statistics within the Chichancanab radiation and with sister outgroup (*artifrons*).

	labiosus	***beltrani***	***artifrons***
simus	0.090	0.068	0.182
labiosus		0.032	0.184
beltrani			0.168

Table S2. F_{ST} statistics within the Chichancanab radiation.

**Comparison**	**F_{ST} = 1 (fixed)**	**1> F_{ST} ≥ 0.95 (nearly fixed)**	**0.95> F_{ST} ≥ 0.9**	**0.9> F_{ST} ≥0.8**	**Other**	**Mean F_{ST}**
simus vs beltrani + labiosus	19	1127	3304	20173	10359605	0.07
labiosus vs *beltrani + simus*	0	0	3	94	10384131	0.04
beltrani vs *simus + labiosus*	0	0	0	13	10384215	0.02

Table S3. Fixed SNPs between *C. simus* and *C. beltrani* + *C. labiosus*. Bold rows indicate SNPs within top 1% of PBS windows. Bolded genes only are genes that have regions that fall within the top 1% PBS percentile.

Position	Gene	Distance to gene	PBS percentile	Annotation
Chr 1: 5290600	**STK35**	12594	1.85	
Chr 1:5290602	**STK35**	12592	1.85	Fertility, eye development and oxidative stress (Miyamoto et al 2018).
Chr 1:5290604	**STK35**	12590	1.85	
Chr 2: 17498913	SLC17A8	Intron	1.24	Glutamate neurotransmitter, inner hair cell dysfunction
Chr 3: **11687002**	**AGO3**	**Intron**	**0.34**	**RNA regulator**
Chr 3: 32286614	RBMS3	14363	22.93	
Chr 3: 32286627	RBMS3	14350	22.93	Involved in chondrogenic craniofacial development of zebrafish
Chr 3: 32286638	RBMS3	14339	22.93	(Jayasena and Bronner 2012).
Chr 3: 32286614	RBMS3	14363	22.93	
Chr 11: 27324192	GRIA3	Intron	2.85	
Chr 11: 27336469	GRIA3	2772	3.18	Glutamate receptor strongly expressed in zebrafish retina
Chr 11: 27336472	GRIA3	2775	3.18	development.
Chr 11: 27336476	GRIA3	2779	3.18	
Chr 11: **28255536**	**NA**	**3026**	**0.98**	**NA**
Chr 11: **28255616**	**NA**	**3106**	**0.98**	**NA**
Chr 11: **28256343**	**NA**	**3833**	**0.98**	**NA**
Chr 11: **28257367**	**NA**	**4857**	**0.98**	**NA**
Chr 11: 28260047	NA	7537	2.87	NA
Chr 12: 13854139	**C4orf22**	11903	1.30	Spermatogenesis, cilia, flagellum
Chr 24: **12693158**	**KDM6A**	**Intron**	**0.05**	**Kabuki syndrome (craniofacial development)**

bioRxiv preprint doi: https://doi.org/10.1101/2025.11.17.688971; this version posted November 18, 2025. The copyright holder for this preprint (which was not certified by peer review) is the author/funder, who has granted bioRxiv a license to display the preprint in perpetuity. It is made available under aCC-BY 4.0 International license.

Table S4. Summary statistics of the distance between closest adaptive candidate SNPs within the same gene

Gene	Median Distance	Mean Distance	SD	
TM6SF2	7430	7430	NA	2
C19orf10	4789	4789	NA	2
KDM6A	2892.5	2892.5	2410.52702	3
KCNB2	1803	1738.75	1614.92299	5
UBE2T	1742	1742	NA	2
TMEM230	1734	3039	3599.52427	4
ABCA1	1037.5	1097.5	959.375665	5
HAPLN4	974	1608.21739	9927.05273	24
GRIN2A	904	1270.6667	4273.38008	4
DPH1	608	1224.5	1999.25934	7
WNT10A	402	1442.17857	3986.39626	29
OPN1LW	386.5	359.5	225.229513	5
NIPSNAP3A	253	1451.71429	2819.19065	8
GALNT15	246.5	675.166667	3156.86746	13
SWS2	246	1381.25	2423.03892	5
STK35	217	793.366667	1992.8543	31
CRIM1	201	47.829268	2176.05381	42
PDYN	187	326.371429	355.925561	36
ADCYAP1R1	186.5	833	1304.35501	11
DNM1	186.5	459.708333	677.261781	25
DNM1	186.5	459.708333	677.261781	25
KCNH8	131.5	584.366667	1030.65532	31
TOP1	124	332.375	6687.63393	17
AGO3	115	357.625	552.097284	9
GMEB2	112	319.490196	2485.05809	52
GPNMB	57	57	77.781746	3
IGFBP4	26	2254	3880.67997	4
FAM83D	23	2523.66667	4347.74658	4
EXOSC3	17	17	NA	2
C4orf22	15	15	9.899495	3
TMEM106B	7	11.4	10.358571	6
THOC3	6	5.833333	3.600926	7
MID1	1	1	NA	2

bioRxiv preprint doi: https://doi.org/10.1101/2025.11.17.688971; this version posted November 18, 2025. The copyright holder for this preprint (which was not certified by peer review) is the author/funder, who has granted bioRxiv a license to display the preprint in perpetuity. It is made available under aCC-BY 4.0 International license.

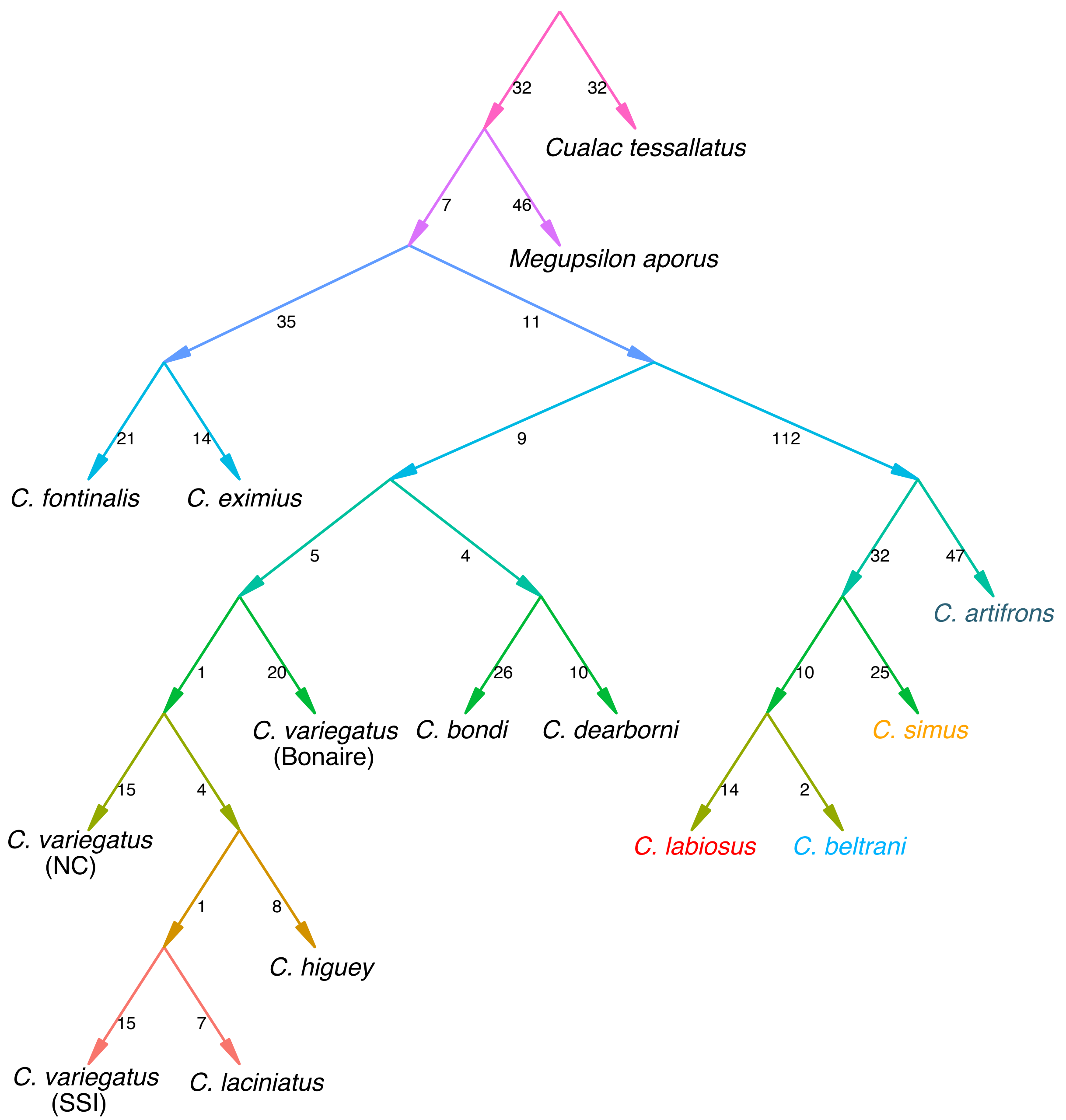

Figure S1. Best fitting admixture graph assuming zero admixture events. Admixture graph was found using the *findGraphs* function in *ADMIXTOOLS2* (Maier et al., 2023). Number on edges represent drift weights.

bioRxiv preprint doi: https://doi.org/10.1101/2025.11.17.688971; this version posted November 18, 2025. The copyright holder for this preprint (which was not certified by peer review) is the author/funder, who has granted bioRxiv a license to display the preprint in perpetuity. It is made available under aCC-BY 4.0 International license.

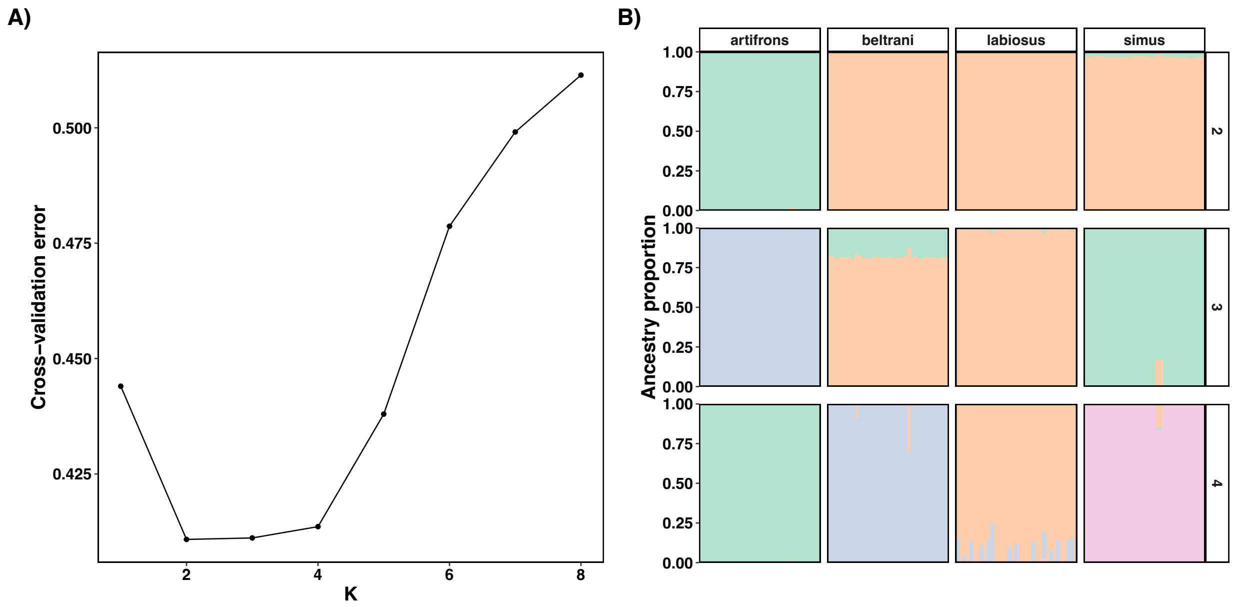

Figure S2. ADMIXTURE results support genetic differentiation of Yucatán Cyprinodon pupfish species. (A) Cross-validation error for different values of K clusters, equally support 2, 3, and 4 clusters. (B) ADMIXTURE plots showing the clusters at different values of K.

bioRxiv preprint doi: https://doi.org/10.1101/2025.11.17.688971; this version posted November 18, 2025. The copyright holder for this preprint (which was not certified by peer review) is the author/funder, who has granted bioRxiv a license to display the preprint in perpetuity. It is made available under aCC-BY 4.0 International license.

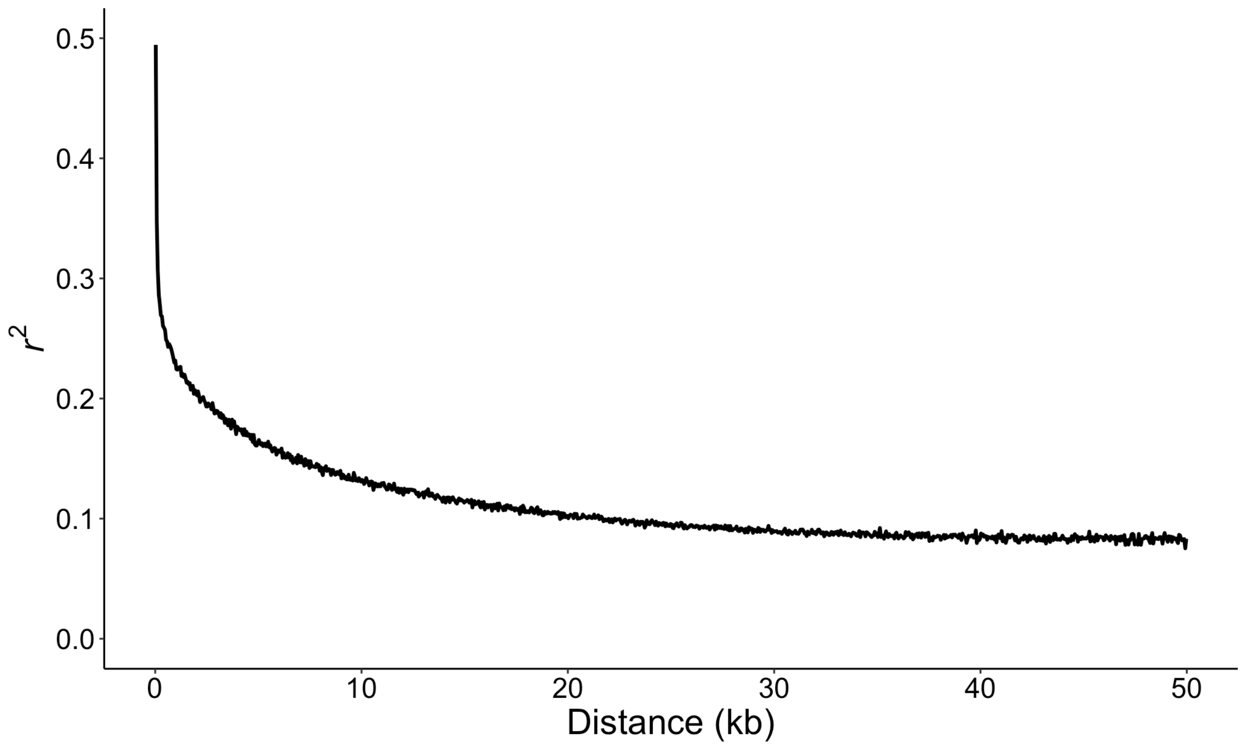

Figure S3. Linkage decay occurs rapidly in *Cyprinodon simus*. Linkage decay was calculated in windows of 1000KB using PLINK (v1.9). The results graphed are a random sample of all calculated r^2 values taken for plotting purposes (n = 9,645 r^2 values).

bioRxiv preprint doi: https://doi.org/10.1101/2025.11.17.688971; this version posted November 18, 2025. The copyright holder for this preprint (which was not certified by peer review) is the author/funder, who has granted bioRxiv a license to display the preprint in perpetuity. It is made available under aCC-BY 4.0 International license.

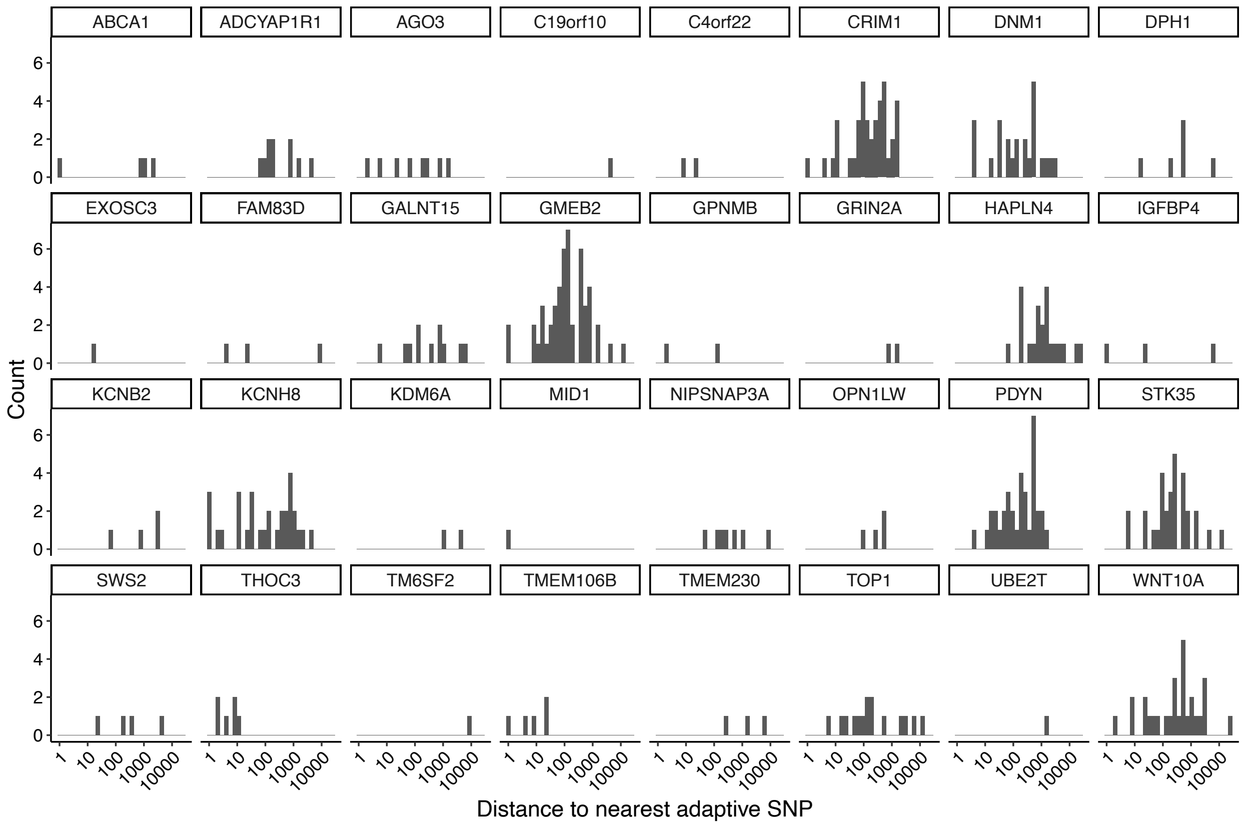

Figure S4. Adaptative SNPs associated with the same gene are generally spaced apart.

Distribution of the distance to nearest adaptive SNP within a gene. Note the log scale of the x-axis.

bioRxiv preprint doi: https://doi.org/10.1101/2025.11.17.688971; this version posted November 18, 2025. The copyright holder for this preprint (which was not certified by peer review) is the author/funder, who has granted bioRxiv a license to display the preprint in perpetuity. It is made available under aCC-BY 4.0 International license.

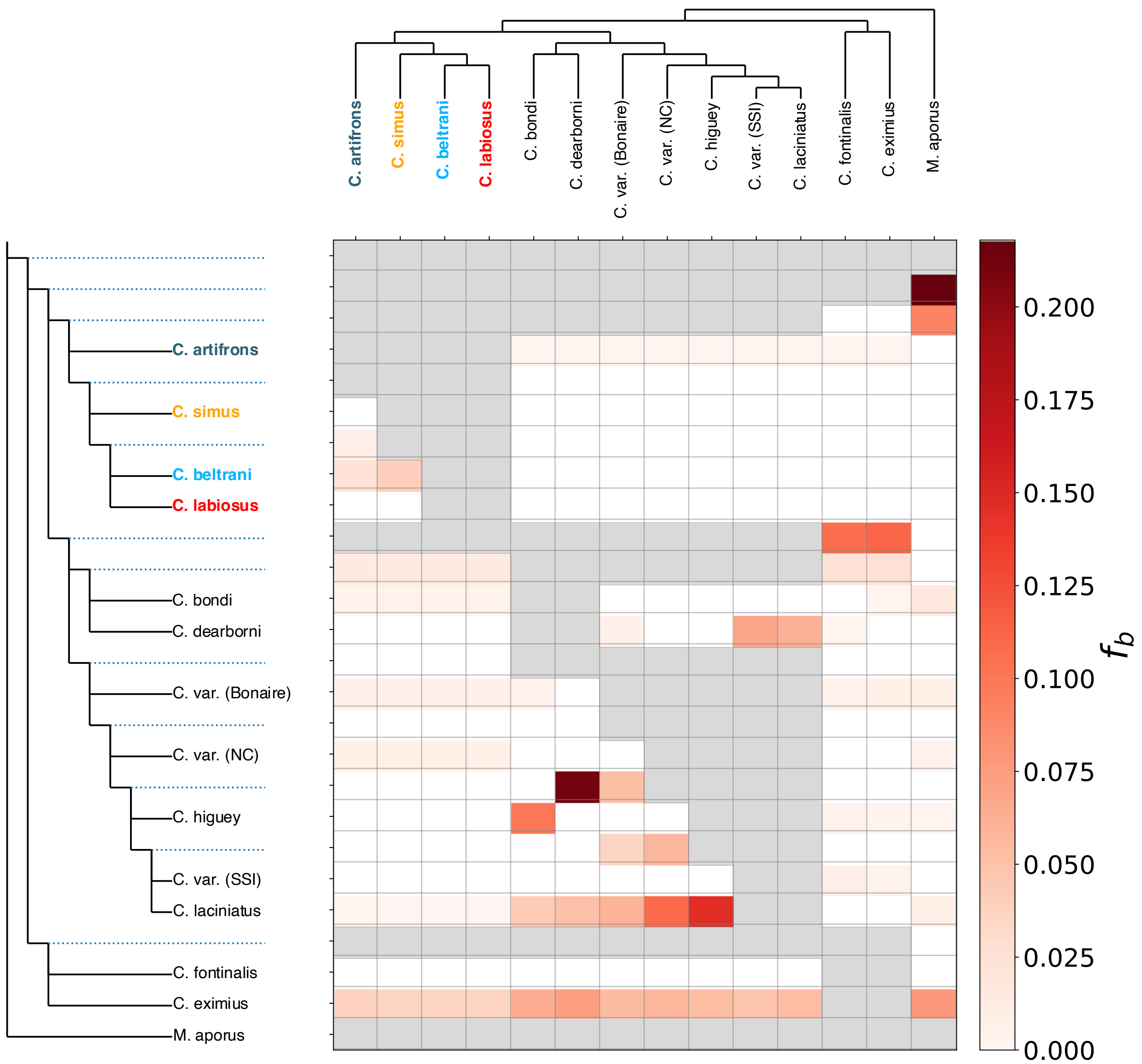

Figure S5. *f*-branch (f_b) show that introgression with desert pupfish occurred before the Chichancanab radiation. Boxes with red color represent significant introgression between P3 species (top) and the species/internal branch on the left. For visualization, non-significant f_b were changed to zero and are completely white boxes. Grey boxes represent comparisons that cannot be made, as f_b cannot be calculated for introgression between sister taxon. Dashed lines on the left are internal branches. SSI: San Salvador Island, Bahamas; NC: North Carolina, USA.

bioRxiv preprint doi: https://doi.org/10.1101/2025.11.17.688971; this version posted November 18, 2025. The copyright holder for this preprint (which was not certified by peer review) is the author/funder, who has granted bioRxiv a license to display the preprint in perpetuity. It is made available under aCC-BY 4.0 International license.

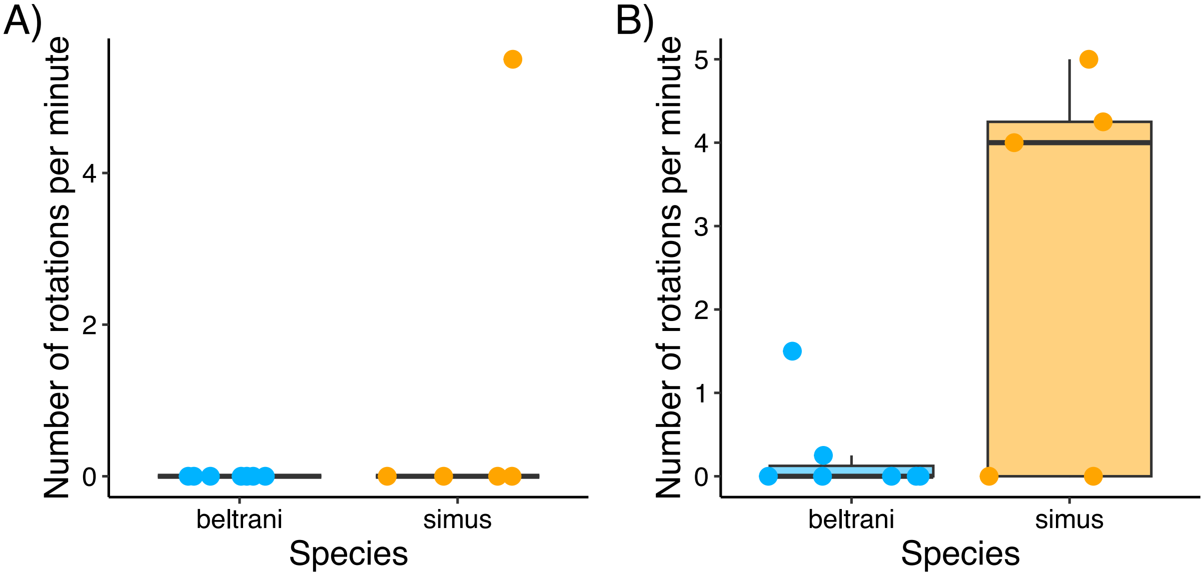

Figure S6. **Both species did not have a strong response to either control scenario in the optomotor response experiment. (A)** Number of complete rotations fish swam when the machine was off and **(B)** when the machine was on but the apparatus was all black**.** Boxplots and points of the number of complete rotations individuals performed during the “off-banded” portion of the experiment. Note that some boxplots are compressed to a single line because most individuals had no response.

bioRxiv preprint doi: https://doi.org/10.1101/2025.11.17.688971; this version posted November 18, 2025. The copyright holder for this preprint (which was not certified by peer review) is the author/funder, who has granted bioRxiv a license to display the preprint in perpetuity. It is made available under aCC-BY 4.0 International license.

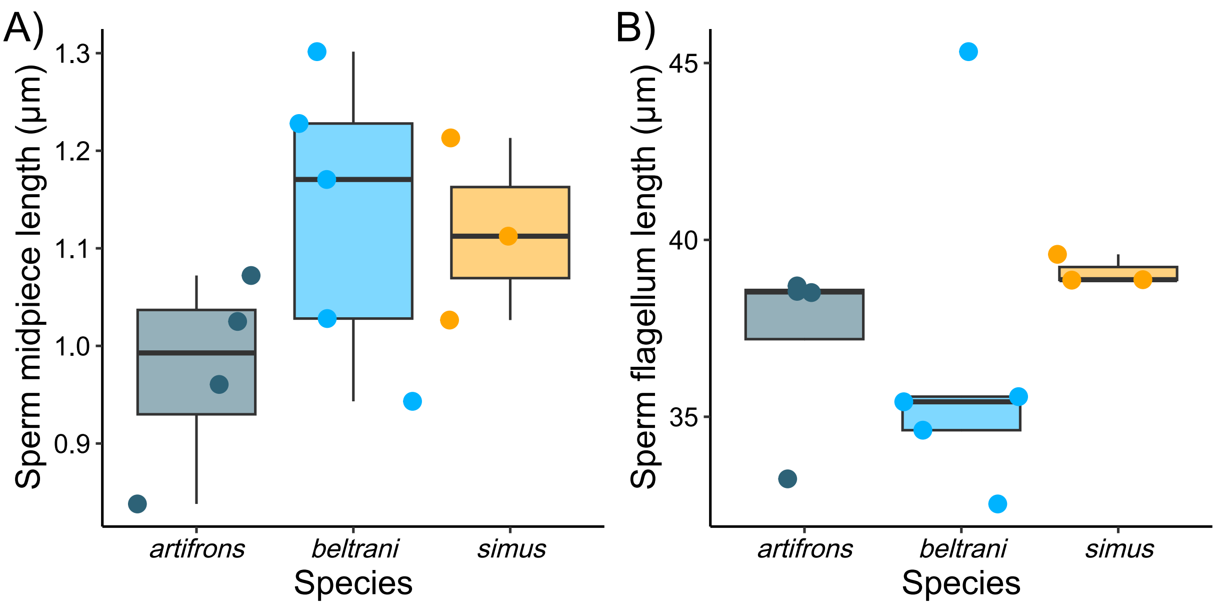

Figure S7. **Species do not differ in sperm midpiece size or sperm flagellum length.** Boxplots and points of the mean sperm morphological estimate for an individual.
